# Pain mechanisms in complex regional pain syndrome: a systematic review and meta-analysis of quantitative sensory testing outcomes

**DOI:** 10.1186/s13018-022-03461-2

**Published:** 2023-01-02

**Authors:** Mohamed Gomaa Sobeeh, Karima Abdelaty Hassan, Anabela Gonçalves da Silva, Enas Fawzy Youssef, Nadia Abdelazim Fayaz, Maha Mostafa Mohammed

**Affiliations:** 1 Department of Physical Therapy for Musculoskeletal Disorders and its Surgeries, Faculty of Physical Therapy, Cairo University, Giza, Egypt; 2 Faculty of Physical Therapy, Sinai University, Ismailia, Egypt; 3 CINTESIS.UA@RISE, School of Health Sciences, University of Aveiro, Aveiro, Portugal

**Keywords:** Complex regional pain syndrome, Sensory profile, Pain mechanisms, Quantitative sensory testing

## Abstract

**Background:**

Complex regional pain syndrome (CRPS) is a chronic condition following inciting events such as fractures or surgeries with sensorimotor and autonomic manifestations and poor prognosis. This review aimed to provide conclusive evidence about the sensory phenotype of CRPS based on quantitative sensory testing (QST) to understand the underlying pain mechanisms and guide treatment strategies.

**Databases:**

Eight databases were searched based on a previously published protocol. Forty studies comparing QST outcomes (thermal, mechanical, vibration, and electric detection thresholds, thermal, mechanical, pressure, and electric pain thresholds, wind-up ratio, mechanical pain sensitivity, allodynia, flare area, area after pinprick hyperalgesia, pleasantness after C-tactile stimulation, and pain ratings) in chronic CRPS (adults and children) versus healthy controls were included.

**Results:**

From 37 studies (14 of low quality, 22 of fair quality, and 1 of good quality), adults with CRPS showed: (i) significant loss of thermal, mechanical, and vibration sensations, significant gain of thermal and mechanical pain thresholds, significant elevation of pain ratings, and no difference in wind-up ratio; (ii) significant reduction of pleasantness levels and increased area of pinprick hyperalgesia, in the affected limb. From three fair-quality studies, adolescents and children with CRPS showed loss of cold detection with cold hyperalgesia in the affected limb. There was moderate to substantial overall heterogeneity.

**Conclusion:**

Diffuse thermal and mechanical hypoesthesia with primary and secondary hyperalgesia, enhanced pain facilitation evidenced by increased area of pinprick hyperalgesia, and elevated pain ratings are dominant in adults with CRPS. Adolescents and children with CRPS showed less severe sensory abnormalities.

**Supplementary Information:**

The online version contains supplementary material available at 10.1186/s13018-022-03461-2.

## Introduction

Complex regional pain syndrome (CRPS) is a chronic debilitating pain condition of the limbs following trauma or surgery with an incidence rate of 26.2 per 100,000 person-years [1, 2]**.** CRPS occurs commonly in elderly people, in females more than males, and the upper extremity more than in the lower extremity [2]. Two main types of CRPS were identified: CRPS types 1 and 2 [3]. CRPS type 1 or reflex sympathetic dystrophy is characterized by sensory, motor, and autonomic abnormalities without electrophysiological evidence of nerve lesion. On contrary, CRPS type 2 is characterized by identifiable nerve lesions that can be detected through electrophysiological findings and it is considered typical neuropathic pain [1].

CRPS is, usually, associated with poor outcomes, long-term complaints, and comorbidities (e.g., depression and photophobia) [4–6]; however, the pain mechanisms involved in CRPS are not fully understood. [7]. Neurogenic inflammation, peripheral sensitization (PS), central sensitization (CS), small nerve fiber pathology, autonomic dysregulation, and psychological states represent the shared model of the underlying pathophysiology of CRPS [8–12]. Neurogenic inflammation is caused by neuropeptides released from the primary afferents resulting in axon reflex vasodilatation and protein extravasation [8]. PS is defined as enhanced responsiveness and decreased threshold of nociceptive neurons within the afflicted receptive field, and it was demonstrated in CRPS by the presence of primary hyperalgesia in the affected regions [13]. Signs of PS in CRPS can include gain of thermal and mechanical pain thresholds at the affected sites [14–16]**.**

In CRPS, secondary hyperalgesia in distant locations away from the affected area was found to be indicative of CS, which is an increased response of nociceptive neurons in the central nervous system to normal or sub-threshold afferent input [17]. Signs of CS in CRPS can include widespread gain of thermal and mechanical pain thresholds, enhanced pain facilitation as evidenced by elevated pain ratings, and/or impaired pain inhibition [14, 18].

It has been demonstrated that CRPS patients have a bilateral reduction in intraepidermal small nerve fiber density, and these fibers are responsible for nociception and perceiving temperature [19]. Conceivably, reduction of the small nerve fiber density would be responsible for altered perception of these sensations. Autonomic dysregulation could result in enhanced pain perception as evidenced by increased expression of α1-adrenergic receptors [11]. Also, post-traumatic stress disorder and pain catastrophizing seem to increase pain response in CRPS [12].

A valid and standardized tool to assess pain mechanisms involved in different chronic pain conditions (inflammatory, neuropathic, and mixed chronic pain conditions) is quantitative sensory testing (QST) [20]. As far as we are aware, this is the first review to consolidate and evaluate the QST data of affected areas and remote areas away from the affected site in adults and children with CRPS type 1 compared to healthy controls. Additionally, we analyzed a broad range of variables including flare area after induction of noxious stimulus, pain area after pinprick induced hyperalgesia, pain ratings after noxious thermal stimulus, electric pain threshold, current perception thresholds, and pleasantness levels after C-tactile perception in an attempt to reach more conclusive results on the sensory profile and pain mechanisms of CRPS type 1.

## Methods

### Protocol registration

The review protocol was registered as an a priori study at the International Prospective Register of Systematic Reviews (PROSPERO) (registration number: CRD42021237157) and we used PRISMA guidelines (www.prisma-statement.org) to report this review.

### Eligibility criteria

Studies were included if they (1) compared adults (age ≥ 18 years) or adolescents and children (age < 18 years) with CRPS type 1 (symptoms duration ≥ 8 weeks) to healthy controls, (2) diagnosed CRPS type 1 (unilateral or bilateral) through clinical assessment and the International Association for the Study of Pain (IASP) or the Budapest criteria, (3) investigated any modality of QST, flare areas after noxious stimulus, conditioned pain modulation, pain rating after noxious stimulus, and pain area after induced pinprick hyperalgesia, and (4) were written in English. We excluded studies that combined results of sensory testing of CRPS with other neuropathic conditions and studies that used the unaffected side as the control site. Additionally, we focused on the QST outcomes for CRPS type 1 only, which is a deviation from the previously published protocol. The protocol stated that both the QST outcomes for CRPS type 1 and type 2 would be included. However, a meta-analysis requires at least two studies, and we found one study only on CRPS type 2 that met the eligibility criteria [15]. Also, there is an identifiable nerve lesion in CRPS type 2 but not in CRPS type 1, which precludes including studies on CRPS type 2 and 1 in the same meta-analysis as that would prevent us from reaching a comprehensive understanding of the sensory profile and type of pain present in such a complex syndrome.

The main included parameters to study the sensory profile of CRPS type 1 were (1) detection thresholds including warm detection threshold (WDT), cold detection threshold (CDT), thermal sensory limen (TSL), vibration detection threshold (VDT), and mechanical detection threshold (MDT); (2) pain thresholds including heat pain threshold (HPT), cold pain threshold (CPT), pressure pain threshold (PPT), and mechanical pain threshold (MPT); (3) temporal summation or wind up ratio (WUR); (4) conditioned pain modulation (CPM); (5) mechanical pain sensitivity (MPS); (6) dynamic mechanical allodynia (DMA)**;** (7) flare area; (8) pain area after pinprick induced hyperalgesia; (9) current perception threshold; (10) electric pain threshold; and (11) pain ratings after thermal and mechanical stimuli. The definition of each variable is included in Table 1 [21–24].

**Table 1:** Definitions of sensory testing included in the review

Sensory testing	Definition
Cold detection threshold	The minimum detectable amount of applied cold
Warm detection threshold	The minimum detectable amount of applied warmth
Thermal sensory limen	The interval between the minimum detectable amount of applied warm and cold
Mechanical detection threshold	The minimum amount of mechanical stimulation that can be detected or elicit pain
Vibration detection threshold	The minimum amount of vibration intensities needed to elicit vibration sensation
Cold pain threshold	The minimum amount of cold needed to elicit pain sensation
Heat pain threshold	The minimum amount of heat needed to elicit pain sensation
Mechanical pain threshold	Geometric mean of series of applied forces via pin prick stimulator of different intensities ranged from 8 to 512 mN
Pressure pain threshold	The minimum amount of pressure needed to elicit painful sensation
Mechanical pain sensitivity	Pain ratings after a series of mechanical stimuli that needed to elicit mechanical pain threshold
Wind-up ratio (temporal summation)	Numerical ratings within five trains of a single pinprick stimulus (a) divided by a series (b) of 10 repetitive pinprick stimuli. WUR is the ratio: b/a
Pain ratings after noxious stimulus	Pain ratings of thermal and mechanical thresholds that provoked pain
Area after pinprick hyperalgesia	Surface area of pain distribution after application of pinprick mechanical stimulus needed to elicit pain
Flare area after electric	
stimulus	Area of blood perfusion after application of an electrical stimulus, usually assessed through laser Doppler imaging
Electric pain threshold	The minimum amount of electric current needed to elicit pain
Current (electric) detection threshold	The minimum amount of detected electric current
Dynamic mechanical allodynia	Pathological sensory response to innocuous stimulus, usually assessed through application of cotton piece or foam brush
Paradoxical heat sensation	The perception of heat during rapid cooling of the skin
Conditioned pain modulation	The increase in thermal or mechanical pain thresholds after application of noxious stimulus in remote area away from the affected area. It represents the spatial assessor of endogenous pain modulation
Levels of pleasantness	The pleasantness level after application of stroking of velocity commonly ranged from 1 to 10 cm/s with C-tactile fibers are responsible for affective touch processing

### Search strategy and investigated databases

The main keywords of our search included complex regional pain syndrome, reflex sympathetic dystrophy, causalgia, central nervous system sensitization, hyperalgesia, quantitative sensory testing, conditioned pain modulation, hypoesthesia, wind-up ratio, mechanical hyperalgesia, temporal summation, thermal hyperalgesia, heat pain threshold, warm detection threshold, mechanical detection threshold, pressure pain threshold, allodynia, cold pain threshold, vibration detection threshold, cold detection threshold, mechanical pain sensitivity, mechanical pain threshold, thermal sensory limen, pain perception, electric pain threshold, current perception threshold, flare area, and laser Doppler imaging. Scopus, EMBASE, Web of Science, PubMed, EBSCO *host*, SAGE, Cochrane library, and ProQuest databases/search engines were searched from inception to January 2022 (Table 2). To identify other eligible articles, a manual search of references of the included studies was done.

**Table 2:** Search keywords

Database	Search keywords	Number of records
PubMed	(( "Central Sensitization" OR "Central Nervous System Sensitization" OR "sensitization" OR "CS" OR "pain sensitization" OR "hyperalges*" OR "hypesthes*" OR "hypoesthes*" OR "mechanical hyperalges*" OR "thermal hyperalges*" OR "mechanical allodyn*" OR "thermal allodyn*" OR "thermal threshold" OR "thermal detection threshold" OR "allodyn*" OR "temporal summation" OR "wind up" OR "wind-up ratio" OR "WUR" OR "pain threshold" OR "sensory threshold" OR "QST" OR "quantitative thermal testing" OR "quantitative sensory testing*" OR "sensation" OR "conditioned pain modulation" OR "CPM" OR "endogenous pain" OR "pressure pain threshold" OR "vibration detection threshold" OR "heat detection threshold" OR "WDT" OR "hot sensitiv*" OR "cold sensitiv*" OR "heat pain threshold" OR "HPT" OR "cold detection threshold" OR "CDT" OR "cold pain threshold" OR "CPT" OR "warm detection threshold" OR "mechanical pain threshold" OR "mechanical detection threshold" OR "mechanical pain sensitiv*" OR "PPT" OR "Pressure-pain threshold" OR "pain threshold" OR "sensory profile" OR "pain perception" OR "current perception threshold" OR "electric pain threshold" OR "pain tolerance threshold" OR "flare area" OR "laser doppler imaging")) AND (( "complex regional pain syndrome*" OR "complex regional pain syndrome type I" OR "complex regional pain syndrome type II" OR "causalgia" OR "reflex sympathetic dystroph*" OR "Type II Complex Regional Pain Syndrome" OR "CRPS Type II" OR "Sudeck's Atrophy*" OR "CRPS Type I*" OR "Shoulder Hand Syndrome*" OR "Algodystroph*" OR "CRPS" OR "CRPS-1" OR "CRPS-2")) Filters applied: Full text, English, Humans	543

*CRPS* complex regional pain syndrome

### Study selection

After removing duplicates, two independent researchers (M.G.S. and K.A.H) screened the titles and abstracts of the relevant retrieved articles. The same two researchers obtained the full-text versions of the relevant articles and assessed them against the eligibility criteria. Conflicts were solved by discussion until a consensus was reached.

### Risk of bias assessment

Two researchers (M.G.S. and K.A.H) independently used the Newcastle–Ottawa quality assessment scale (NOS) for case–control and cohort studies to perform the risk of bias assessment. Three aspects were evaluated through the NOS using a star rating system: the selection of the study groups, the comparability of the groups, and the ascertainment of the exposure or outcome of interest. Each aspect contains several items that can be scored with one star, except for comparability, which can score up to two stars (Table 3) [25]. The highest possible NOS score is 9. According to Agency for Health Research and Quality (AHRQ) standards, studies were deemed to be of good quality if they received three or four stars in the selection domain, one or two stars in the comparability domain, and two or three stars in the outcome/exposure domain. Studies were deemed to be of fair quality if they received two stars in the selection domain, one or two stars in the comparability domain, and two or three stars in the outcome/exposure domain. Studies were deemed to be of low quality if they received a score of zero or one in the selection domain, zero star in the comparability domain, or zero or one star in the outcome/exposure domain. Researchers were blind to the study authors when performing the risk of bias assessment. Inter-rater agreement between the two researchers was calculated using non-weighted Kappa statistics and respective 95% confidence interval (CI). A third researcher (A.G.S) was contacted if consensus was not reached.

**Table 3:** Results of risk of bias assessment

Studies	Selection				Comparability		Exposure			Score/Stars	Overall quality
	1	2	3	4	1a	1b	1	2	3		
Bank et al. [38]	*			*	*	*		*		5	Fair
Becerra et al. [41]			*	*	*	*		*		5	Fair
Dietz et al. [65]	*			*	*	*	*			5	Fair
Drummond et al. [63]	*			*				*		3	Low
Eberle et al. [73]	*	*						*		3	Low
Edinger et al. [55]	*		*	*	*	*		*		6	Fair
Enax-Krumova et al. [16]	*	*		*				*		4	Low
Gierthmühlen et al. [15]	*	*		*	*	*		*		6	Fair
Gossrau et al. [72]	*		*	*	*	*		*		6	Fair
Habig et al. [71]	*			*	*	*		*		5	Fair
Huge et al. [47]	*	*		*	*	*		*		6	Fair
Huge et al. [61]	*	*		*	*	*		*		6	Fair
Kemler et al. [44]	*			*				*		3	Low
Knudsen et al. [54]	*			*				*		3	Low
Kolb et al. [61]	*			*	*	*		*		5	Fair
König et al. [39]	*	*		*				*		4	Low
König et al. [40]	*	*		*				*		4	Low
Kumowski et al. [53]	*		*	*	*	*		*		6	Fair
Maier et al. [69]	*			*	*	*		*		5	Fair
Mainka et al. [49]	*	*		*				*		4	Low
Thimineur et al. [57]	*			*	*			*		4	Low
Meyer-Frießem et al. [60]	*			*	*	*		*		5	Fair
Munts et al. [70]	*	*		*	*	*		*		6	Fair
Palmer et al. [52]	*			*	*	*		*		5	Fair
Raj et al. [59]	*			*				*		3	Low
Rasmussen et al. [43]	*			*	*	*		*		5	Fair
Reimer et al. [14]	*			*	*	*		*		5	Fair
Seifert et al. [45]	*			*	*			*		4	Low
Sethna et al. [46]	*		*	*	*	*		*		6	Fair
Sieweke et al. [62]	*			*	*	*		*		5	Fair
Terkelsen et al. [18]	*		*	*	*	*		*	*	7	Good
Truffyn et al. [64]			*	*	*	*		*		5	Fair
Uçeyler et al. [66]	*			*	*	*		*		5	Fair
van Rooijen et al. [50]	*			*	*	*		*		5	Fair
van Rooijen et al. [51]	*			*	*	*		*		5	Fair
Vartiainen et al. [42]	*			*	*			*		4	Low
Vatine et al. [48]	*			*			*	*		4	Low
Weber et al. [58]	*			*	*	*		*		5	Fair
Wittayer et al. [68]	*	*		*	*	*		*		6	Fair
Wolanin et al. [56]	*		*	*				*		4	Low

Selection: (1) the case definition being adequate, (2) representativeness of the cases, 3) selection of controls, (4) definition of controls. Comparability: (1a) study controls of age, (1b) study controls for additional important factor as gender. Exposure: (1) ascertainment of exposure, (2) same method of ascertainment for cases and controls, (3) non-response rate. (*) means the study passed the assessment category

### Data extraction

Data extracted from the included articles were: authors, year of publication, number of participants, diagnostic criteria for CRPS, type, and raw data of measurements (CPT, HPT, PPT, CDT, WDT, TSL, VDT, MDT, MPS, MPT, DMA, WUR, pain area after pinprick hyperalgesia, pain ratings, and CPM), body site where measurements were taken, pain intensity, and details of QST parameters and measurement procedures (including method, number of trials, and devices used) (Table 4). Data extraction was performed by one researcher (M.G.S.) and revised by another researcher (A.G.S.) to confirm the data were correctly gathered. Corresponding authors of the included studies were contacted if there were missing data.

**Table 4:** Results summary of the eligible articles

Authors	Participants	Definition and characteristics of CRPS	Stimulus	Measurement location	Results
Terkelsen et al. [18]	CRPS type 1 (*n* = 18) + CRPS type 2 (*n* = 2) 20 (11 women) with unilateral upper limb CRPS with mean age 45 (ranged from18-72 years) and pain duration of 37 months Healthy Control group: (*n* = 20) matched for age, sex, and BMI Mean age: 44 (ranged from 19 to 68 years)	The diagnosis was based on IASP criteria	PPT, CDT, WDT, HPT, CPT, capsaicin induced pain, flare area, and perfusion area	PPT measured at proximal inter-phalangeal joint of middle finger and skin fold between thumb and index fingers CDT, CPT, WDT, and HPT measured at dorsum of the hand between first and second metacarpal and at thenar eminence using thermal tester (Somedic AB) Capsaicin 5% was applied for 30 min to the dorsum of the hand at fixed skin temperature of 35 °C while laser Doppler perfusion was used to assess the flare area. Also, pain intensity was measured after the application of capsaicin. This variable measured only in 16 patients and 15 healthy subjects	Maximum pain after capsaicin application: In affected and non-affected limbs of CRPS, it was 63 (27/100) and 54 (18) In the control group, it was 37 (27/100) and 36 (22) in the hands matched to the affected and non-affected limbs to CRPS Flare area In affected and non-affected limbs, it was 24.7 (8.3 cm^2^) and 23.5 (11.0 cm^2^) in CRPS In control group was 30.6 (11.5 cm^2^) and 31.1 (10.4 cm^2^) in the matched hands to affected and non-affected limbs to CRPS Area under curve of pain (AUC) In affected and non-affected limbs of CRPS, it was 64.079 (37.99 cm^2^) and 54.354 (27.755 cm^2^) In the control group, it was 33.605 (24.888 cm^2^) and 35.434 (34.108 cm^2^) in the hands matched to the affected and non-affected limbs to CRPS PPT at skin fold: In CRPS group, it was 190 (132 kPa) and 362 (126 kPa) in affected and non-affected hands, respectively In the control group, it was 591 (275 kPa) and 600 (270 kPa) in matched hands to CRPS PPT at inter-phalangeal joint: In CRPS group, it was 172 (98 kPa) and 315 (106 kPa) in affected and non-affected hands, respectively In control group, it was 569 (154 kPa) and 597 (156 kPa) in the matched hand of affected and non-affected hands of CRPS CPT at thenar area: In CRPS group was 23.2 (5.7 °C), 16.6 (6.3 °C) in affected and non-affected hand, respectively In control group was 12.1 (3.0 °C), 12.3 (3.6 °C) in the matched hand to affected and non-affected hands of CRPS group CPT at dorsum of the hand between first and second metacarpal: In CRPS group was 23.4 (5.5 °C), 17.7 (6.7 °C) in affected and non-affected hand, respectively In the control group, it was 11.3 (2.5 °C), 12.3 (4.7 °C) in the matched hand to affected and non-affected hands of CRPS group HPT at thenar area: In CRPS group was 38.1 (4.5 °C), 41.2 (4.2 °C) in affected and non-affected hand, respectively In control group was 45.1 (3.1 °C), 45.2 (3.3 °C) in the matched hand to affected and non-affected hands of CRPS group HPT at dorsum of the hand between first and second metacarpal: In CRPS group was 38.4 (4.1 °C), 41.3 (4.1 °C) in affected and non-affected hand, respectively In control group was 44.3 (3.3 °C), 44.0 (3.6 °C) in the matched hand to affected and non-affected hands of CRPS group
Vartiainen et al. [42]	Chronic upper limb CRPS type 1 (*n* = 8) (All right-handed females; ages 26–57, mean 45.5 years) Mean duration of 5.5 years Healthy age-matched female (*n* = 9) (8 right-handed; ages ranged from 28 to 57, mean 46.0 years)	The diagnosis of CRPS type 1 was based on the criteria of IASP	Presence of allodynia using brush The needed laser intensity to elicit pain and max Pain intensity after laser application. Thulium-laser stimulator (BLM 1000 Tm:YAG; Baasel Lasertech, Starnberg, Germany)	Allodynia was investigated on the affected hand Laser noxious stimulus was applied on the dorsum of the hand	Brush mechanical allodynia was present in all CRPS patients with no identified allodynia in the control group Laser intensity needed to elicit pain in CRPS was 520 mJ while in control group, it was 740 mJ Maximum pain after laser noxious stimulation: In CRPS, it was 7.5 ± 0.7 and 5.4 ± 1.0 in affected and non-affected hands, respectively, while In the control group, it was 4.1 ± 0.5 and 4.1 ± 0.3 in the matched hands
Rasmussen et al. [43]	CRPS type 1 (*n* = 8) (Seven females, one male, mean age 45.5(SD 5.19 years), six upper limbs, two lower limbs) Eight healthy controls (*n* = 8) matched to the Patients by gender and age (seven females, one male, mean age 46.9 (SD 4.83 years) and by handedness	Diagnosis of CRPS type 1 based on Budapest criteria	CDT, CPT, WDT, and HPT were assessed using Medoc TSA-II thermode (Ramat Yishai, Israel) Pain rating after noxious heat or cold was assessed using VAS Presence of allodynia in the affected and contralateral regions was assessed using brush	All variables were measured at the affected limbs and unaffected limbs in CRPS while in the control group the region matched to affected limb was assessed	HPT: In the affected limb of CRPS, it was 35.6 (3.8 °C) while in the unaffected limb, it was 37.58 (1.64 °C) In the control group, it was 38.4 (2.8 °C) NB. There is significant reduction in HPT in CRPS-affected limb than in control (*p* < 0.05) WDT: In the affected limb of CRPS, it was 36 (1.7 °C), while in unaffected limb it was 34.95 (1.61 °C) In the control group, it was 37 (3.4 °C) Data were transformed using Wan’s method from median (maximum, minimum values) CDT: In the affected limb of CRPS, it was -2.43 (2.06 °C), while in unaffected limb it was –3.45 (3.8 °C) In the matched limb of control group, it was − 1.95 (1.05 °C) Data were transformed using Wan’s method from median (maximum, minimum values) CPT: In the affected limb of CRPS, it was − 5.03 (2.75 °C), while in unaffected limb, it was -8.3 (5.5 °C) and In the matched limb of control group was -18(9.8 °C) Data were transformed using Wan’s method from median (maximum, minimum values) Pain rating after noxious cold application (cold pain rating): In affected limb of CRPS, it was 7.0 (7.1), while in unaffected limb it was 1.5 (2.1) In the matched limb of control group, it was 0.0 (0.8) Pain rating after cold application (cold rating): In the affected limb of CRPS was 7.0 (4.0) while in the unaffected limb was 5.04 (2.13) and In the matched limb of control group, it was 2.5 (2.8) Pain rating after noxious heat application (heat pain rating): In affected limb of CRPS, it was 4.25 (3.5), while in unaffected limb it was 1 (1.4) In the matched limb of control group, it was 0.25 (0.35) Pain rating after heat application (heat rating): In the affected limb of CRPS, it was 7.0 (4.3) and in the unaffected limb, it was 5.5 (1.8) In the matched limb of control group, it was 2.8 (2) Data were transformed using Wan’s method from median (maximum, minimum values) Intraepidermal nerve fiber density (IENFD): In the affected limb of CRPS, it was 3.55 (2.39 /mm), while in unaffected limb, it was 2.80 (4.02/mm) and In the control group, it was 14.50 (4.06/mm) Mechanical allodynia was present in all eight subjects with CRPS
Kemler et al. [44]	CRPS type 1 (*n* = 53) (37 women and 16 men), with a mean age of 38.6 years (33 CRPS in the arm, 20 CRPS in the leg). The mean duration of CRPS type 1 was 38.2 months Healthy subjects (*n*=50). (25 females, 25 males) in the control group with age range (20–70 years).	Diagnosis of CRPS type 1 based on IASP criteria	CDT, WDT, CPT, HPT, PPT, and PDT Thresholds for warmth, cold, heat-induced pain, and cold-induced pain were measured using a 5 3 2.5-cm water-cooled Peltier probe (TSA-2001; Medoc Ltd., Ramat Yishai, Israel) PPT was assessed by Semmes–Weinstein monofilaments	Thresholds for the foot was assessed at the dorsal aspects of both feet, immediately proximal to the basis of the second and third toe Thresholds for the hand were assessed at the volar aspects of both wrists, immediately proximal to the base of the hand	In RT side wrist of healthy subjects: CDT was 31.3 (1.4 °C), WDT was 32.5 (2.89 °C), CPT was 5.4 (27.78 °C), HPT was 46.5 (13.7 °C), PDT was 0.07 (0.27 gm) and PPT was not detected through monofilaments used In RT side foot of healthy subjects: CDT was 30.9 (5.1 °C), WDT was 35.4 (20.2 °C), CPT was 3.4 (27.24 °C), HPT was 46.7 (12.45 °C), PDT was 0.34 (1.56 gm) and PPT was not detected through the monofilaments used In affected wrist of CRPS subjects: CDT was 30.9 (4.1 °C), WDT was 33.1 (5.7 °C), CPT was 21 (46.5 °C), HPT was 40.3 (24.18 °C), PDT was 7.9 (255 gm) and PPT 113 (1008gm) In unaffected wrist of CRPS subjects: CDT was 31.3 (1.7 °C), WDT was 32.7 (2.9 °C), CPT was 10.6 (38.24 °C), HPT was 44.6 (16.85 °C), PDT was 0.14 (0.53 gm) and PPT was not detected In affected foot of CRPS subjects: CDT was 28.8 (21.8 °C), WDT was 36.5 (19.00 °C), CPT was 20.1 (49.2 °C), HPT was 41.6 (19.39 °C), PDT was 4.5 (33.8 gm) and PPT 137 (732.4 gm) In unaffected foot of CRPS subjects: CDT was 30 (12.2 °C), WDT was 35.7 (17.3 °C), CPT was 10.5 (39.13 °C), HPT was 44.1 (12.21 °C), PDT was 0.98 (3.87 gm) and PPT could not be detected Mechanical allodynia was present in 27/33 subjects with CRPS in arm while in CRPS of foot, it was 18/20
Seifert et al. [45]	Upper limb CRPS type 1 (*n* = 24) + upper limb CRPS type 2 (*n* = 3) (9 males, 18 females, mean age 57.6 years (2.56) The mean CRPS-duration was 22.12 (SD 4.3 months) Healthy age-matched controls (*n* = 14) (3 males, 11 females, mean age 52.8 years (SD 3.43 years)	The patients had to meet Budapest criteria	Electric detection threshold (EDT), electric pain threshold (EPT), MDT, and MPT Flare area after current stimulation was assessed using laser Doppler perfusion Pinprick hyperalgesic area induced by electric stimulation Normalized electric current needed to induce pain intensity of 6/10 on numerical rating scale Pain rating during adaptation phase to induced electric current	All variables measured in the dorsum of affected hand, contralateral hand, and control group hand of CRPS	In the affected hand of CRPS group, EDT was 1.62 (0.67), EPT was 4.31(2.44), MDT was 28.04 (30.97mN), and MPT was 213.60 (223.1 mN) In the unaffected hand of CRPS group, EDT was 1.32 (0.21), EPT was 4.16(1.92), MDT was 15.58 (16.06 mN), and MPT was 278.11 (247.7 mN) In the control group, EDT was 1.48 (0.3), EPT was 3.80 (1.05), MDT was 8.14 (5.95mN), and MPT was 310.82 (167.25 mN) Data of EDT, EPT, MDT, and MPT was represented as mean (SEM) while SEM was transformed into SD using Cochrane handbook Flare area: In CRPS affected hand, it was 2.2 (0.4 cm^2^), while in unaffected hand it was 2.6 (0.5 cm^2^) and In the control group it was 2.1 (0.4 cm^2^) Area of electrically induced pinprick hyperalgesia: In the affected hand of CRPS, was 12.72 (1.36 cm^2^), while in the unaffected hand was 7.80 (1.44 cm^2^) In the control group, it was 8.03 (1.18 cm^2^) NB. There was a significant increase in hyperalgesic area in affected hands of CRPS subjects compared to control and unaffected hands Normalized electric current needed to induce pain of 6/10 intensity: In the affected hand of CRPS, it was 1.044(0.31au), while in unaffected hand, it was 1.022 (0.26au) In the control group the normalized current was 1.16 (0.42au) The pain rating during electric current adaptation: In affected hand of CRPS, it was 4.15 (2.03), while in unaffected hand, it was 3.92 (1.92) and In the control group was 2.84 (1.39) NB. Enhanced pain facilitation was evident by significant reduction of pain adaptation to electric stimuli in CRPS compared with control. Also, this was confirmed by increased hyperalgesic area after electric stimulation DMA was present in 21 of the patients
Sethna et al. [46] (children and adolescents)	Children and adolescents with CRPS (*n* = 42) (40 females) with mean age of 13.2 (2.6 years) Age and sex matched healthy control (*n* = 101) (53 females) with mean age of 11.5 (3 years)	Both types of CRPS were included based on IASP criteria	WDT, CDT, HPT, CPT, and VDT were assessed through Medoc Ltd., Ramat Yishai, Israel) Static and dynamic allodynia, and allodynia to punctate temporal summation were assessed through brush and pinprick	Assessment of all variables occurred in the most painful area of the affected foot	WDT: In the affected foot of CRPS, it was 35.4 (1.6 °C), while in unaffected foot it was 35 (1.3 °C) In the control group, WDT was 35.3(1.2 °C) CDT: In the affected foot of CRPS group, it was 29.3 (2 °C) while in the unaffected foot, it was 30.3 (1.1 °C) In the control group, CDT was 30 (1.2 °C) HPT In the affected foot of CRPS, it was 39.4(3.15 °C) while in the unaffected foot, it was 40.87 (3.3 °C) In the control group, HPT was 42.6 (3.15 °C) CPT In the affected foot of CRPS, it was 25.53 (6.22 °C), while in unaffected foot it was 18.16 (9.2 °C) In the control group, CPT was 19.4 (9.32 °C) VDT In the affected foot of CRPS group, it was 0.65(0.84 µm) while in the unaffected foot, it was 0.68 (0.48 µm) In the control group, VDT was 0.52(0.21 µm) NB. Data reported as median (1st, 3rd quartiles) and transformed based on Wan’s method Mechanical static allodynia was observed in 29 patients Mechanical dynamic allodynia was observed in 28 patients Allodynia to punctate temporal summation was observed in 30 patients All types of allodynia were observed in 26 patients
Huge et al. [47]	Upper extremity CRPS type 1 (*n* = 61) (54 females) with mean age of 59.1 (12.9 years) The cohort of CRPS was divided into two groups, the first group was acute CRPS (27 patients) with mean disease duration of 3.5 (SD 2.1 months) while the second group was chronic CRPS (34 patients) with mean disease duration of 37.4 (SD 15.1 months) We included the data of chronic cases only Healthy control (*n* = 56) (40 females), with mean age of 56.8 (12.3 years)	CRPS type 1 diagnosed based on the research diagnosis criteria proposed by Bruehl et al. 1999 and IASP diagnostic criteria 1994	CDT, WDT, CPT, HPT, TSL, and paradoxical heat sensation (PHS) were assessed via Medoc Thermal Stimulus Analyser TSA-2001 device (Medoc, Ramat Yishai, Israel)	All parameters were measured at painful area of the affected hand and the matched area of control group (both dominant and non-dominant hands were reported in the study, while we mentioned only the results of dominant hands)	WDT: In the affected hand of CRPS, it was 37.2 (4.1 °C) while in the unaffected hand, it was 36.2(3.00 °C) In the dominant hand of the control group, it was 34.3 (1.5 °C) CDT: In the affected hand of CRPS, it was 28.4 (2.4 °C) while in the unaffected hand, it was 29.2(1.6 °C) In the dominant hand of control group, it was 30.5 (1.00 °C) TSL: In the affected hand of CRPS, it was 0.9 (0.3 °C) while in the unaffected hand, it was 0.8(0.2 °C) In the dominant hand of control group, it was 0.52 (0.3 °C) HPT: In the affected hand of chronic CRPS was 44.21 (4.3 °C) while in the unaffected hand of CRPS, it was 44.6 (4.3 °C) In the dominant hand of control group, it was 45.24 (3 °C) CPT: In the affected hand of CRPS, it was 15.8 (9.9 °C) while in the unaffected hand, it was 13.8 (9.3 °C) In the dominant hand of control group, it was 9.8 (7.6 °C) PHS: In CRPS, it was present in 2/31 patients In the control group, it was almost absent (occurred in 1/336 TSL trials in both hands of healthy controls (0.3%) Results of huge et al. reported as mean (SE) in the article, while we mentioned in the table mean (SD) after using Cochrane guidelines to transform SE into SD
Vatine et al. [48]	CRPS type 1 (*n* = 17) (6 women with mean age of 56.7 (SD 14.5 years) and 11 men with mean age of 37.3 (SD 14.5 years) Symptom duration was 21.1(SD 39.2 months). Only 2 patients have a duration of one month that is not considered chronic 13 patients with other chronic pain conditions (6 women with mean age of 46.2 (26.6 years) and 7 men with mean age of 40 (SD 17.8 years) Pain-free volunteers (*n* = 24) (10 women with mean age of 36.9 (SD 12.7 years) and 14 men with mean age of 40 (SD 11.2 years)	Diagnosis based on the diagnostic criteria of the IASP	PPT Pressure pain tolerance (the pressure that induces intolerable pain)	Sternum	Mean threshold and tolerance values were significantly lower in patients with CRPS (2.7 ± 1.0 kg and 5.4 ± 2.0 kg, respectively) and in patients suffering from other chronic pain syndromes (2.6 ± 1.1 and 4.6 ± 1.7 kg) than in healthy subjects (5.4 ± 2.3 and 8.4 ± 2.6 kg)
Mainka et al. [49]	Upper limb CRPS type 1 (*n* = 18) (7 females) and mean age of 51.7 (10.1 years) The duration of symptoms was 3.3 (SD 2.6 months) 16 subjects with upper limb pain but not diagnosed as CRPS (non-CRPS) with mean age of 45.5 (SD 15.3 years) Healthy control group (*n* = 18) (9 females) with mean age of 41.2 (SD 11.3 years)	Patients with CRPS type 1, diagnosed in accordance with the revised Budapest criteria	PPT was measured using Somedic Production AB, Sweden, algometer type II	Thenar area, metacarpophalangeal (MCP) and proximal inter-phalangeal (PIP) joints. The average PPT of all five MCP and PIP joints was reported	PPT of affected thenar area: In CRPS, it was 243.1 ± 150 kPa while in the unaffected thenar area, it was 522.5 ± 121.9 kPa In the control group, it was 477.9 ± 105.9 kPa PPT of affected MCP joints: In CRPS, it was 79.8 ± 66.6 kPa while in the unaffected thenar area, it was 279.4 ± 148.8 kPa In the control group, it was 254.0 ± 50.4 kPa PPT of affected PIP joints: In CRPS. it was 79.7 ± 55.7 kPa while in the unaffected thenar area, it was 300.2 ± 140.5 kPa In the control group, it was 274.7 ± 75.9 kPa NB. There was a significant reduction of all PPT thresholds in affected hands of CRPS compared to control group The study reported the finding of right and left hands of the control group. Thus, we used the dominant right-hand findings for comparison
Rooijen et al. [50]	Upper limb CRPS type 1 (*n* = 48) (With dystonia, *n* = 31 and mean age of 45.5 (SD 12.4 years); without dystonia, *n* = 17 and mean age of 47.9 (SD 11.7 years) Age and sex matched healthy controls (*n* = 42) (16 women) with mean age of 46.7 (12.0 years)	Diagnosis of CRPS type 1 was based on IASP criteria	CDT, WDT, CPT, HPT, PPT, WUR, and VDT CDT, WDT, CPT, And HPT Measured By TSA-II Neurosensory Analyzer (Medoc Ltd, Ramat Yishai, Israel) VDT was measured via Vibrameter (Type II; Somedic, Stockholm, Sweden) PPT was measured via electronic algometer (FPX50; Wagner Instruments, Greenwich, CT) -WUR was measured via a custom-made pinprick of 256 mN was used	CDT, WDT, CPT, HPT, and WUR Measured at dorsum of the hand PPT was measured over the M. abductor pollicis brevis VDT was measured over the first metacarpal bone	In affected hand of CRPS group with dystonia: CDT was 29.4 (28.0–30.9 °C), WDT was 36.4 (34.7–43.5 °C), CPT was 26.5 (4.8–30.0 °C), HPT was 41.7 (35.6–48.0 °C), WUR was 1.4 (1–2), PPT was 2.0 (1.0–3.8 kg/cm), and VDT was .45 (.40–5.5 µm) In affected hand of CRPS group without dystonia: CDT was 30.0 (28.4–30.6 °C), WDT was 36.4 (35.3–43.2 °C), CPT was 24.5 (12–26.7 °C), HPT was 44.7 (38.9–48.1 °C), WUR was 1.4 (1–3.4), PPT was 3.4 (2.2–5.6 kg/cm), and VDT was .35 (.20–1.05 µm) In matched hand of control group without dystonia: CDT was 30.9 (30.1–31.3 °C), WDT was 35.2 (33.4–36.1 °C), CPT was 13.8 (4.9–22.5 °C), HPT was 43.7 (40.8–47.6 °C), WUR was 2.0 (1–3), PPT was 6.1 (4.9–6.9 kg/cm), and VDT was .22 (.17–.41 µm) All data reported as median and IQR while transformed into mean and SD using Wan’s method In both types of CRPS, there was significant loss of warm, cold, vibration detection thresholds as well as significant gain of pressure pain and cold pain thresholds compared to control group NB. compared to healthy controls, pain levels after the first pinprick were significantly higher in CRPS patients with dystonia (*p* = .001)
Rooijen et al. [51]	Upper limb CRPS type 1 (*n* = 48) (With dystonia, *n* = 31 and mean age of 45.5 (12.4 years); without dystonia, *n* = 17 and mean age of 47.9 (11.7 years) Age and sex matched healthy controls (*n* = 42) (16 women) with mean age of 46.7 (12.0 years)	Diagnosis was based on IASP criteria	CDT, WDT, CPT, HPT, PPT, WUR, and VDT	CDT, WDT, CPT, HPT, and WUR Measured at matched site in contralateral arm (21 measurements), contralateral leg (30 measurements), cheek (48 measurements), and ipsilateral site away from the affected area (24 measurements)	WDT measured at contralateral arm of CRPS was 34.9 (34–38.2) while in controls, WDT was 34.5 (33.6–36.1) In the ipsilateral leg of CRPS, WDT was 37.6 (35.5–42.4) while in healthy group, WDT was 37.3 (35.6–40) In the contralateral leg, WDT in CRPS was 38.3 (35.7–41.7) while in control group, it was 37 (34.9–39) In cheek, WDT in CRPS was 35.1 (34.3–36.5), while in the control group, it was 34.3 (33.4–35.3) CDT of the contralateral arm of CRPS was 30.3 (29.6–31.3), while in control group, it was 30.6 (30.2–31.1) CDT of the ipsilateral leg of CRPS was 27.5 (24.5–29.4), while in control group, it was 29.4 (27–30.3) CDT of the contralateral leg of CRPS was 29.1 (27.2–30.3), while in the control group, it was 28.2 (26.6–29.7) CDT of the cheek of CRPS was 30.7 (30.3–31.0), while in the control group, it was 31 (30.4–31.4) HPT of the contralateral arm of CRPS was 42.7 (4.7), while in the control group, it was 44.3 (3.9) HPT of the ipsilateral leg of CRPS was 44.5 (4.1), while in the control group, it was 45.4 (3) HPT of the contralateral leg of CRPS was 43.6 (4), while in the control group, it was 45.5 (2.8) HPT of the contralateral arm of CRPS was 39.3 (37.3–46.4), while in the control group, it was 45.1 (41–47.6) CPT of the contralateral arm of CRPS was 22.9 (6.1–26.4), while in the control group, it was 9.3 (3.1–20.8) CPT of the ipsilateral leg of CRPS was 16.8 (0–24.9), while in the control group, it was 10.2 (0–19.9) CPT of the contralateral leg of CRPS was 20.2 (8.1–24.6), while in the control group, it was 6.5 (0–20.9) CPT of the cheek of CRPS was 26.2 (4.4–28.4), while in the control group, it was 16.7 (0.6–23.5) PPT of the contralateral arm of CRPS was 3.6 (2.7–5.4), while in the control group, it was 6.2 (4.9–7.5) PPT of the ipsilateral leg of CRPS was 4.6 (3.4–7.1), while in the control group, it was 6.9 (5.7–8.6) PPT of the contralateral leg of CRPS was 4.6 (3.6–6), while in the control group, it was 6.6 (5.6–8) PPT of the cheek of CRPS was 1.4 (1–2), while in the control group, it was 2 (1.7–2.5) VDT of the contralateral arm of CRPS was 0.32 (0.23–1.13), while in the control group, it was 0.31 (0.22–0.38) VDT of the ipsilateral leg of CRPS was 2.8 (1.4–11.5), while in the control group, it was 2.0 (0.68–7.6) VDT of the contralateral leg of CRPS was 2.2 (1.0–11.2), while in the control group, it was 1.9 (0.64–6.8) VDT of the cheek of CRPS was 1.6 (0.84–2.39), while in the control group, it was 0.8 (0.41–1.89) All data reported as median and IQR while transformed into mean and SD using Wan’s method
Palmer et al. [52]	Unilateral upper or lower CRPS type 1 (*n* = 36) (29 female) Mean age was 48.94 ± 13.70 years) Healthy controls (*n* = 37) (29 women) with mean age of 50.27 (15.28 years)	Based on Budapest criteria for unilateral CRPS for upper and lower limb affection	Cold and heat sensitivity based on numerical rating from 0 to 10 using Hot and cold metal rollers (Therroll; Somedic Production AB, Sweden) PPT was assessed on distal phalanges of index fingers bilaterally using digital algometer (Somedic Production AB) Light touch threshold (LTT) was measured using Von Frey monofilaments on hands and sternum	Cold and hot sensitivity was measured using a scale from 0 to 10, at the nearest location to the most painful area at which patients can tolerate heat and cold sensations PPT was measured at distal phalanges of index fingers LTT was measured at both hands and sternum	In the affected region of CRPS, cold sensitivity was 3.00 (1.00) while heat sensitivity was 6.00 (1.00), LTT of the affected hand was 0.14 (0.69gm), LTT of sternum was 0.09 (0.70gm), and PPT of the affected index finger was 182 (102 kPa) In the unaffected region of CRPS, cold sensitivity was 3.00 (0.13) while heat sensitivity was 6.00 (1.00), LTT of the unaffected hand 0.12 (0.31gm), and PPT of the unaffected index finger was 225 (169 kPa) In the dominant right side of control group, cold sensitivity was 3.00 (1.00) while heat sensitivity was 6.00 (0.00), LTT of the right hand was 0.06 (0.09 gm), LTT of the sternum was 0.05 (0.11gm), and PPT of right index finger was 280 (144 kPa) Data were represented as median and IQR, while we used these values as a mean and SD during meta-analysis as we could not transform these values Both results of right and left sides of control group were reported, while we mentioned only the right dominant side
Kumowski et al. [53]	CRPS type 1 (*n* = 21) + CRPS type 2 (*n* = 3) Mean age was 51.6 (SD 9.8 years) and the disease duration was 24.2 (SD 11.7 weeks) Age and gender matched healthy controls (*n* = 23)	Based on the Budapest research criteria for CRPS	CPM	A heat pain stimulus served as test stimulus (TS) and was applied to a pain-free area on the volar forearm of the affected side with a 30 by 30 mm2 contact stimulation device (TSA-II, Medoc, Ramat Yishai, Israel) Conditioned stimulus of cold-water immersion applied on contralateral hand All QST parameters were measured at the thenar area bilaterally	In CRPS, heat pain (TS) before conditioning was 46.2 (13.2), during conditioning was 31.4 (15.7), five minutes after conditioning was 40 (14.6) Regarding conditioned pain modulation (CPM) effect: Early CPM (TS during—before) was − 14.7 (15.7), while late CPM (TS after 5 min—before) was − 6.2 (9.4) In the control group, heat pain (TS) before conditioning was 50.2 (12.3), during conditioning was 37.7 (15.8), five minutes after conditioning was 44.6 (15.9) Regarding conditioned pain modulation (CPM) effect, Early CPM (TS during—before) was − 12.5 (12.4), while late CPM (TS after 5 min—before) was − 5.6 (13) Data of heat pain and CPM effect represented as mean and (SE) Based on QST, somatosensory abnormalities in the CRPS group were loss of thermal detections (CDT: 25%, WDT: 21%; TSL: 33%), thermal hyperalgesia (CPT: 17%, HPT: 22%), and mechanical hyperalgesia (MPT: 33%) QST values were represented as z-score. A value outside the 95%-confidence interval of published data from healthy subjects (corresponding to z-values higher than + 1.96 or lower than -1.96) were considered as abnormal value (Rolke et al. 2006; Magerl et al. 2010)
Gierthmühlen et al. [15] (frequencies of sensory abnormalities)	CRPS type 1 (*n* = 298) (233 females) Mean age was 53.0 (SD 13.4 years) and symptoms duration was 21.2 (SD 35.5 months) CRPS type 2 (*n* = 48) (38 females) The mean age was 52.3 (SD 12.0 years) and symptoms duration was 25.1 (SD 33.3 months) Healthy controls (*n* = 180) (110 females) Mean age of 38.4 (SD 12.9 years)	Based on IASP and Budapest criteria	CDT, WDT, TSL, CPT, HPT, MDT, MPT, MPS, WUR, VDT, PHS, MDA (mechanical dynamic allodynia), and PPT Thermal thresholds measured by using a TSA-2001-II (MEDOC, Ramat Yishai, Israel) MDT was assessed using a standardized set of modified von Frey hairs (Optihair2-Set, Marstock Nervtest, Schreisheim, Germany) exerting forces between 0.25 and 512 mN MPT, WUR, and MPS were assessed using custom-made weighted pinprick stimuli (the PinPrick; MRC Systems GmbH, Heidelberg, Germany) Gentle/light stroking with a cotton wisp (around 3 mN), a cotton wool tip fixed to an elastic strip (around 100 mN), and a brush (around 200–400 mN) used to assess DMA VDT was measured via Rydel–Seifert et al. tuning fork (64 Hz, 8/8 scale) Pressure gauge device (FDN200, Wagner Instruments, Greenwich, CT, USA) used to assess PPT	The most painful area of the hand in CRPS was assessed while dorsum of the hand in the control group was assessed	In the affected limb of CRPS group, the sensory gain of CDT was 3.8%, WDT was 2.2%, TSL was 2.65%, CPT was 30%, HPT was 39.7%, MDT was 7.85%, MPT was 32.9%, MPS was 42.1%, WUR was 12%, VDT was 1.7%, PPT was 70%, and DMA was 26.1% In the healthy controls, the sensory gain of CDT was 2.3%, WDT was 6.1%, TSL was 5.00%, CPT was 4.5%, HPT was 3.9%, MDT was 6.2%, MPT was 3.3%, MPS was 5%, WUR was 7.2%, VDT was 6.7%, PPT was 5.6%, and DMA was 1.1% In the affected limb of CRPS group, the sensory loss of CDT was 36.3%, WDT was 28%, TSL was 26.15%, CPT was 6.2%, HPT was 8.8%, MDT was 42.7%, MPT was 11%, MPS was 8.9%, WUR was 2.3%, VDT was 36.9%, and PPT was 4% In the healthy controls, the sensory loss of CDT was 6.7%, WDT was 7.2%, TSL was 5.5%, CPT was 4.4%, HPT was 2.8%, MDT was 6.1%, MPT was 5%, MPS was 2.2%, WUR was 2.8%, VDT was 1.1%and PPT was 3.4%
Knudsen et al. [54]	Upper limb CRPS type 1 (*n* = 17) + lower limb CRPS type 1 (*n* = 17) Healthy controls (*n* = 45) (14 male). Age ranged from 17 to 51 years	Based on IASP research diagnostic criteria	PPT at forehead bilaterally. Pressure was applied using a spring loaded algometer with a rounded tip (1 cm in diameter) Sharpness to mechanical stimulus was assessed based on a scale from 0 (not sharp) to 10 (stabbing) in response to a single application with a firm nylon bristle (Filament 17, Senselab von Frey Aesthesiometer, Somedic Sales AB, Sweden) 22 patients underwent cold pressor pain modulation by immersion of affected and contralateral sides in cold water 2 °C if patients cannot withstand because of allodynia, cold water of 10 °C was used	Bilateral forehead ipsilateral to affected side and contralateral to it	In CRPS patients, PPT at ipsilateral and contralateral forehead was 497 (48 g) versus 648 (43 g), respectively In CRPS, sharpness to punctate stimulation on the ipsilateral than contralateral side of the forehead in both groups of patients (mean rating 3.6 (.3) versus 2.6 (.2)), respectively In the control group, PPT of right-side forehead = 675.71 (30 g) while for left forehead, it was 652.00 (33 g) Sharpness of the control group at right-side forehead was 3.2 (0.2) while at left side, it was 3.3 (0.3) For conditioned pain modulation: Forehead asymmetry increased after immersion of the CRPS-affected limb (from 556.5 (61 gm) to 418.5(53.7gm) in ipsilateral side and from 606.12 (72.5 gm) to 509.8 (53.5 gm) in contralateral side; significant reduction after the affected limb immersion, *p* < .05), but not after immersion of the healthy limb, from 500 (55 gm) to 439 (55 gm) in ipsilateral side and from 575.5 (49 gm) to 550 (57 gm) in contralateral limb after 2 min immersion Sharpness was symmetrical in the forehead during each limb immersion and did not change after the immersions In the control group, sharpness also did not change while the PPT diminished significantly after cold pressor (from 675 (30 gm) to 564 (22gm) in ipsilateral side and from 652 (33 gm) to 572 (28 gm) in contralateral side Sharpness did not significantly change after cold pressor application NB. This indicates disturbances of inhibitory control in CRPS Data represented as mean and SE, while we used Cochrane guidelines to transform data into mean (SD) NB. Results of CRPS and healthy controls were published separately while the same research group introduced both studies. Also, the study of CRPS compared PPT with the control values published before CRPS study
Edinger et al. [55]	CRPS (*n* = 20) accompanied with total body pain The average duration was 109.6 (SD 63.4 months). In CRPS, there were 18 women (age 21–59 years; average age, 39.9 years) and 2 men (ages 24 and 46; average age, 35 years) Age and gender matched healthy controls (*n* = 10)	Based on the Budapest clinical criteria	static and mechanical allodynia, thermal allodynia, mechanical hyperalgesia, and after sensations Static non hyperalgesia allodynia was measured using a Wagner Force Dial TM algometer (Wagner Instruments, Greenwich, CT) DMA measurements were obtained using a standard one-inch foam brush A metal tuning fork chilled in an ice water bath to 2 °C was utilized to evaluate cold thermal allodynia The threshold for algesic mechanical hyperalgesia was measured using a Neuropen (Owen Mumford, Oxford, UK) After sensation, pain perception lasting longer than 30 s after the stimulus withdrawal was recorded following the 4 sensory tests on each limb	Most painful areas of eight selected body regions of (face, chest, abdomen, right arm, left arm, right leg, left leg, and back	All patients with CRPS showed a significantly lower pain threshold for static allodynia in all body regions tested compared to the control participants (face, *p* = 0.045; chest, *p* = 0.004; abdomen, *p* = 0.012; right arm, *p* = 0.001; left arm, *p* < 0.0001; right leg, *p* < 0.0001; left leg, *p* < 0.0001; back, *p* < 0.0001) More than half of the patients with CPRS showed a significantly lower pain threshold for dynamic allodynia in all body regions tested compared to control participants (face, *n* = 12, *P* = 0.0225; chest, *n* = 15, *P* < 0.0001; abdomen, *n* = 13, *P* < 0.0001; right arm, *n* = 15, *P* < 0.0001; left arm, *n* = 16, *p* = 0.001; right leg, *n* = 18, *p* = 0.0001; left leg, *n* = 17, *p* = 0.00064; back, *n* = 13, *p* < 0.0001) At least 85% of the patients with CRPS had a significantly lower pain threshold for mechanical hyperalgesia in all body areas compared to control participants (face, *n* = 17, *p* = 0.001; chest, *n* = 19, *p* = 0.0001; abdomen, *n* = 18, *p* = 0.0001; right arm, *n* = 20, *p* < 0.0001; left arm, *n* = 18, *p* < 0.0001; right leg, *n* = 20, *p* < 0.0001; left leg, *n* = 19, *p* < 0.0001; back, *n* = 19, *p* < 0.0001) For cold allodynia, the median pain rating in all areas for the control participants was 0. For the patients with CRPS, the median pain rating for the face, chest, right upper extremity, and back was 5. For the left upper extremity and right lower extremity the median pain rating was 6. For the abdomen the median pain rating was 3 and for the left lower extremity it was 7 (face, *p* < 0.01; chest, *p* < 0.15; abdomen, *p* = 0.40; right arm, *p* < 0.0001; left arm, *p* < 0.0001; right leg, *p* = 0.013; left leg, *p* = .001; back, *p* = 0.14) There were significantly more reports of after sensation in all 4 limbs of the patients with CRPS compared to control participants following static touch (right arm, *p* = 0.004; left arm, *p* = 0.079; right leg, *p* = 0.0004; left leg, *p* = 0.003). There were also significantly more reports of after sensation following pin prick (right arm, *n* = 20, *p* = 0.001; left arm, *n* = 18, *p* < 0.0001; right leg, *n* = 20, *p* < 0.0001; left leg, *n* = 19, *p* < 0.0001)
Wolanin et al. [56]	CRPS (*n* = 32) (23 women) Mean age was 45.4 years) and duration of symptoms was 9.67 years Age and sex matched healthy controls (*n* = 35) (19 women) The mean age was 42 years	Diagnosis was based on IASP criteria	Static and mechanical allodynia, thermal allodynia, mechanical hyperalgesia, and after sensations The testing of thermal allodynia to cold utilized the metal handle of a standard reflex hammer at room temperature An algometer with a 1 cm^2^ rubber tip FDK 20 (Wagner Insling, Greenwich, CT) was utilized to measure static mechano-allodynia Wind-up pain was elicited by 6 depressions of a von Frey hair at half second intervals: the duration of the elicited pain was measured for 30 s A foam brush (3 inches in diameter) was lightly brushed over the skin at 6 cm/sec Pinprick was utilized to measure a sharp mechanical (algesic) stimulus. One pinprick stimulus (a 2-inch pin steel safety pin with nickel plating) was applied	In the affected limb of CRPS and both at the dominant and non-dominant side of control group	The spread of thermal allodynia in CRPS was 5.47 ± 0.78 cm while in the control group, it was 0 cm. Duration of cold sensation in CRPS was 24.38 ± 2.10 s while in the control group, it was 1.46 ± 0.92 s DMA rated by using NRS in CRPS was 6 (0–10) (median and range) while in the control group, it was 0 Static mechanical allodynia in CRPS was 3.21 ± 0.31 Ibs while in the control group, it was 10.48 ± 0.13 Ibs Hyperalgesia measured through Von Frey Hair in CRPS was 6 (0–10) (median and range) while in the control group, it was 0 (0–2) Wind-up pain in CRPS was 8 (0–10) while in the control group, it was 0 (0–4) Pinprick pain in CRPS was 8 (0–10) (median and range) while in the control group, it was 0 (0–2). Also, Pinprick spread in CRPS was 5.75 ± 0.73 cm while in the control group, it was 0.01 ± 0.01. Finally, Pinprick after sensation in CRPS was 24.22 ± 2.02 s while in the control group, it was 0 s Results were reported as median (range) or mean (SE) and we used Cochrane guidelines to transform data into mean (SD)
Truffyn et al. [64] (children)	Children with lower limb CRPS type 1 (*n* = 34) The mean age was 12.03 (SD 2.4 years) and mean duration was 8.8(SD 11.5 months) Age and sex matched healthy controls (*n* = 56) (28 females) Mean age was 15.7 (SD 1.1 years)	Not mentioned	CDT, WDT, CPT, and HPT Thermal stimulation was accomplished using Medoc Neuro Sensory Analyzer, Model TSA-II (Medoc Ltd, Ramat Yishai, Israel)	Pain site and contralateral site in CRPS, and matched site in the healthy control group	In CRPS-pain site, CDT was 28.5 (2.9 °C), WDT was 37.5 (3.6 °C), CPT was 18.9 (10.3 °C), and HPT was 41.3 (4.1 °C) In CRPS-contralateral site, CDT was 29.1 (2.12 °C), WDT was 36.5 (2.8 °C), CPT was 18.2 (10.2 °C), and HPT was 41.3 (3.7 °C) In the control group, CDT was 30.9 (0.82 °C), WDT was 33.67 (1.02 °C), CPT was 17.89 (10.3 °C), and HPT was 39.95 (4.05 °C)
Raj et al. [59]	CRPS type 1 and 2 (*n* = 36) with age greater than 18 years) Healthy control group: (*n* = 57)	CRPS Type 1 or 2 subject inclusion criteria were adapted from those outlined by Stanton-Hicks et al. (IASP criteria)	Current perception threshold (CPT) and pain tolerance threshold (PTT) to an electric stimulus DMA was assessed also	Affected finger or great toe in CRPS group and matched site in the control group	The difference between the PTT values for the CRPS subjects from the symptomatic site and the asymptomatic control site and the healthy controls was statistically significant (*P* < .05) NB. No data available for the control group DMA was present in 29/36 of CRPS subjects
Weber et al. [58]	CRPS (*n* = 10) The mean age was 44.6 (range 35–56) years. The mean duration of CRPS symptoms was 32 weeks (range 4–190) Age and gender matched healthy controls (*n* = 10) The mean age was 44.8 (range 24–78) years	All patients fulfilled the following IASP diagnostic criteria (Stanton-Hicks et al. [110]):	Axon reflex vasodilation (flare area) after electrical stimulation using laser Doppler imaging	Forearm in upper limb CRPS and leg in lower limb CRPS	In CRPS, axon reflex vasodilation was 438 (68%) after the stimulation In the control group, axon reflex vasodilation was 306 (52%) after the stimulation
Sieweke et al. [62]	CRPS (*n* = 40). This cohort further divided into a group of 23 patients and another group of 17 patients The mean age was 48.6 years Age and sex matched healthy control (*n* = 15) Mean age of 46 years	Based on the current IASP criteria (Stanton-Hicks et al. [110])	HPT, MPT, MPS, and WUR	Affected foot and hand in CRPS while in the control group the sites matched to that of CRPS were assessed	In the group of 23 patients: in the affected site, HPT was 44.2 (4.12 °C). In contralateral site, HPT was 44.2 (2.6 °C) In the group of 17 patients: in the affected site, MPS was 48.5 (40) and WUR was 2.1 (5.8). In contralateral site, MPS was 42.4 (22.9) and WUR was 1.8 (5.2) In the control group: in the dominant site, HPT was 44 (2 °C) while MPS was 46.6 (19) and WUR was 1.9 (4.8)
Reimer et al. [14] (frequencies of sensory abnormalities)	Upper limb CRPS type 1 (*n* = 19) Mean age was 60.2 (SD 12.9 years) and duration of symptoms was 5.7(SD 8.3 months) The reference value of Rolke et al. 2006 was used as a healthy reference (180 subjects)	Diagnosis of CRPS type 1 was based on Budapest criteria for clinical diagnosis	CDT, WDT, TSL, CPT, HPT, MDT, MPT, MPS, WUR, VDT, PPT, PHS, and DMA	QST measurements were taken according to German network guidelines	In the affected limb of CRPS group, the sensory gain of CDT was 0.00%, WDT was 0.00%, TSL was 5.3%, CPT was 36.8%, HPT was 36.8%, MDT was 5.3%), MPT was 5.3%, MPS was 42.1%, WUR was 6.7%, VDT was 5.3%, PPT was 100%, and DMA was 26.3% In the unaffected limb of CRPS group, the sensory gain of CDT was 5.3%, WDT was 0.00%, TSL was 0.00%, CPT was 10.5%, HPT was 0.00%, MDT was 0.00%, MPT was 0.00%, MPS was 10.5%, WUR was 0.00%, VDT was 0.00%, PPT was 15.8%, and DMA was 0.00% In the healthy controls, the sensory gain of CDT was 2.3%, WDT was 6.1%, TSL was 5.00%, CPT was 4.5%, HPT was 3.9%, MDT was 6.2%, MPT was 3.3%, MPS was 5%, WUR was 7.2%, VDT was 6.7%, PPT was 5.6%, and DMA was 1.1% In the affected limb of CRPS group, the sensory loss of CDT was − 31.6%, WDT was 26.3%, TSL was 26.3%, CPT was 10.5%, HPT was 5.3%, MDT was 31.6%, MPT was 5.3%, MPS was 5.3%, WUR was 6.7%, VDT was 42.1%, and PPT was 00% In the unaffected limb of CRPS group, the sensory loss of CDT was 0.00%, WDT was 15.8%, TSL was 0.00%, CPT was 0.00%, HPT was 0.00%, MDT was 5.3%, MPT was 0.00%, MPS was 15.8%, WUR was 6.7%, VDT was 0.00%, and PPT was 15.8% In the healthy controls, the sensory loss of CDT was 6.7%, WDT was 7.2%, TSL was 5.5%, CPT was 4.4%, HPT was 2.8%, MDT was 6.1%, MPT was 5%, MPS was 2.2%, WUR was 2.8%, VDT was 1.1%, and PPT was 3.4%
Thimineur et al. [57]	CRPS (*n* = 140) Healthy controls (*n* = 26)	Patients identified as having CRPS met all six of our clinical criteria: 1. pain of an extremity disproportionate to physical injury 2. hyperalgesia and hyperpathia that were inconsistent with a peripheral nerve or spinal root pattern 3. absence of underlying peripheral pathology that would otherwise explain pain and sensory abnormalities 4. evidence of autonomic dysfunction such as color, temperature, or edematous Changes 5. history of precipitant trauma (including surgery), stroke, or spinal cord injury 6. symptoms present for at least 3 months; there was no upper limit on symptom duration	TSL, PPT, MDT, and pain ratings after suprathreshold ethanol application NB. PPT was measured at hand in case of affected feet and in feet in case of affected hand	TSL was measured at the affected side (foot, hand, ophthalmic area, maxilla) and the contralateral side. Corresponding sites in the control group were also assessed PPT was measured at the dorsum of the hand and the feet MDT was measured at the palmar surface of the hand Oral pain perception to suprathreshold stimulus was assessed at tongue	In CRPS, TSL in the foot of affected side was 18(10.7) while in the control group was 9.5(7.5) In CRPS, TSL in the foot contralateral to the affected side was 13 (9.2) while in the control group was 7 (3) In CRPS, TSL in the hand of affected side was 12.2 (10.1) while in the control group, it was 3.9 (2) In CRPS, TSL in the hand contralateral to the affected side was 8.2 (8) while in the control group, it was 3.7 (2.6) In CRPS, TSL in the maxilla of affected side was 6.5 (7.2) while in the control group, it was 1.8 (1.4) In CRPS, TSL in the maxilla contralateral to the affected side was 4 (5.5) while in the control group, it was 1.7 (1.3) In CRPS, TSL in the ophthalmic area of affected side was 11.8 (9.5) while in the control group, it was 3.2 (2.7) In CRPS, TSL in the ophthalmic area contralateral to the affected side was 5.5 (6.5) while in the control group, it was 3.8 (5.6) In CRPS, pain rating to suprathreshold stimulus in the tongue of the affected side was 163.5 (87) while in the control group, it was 236 (115) In CRPS, pain rating to suprathreshold stimulus in the tongue contralateral to the affected side was 203 (116.5) while in the control group, it was 240 (118) In CRPS, MDT of the affected side was 3.9 (0.9) while in the control group, it was 2.9 (0.3) In CRPS, MDT in the hand contralateral to the affected side was 3.3 (0.75) while in the control group, it was 2.8 (0.3) In CRPS, MDT of the affected side was 3.9 (0.9) while in the control group, it was 2.9 (0.3) In CRPS, MDT in the hand contralateral to the affected side was 3.3 (0.75) while in the control group, it was 2.8 (0.3) In CRPS, PPT of the hand ipsilateral to the affected foot was 11.2 (5.1 pound) while in the control group, it was 8.0 (3.5 pound) In CRPS, PPT of the hand contralateral to affected foot was 8.5 (5 pound) while in the control group, it was 8 (3.3 pound) In CRPS, PPT of the foot ipsilateral to affected hand was 13.4 (5.65 pound) while in the control group, it was 11.2 (5.2 pound) In CRPS, PPT of the foot contralateral to affected hand was 11.6 (5.3 pound) while in the control group, it was 10.5 (3.5 pound)
Drummond et al. [63]	CRPS type 1 (*n* = 24) + CRPS type 2 (*n* = 6) (22 females and 8 males) (18 upper limb and 12 lower limb CRPS) The mean age was 49 (11 years) Healthy controls (*n* = 20) (15 females) The mean age was 46.5 (13.5 years)	Based on Budapest criteria for diagnosis of CRPS	MPS, electric pain sensitivity, and PPT Monofilament (× 5) used to assess MPS, an algometer (FDX, Wagner Instruments, Greenwich, CT) used to assess PPT	Affected extremity, contralateral extremity, forehead	In CRPS, MPS of the affected limbs was 4.1 (0.8) while in the contralateral limbs, it was 2.7 (0.5) In the control group, MPS in the area matched to affected area was 0.6 (0.2) In CRPS, MPS was 2.4 (0.6) in area away from the affected part in ipsilateral limb while in matched area in the contralateral limb, it was 4 (0.4) In the control group, MPS at area matched to remote area of affected region was 0.6 (0.16) In CRPS (forehead area), MPS in the ipsilateral forehead was 2.9 (0.5) and in the contralateral forehead, it was 2.6 (0.4) In the control group (forehead area), MPS was 0.66 (0.1) In CRPS, PPT of the affected limbs was 0.825 (0.275 kg) while in the contralateral limbs, it was 1.5 (0.25 kg) In the control group, PPT in the area matched to the affected area was 2.1 (0.24 kg) In CRPS, PPT was 1.3 (0.25 kg) in area away from the affected part in ipsilateral limb while in matched area in the contralateral limb, it was 1.7 (0.25 kg) In the control group, MPS at area matched to the remote area of affected region was 2 (0.2) In CRPS (forehead area), PPT in ipsilateral forehead was 0.8 (0.1 kg) and in the contralateral forehead, it was 0.9 (0.1 kg) In the control group (forehead area), PPT was 1.2 (0.2 kg) Measurements were reported as mean (SE)
Maier et al. [69] (frequencies of sensory abnormalities)	CRPS (*n* = 403) (312 females) Mean age was 52 (13 years) The reference value of Rolke et al. 2006 was used as a healthy reference (180 subjects)	Based on the revised criteria of Bruehl et al. 1999 and Budapest criteria	CDT, WDT, TSL, PHS, CPT, HPT, MDT, MPT, MPS, WUR, VDT, PPT, and CPM	CDT, WDT, TSL, CPT, HPT, MPT, MDT, MPS and VDT were measured from the middle finger. PPT was measured by pressure algometer at thenar eminence Vibration detection threshold (VDT) was tested with a Rydel–Seifert et al. graded tuning fork (64 Hz, 8/8 scale) To test wind up ratio (WUR), a single stimulus was applied with a 256 mN pinprick stimulator probe. Then, at intervals of ten seconds a series of ten identical pinprick stimuli were applied in the same skin area. Participants were asked to rate the intensity using a numeric rating scale from 0 to 100 immediately after the single stimulation and again after the series of 10 stimuli MPT was tested using blunt probes with increasing pressure intensities MPS was calculated as the geometric mean of the pain ratings for pinprick stimuli MDT was tested using glass von Frey hairs DMA using a cotton wisp, a Q-Tip and a standardized brush (Somedic, Sweden) CPM was measured through investigating the effect of heat noxious stimulus on PPT measured at dorsal forearm before and after the noxious stimulus A calibrated MSA thermal sensory analyzer (Somedic, Sweden) with baseline temperature was 32 °C	Affected limb of CRPS group, the sensory gain of CDT was 2.7%, WDT was 2.5%, TSL was 2.7%, CPT was 30.5%, HPT was 40.1%, MDT was 9.5%, MPT was 28.7%, MPS was 46.6%, WUR was 13.1%, VDT was 1.5%, PPT was 66.3%, and DMA was 24.1% The healthy controls, the sensory gain of CDT was 2.3%, WDT was 6.1%, TSL was 5.00%, CPT was 4.5%, HPT was 3.9%, MDT was 6.2%, MPT was 3.3%, MPS was 5%, WUR was 7.2%, VDT was 6.7%, PPT was 5.6%, and DMA was 1.1% Affected limb of CRPS group, the sensory loss of CDT was 32.5%, WDT was 26.6%, TSL was 26.9%, CPT was 5.2%, HPT was 7.7%, MDT was 35.2%, MPT was 10%, MPS was 6.2%, WUR was 2.7%, VDT was 35.4%, and PPT was 3.3% The healthy controls, the sensory loss of CDT was 6.7%, WDT was 7.2%, TSL was 5.5%, CPT was 4.4%, HPT was 2.8%, MDT was 6.1%, MPT was 5%, MPS was 2.2%, WUR was 2.8%, VDT was 1.1% and PPT was 3.4%
Bank et al. [38]	Upper extremity CRPS type 1 (*n* = 25) Mean age was 50.6 (SD 13.7 years) Age and gender matched healthy controls (*n* = 50) Mean age was 50.1(SD 13.4 years)	Diagnosis based on IASP criteria	PPT and VDT	Affected and unaffected extremities in CRPS group and dominant and non-dominant extremities in healthy group	In the affected extremity of CRPS, PPT was 1.77 (1.07 kg) and VDT was 0.5 (0.36 µm) In the unaffected extremity of CRPS, PPT was 3.6 (1.26 kg) and VDT was 0.43 (0.39 µm) In the dominant side of healthy controls, PPT was 4.3 (1.6 kg) and VDT was 0.49 (0.34 µm)
Kolb et.al [67]	CRPS type 1 (*n* = 17) + CRPS type 2 (*n* = 3) Mean age was 54.2(SD 12.5 years) Age and gender matched healthy controls (*n* = 20) The mean age was 54.6 (SD 13.5 years)	Diagnosis based on Budapest criteria	CDT, WDT, TSL, CPT, HPT, TDT, VDT, WUR, PPT, MPT, and PHS Allodynia was also assessed	Affected and un-affected extremity of CRPS and dominant extremity of control group	In the affected limb of CRPS group, CDT was 26.7 (1.6), WDT was 35.7 (0.9), TSL was 6.8 (2.33), CPT was 15.6 (2.7), HPT was 41.4 (1.6), TDT was 26.6 (22.4), MPT was 138.8 (36.5), MPS was 2.14 (0.52), WUR was 4 (1.06), VDT was 7.59 (1.83), PPT was 291.2 (73.4), and DMA was 5 (1.35) In the healthy controls, CDT was 29.5 (0.32), WDT was 35.5 (0.57), TSL was 3.94 (0.55), CPT was 11.9 (2.1), HPT was 43.8 (0.86), TDT was 4.16 (2.72), MPT was 148.5 (41.3), MPS was 1.3 (0.4), WUR was 2 (0.21), VDT was 7.6 (0.12), PPT was 814.65(83.68), and DMA was 0.01 (0.01) In the unaffected limb of CRPS group, CDT was 29.5 (0.39), WDT was 34.8 (0.77), TSL was 3.9 (0.84), CPT was 9.67 (1.67), HPT was 42.9 (1.3), TDT was 2.59 (1.38), MPT was 199.5 (39.88), MPS was 0.53 (0.14), WUR was 5.5 (1.44), VDT was 7.59 (1.9 Hz), PPT was 702 (178.27) and DMA was 0.00 (0.00) Data were represented as mean (SEM)
Munts et al. [70]	Consecutive patients with CRPS type 1 (*n* = 44) The mean age was 36 (SD 13 years) and mean disease duration of 10 (6 years) Healthy control women (*n* = 35) Mean age was 40 (SD 13 years)	Diagnosis based on IASP criteria	CDT, WDT, and HPT	Affected and unaffected extremities of CRPS group compared to non-dominant side of healthy controls	In the affected extremity of 44 cases with CRPS, CDT was 30.7 (1.5) in the hand while in the foot, it was 26.7 (5.3). WDT of the hand was 34.7 (3.7) while in the foot, it was 41 (7) and HPT of the hand was 42.3 (10.5) while in the foot, it was 43.2 (9.6) In the unaffected extremity of 7 cases with CRPS, CDT of the hand was 31.6(0.5) while in the foot, it was 30.6(1.5). WDT of the hand was 32.8(1.1) while in the foot, it was 38(8.7) and HPT of the hand was 45.7(4) while in the foot, it was 45.8(4) In the dominant side of 35 healthy controls, CDT of the hand was 31.7 (0.3) while in the foot, it was 31.2 (0.9). WDT of the hand was 32.5 (0.3) while in the foot, it was 35.3 (3) and HPT was 44.6 (3) in the hand while in the foot, it was 45.2 (3.2) Data were represented as median (IQR), while we used Wan’s method to transform data into mean (SD)
Becerra et al. [41] (children)	Lower limb CRPS (*n* = 26) The Age ranged from 10 to 18 years Age and gender matched healthy controls (*n* = 12)	Diagnosis bases on neurological examination and comprehensive record review	HPT, CPT, and allodynia	The affected area of CRPS group and the matched area in the control group	In the affected area of CRPS, HPT was 41.7 (1.2), CPT was 21.2 (3.2), and allodynia was 7 (0.8) In the control group, HPT was 40.5 (3.2), CPT was 11.2 (2.6), and allodynia was 0.2 (0.18)
Habig et al. [71]	CRPS (*n* = 10) Mean age was 33 years, SEM 3.3) Healthy control group: (*n* = 11) Mean age was 43.2 years, SEM 3.9)	The revised Budapest diagnostic criteria	Pleasantness levels after C-tactile perception	The affected area and the matched contralateral area	In the affected area of CRPS, pleasantness levels were 1.85 (2.2) and in the contralateral matched area was 3.4 (0.13) In the control group, pleasantness levels were 3.4 (0.37)
Gossrau et al. [72]	CRPS type 1 (*n* = 19) Mean age was 56.5 (SD 13.4 years) Healthy controls (*n* = 22) Mean age was 60.8 (SD 11.4 years)	Budapest diagnostic criteria	Pleasantness levels after C-tactile perception	The affected area and the matched contralateral area	In the affected area of CRPS, pleasantness levels were 1.94 (1.45) and in the contralateral matched area was 1.96 (1.53) In the control group, pleasantness levels were 3.7 (2) In this study, the data were represented in figure two as mean (95% CI), while we used Cochrane guideline to transform data into mean (SD)

*CRPS* complex regional pain syndrome, *CDT* cold detection threshold, *CPT* cold pain threshold, *DMA* dynamic mechanical allodynia, *HPT* heat pain threshold, *MDT* mechanical detection threshold, *MPS* mechanical pain sensitivity, *MPT* mechanical pain threshold, *PHS* paradoxical heat sensation, *PPT* pressure pain threshold, *QST* quantitative sensory testing, *TSL* thermal sensory limen, *VDT* vibration detection threshold, *PDT* pressure detection threshold, *LTT* light touch threshold, *WDT* warm detection threshold, *WUR* wind-up ratio, *EDT* electric detection threshold, *EPT* electric pain threshold, *SD* standard deviation, *CI* confidence interval

### Data management and meta-analysis

The raw data from individual articles were extracted (Table 4), grouped based on the applied measurements (CPT, HPT, PPT, CDT, WDT, TSL, VDT, MDT, MPS, MPT, DMA, WUR, pain area after pinprick hyperalgesia, pain ratings, and CPM), and further clustered according to age into: (1) patients with chronic CRPS type 1 ≥ 18 years and (2) patients with CRPS type 1 < 18 years. For each age group, the outcomes were clustered according to body location into (1) affected area and (2) remote areas away from the affected site. If a cluster of specific measurements contained at least two studies reporting means and standard deviations for patients with CRPS and healthy controls, a meta-analysis was performed [26].

Meta-analysis was conducted using the Review Manager computer program (RevMan 5.4) by Cochrane collaboration. The standardized mean difference (SMD) and the corresponding 95% CI were calculated based on inverse variance weighting [27]. SMD effect size values between 0.2 and 0.5 are regarded as small, 0.5 to 0.8 as medium, and values higher than 0.8 as large [28]. Egger’s regression test was conducted when there were 10 or more effect sizes to assess publication bias [29, 30] and represented graphically by Begg’s funnel plot [31]. If the p value of Egger’s regression test was less than 0.10, it is considered significant. Whenever publication bias was found, we applied the trim and fill method of Duvall and Tweedie to enhance the symmetry through adding the studies supposed to be missed [32]. To assess the heterogeneity, I2 was measured and classified into: 0%–40%: no heterogeneity, 30%–60%: moderate, 50%–90%: substantial, and 75%–100%: considerable [33]. We determined the borderline I2 values based on the magnitude and direction of effects and the strength of evidence for heterogeneity. So, if there is 50% heterogeneity with a narrower confidence interval and a large effect size, the amount of heterogeneity becomes moderate, whereas heterogeneity is substantial with a wide confidence interval and a small effect size. [33].

The overall effect was significant if the p value was less than 0.05. Studies not included in the meta-analysis were reported separately. Sensitivity analyses were performed to account for the studies with high risk of bias based on the NOS assessment.

GRADE assessment was conducted to check for the certainty of obtained results [34, 35]. One author checked the quality of the evidence considering five domains: (i) risk of bias, (ii) inconsistency of results, (iii) indirectness, (iv) imprecision, and (v) publication bias. At the baseline rating, the studies were considered “low-quality” evidence, due to the observational study design, and then, the rating was upgraded or downgraded the ratings based on the judgment for each of the five domains listed above. The overall quality rating of the evidence was classified as high, moderate, low, or very low evidence [34, 35].

A few studies included median and interquartile ranges, and Wan’s method was used to convert this data into mean and SD [36]. Cochrane guidelines formula was used to convert CI and standard error of mean into SD to be added in the meta-analysis [37].

## Results

### Study selection

**Fig. 1:**
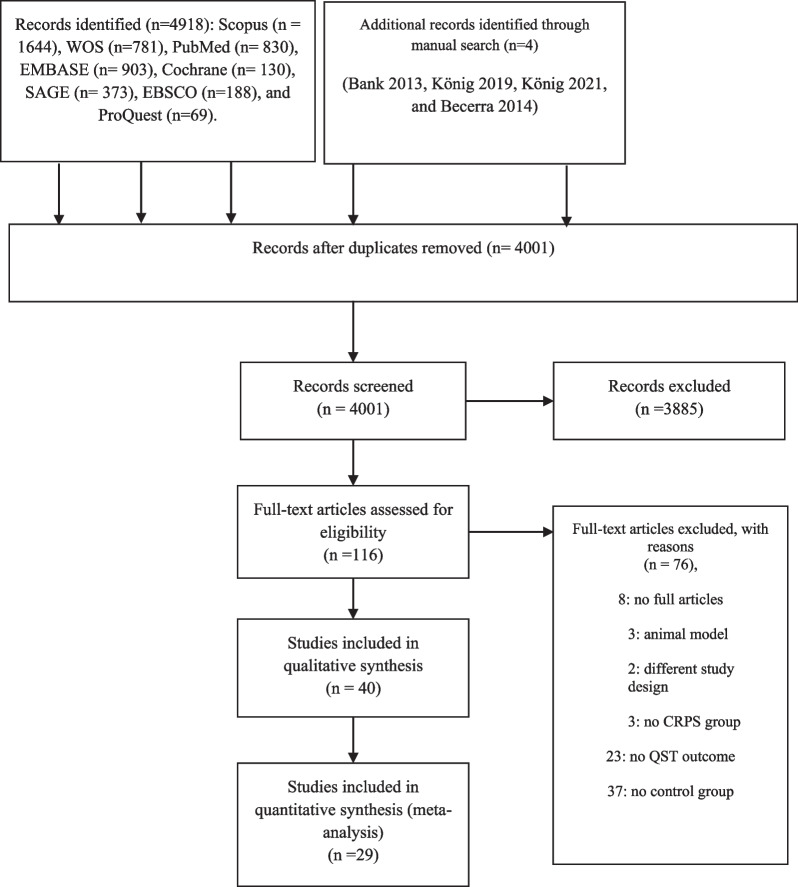
PRISMA flow diagram

The search yielded 4918 articles identified through different databases, with 4 additional studies identified through manual search [38–41]**.** The flowchart of the systematic review is shown in Fig. 1. The titles and abstracts of the remaining articles after removing duplicates were screened (*n* = 4001), and the full texts of 116 articles were read. Forty articles were included in this review [14–16, 18, 38–73, 76] articles were excluded. Reasons for exclusion were: use of animal models (e.g., Ohmichi et al.’s study [74]), different experimental design (e.g., Drummond et al. study [75]), absence of a control group or of a group of individuals with CRPS (e.g., Vaneker et al. study [76]), or inability to obtain the full text (eight studies)**.** The corresponding authors of five publications were contacted requesting data for the meta-analysis [39, 66, 69, 71, 72]. Three authors replied and sent the required information [15, 39, 69].

### Study characteristics

Ten studies were included in the qualitative analysis based on z-scores [14, 39, 40, 53, 61, 66, 68, 71–73], and the frequencies of gain and loss of sensations in CRPS were mentioned in six studies (Table 5) [14, 15, 44, 53, 65, 69]. Twenty-six studies were included in the quantitative analysis. Two studies investigated the sensory profile of patients with CRPS accompanied by dystonia [50, 70], and we included these results in the meta-analysis as we aimed to summarize the sensory profile and underlying pain mechanisms in individuals with CRPS in general. Two studies assessed the level of pleasantness after c-tactile touch perception in CRPS, and we included these results in the meta-analysis to illustrate the functionality of this specific type of C-fibers in CRPS [71, 72].

Rooijen et al. reported the QST results for two groups of individuals with CRPS: one group with dystonia and one group without dystonia [50]. We included the results of both groups in our review. Huge et al. investigated the results of QST in acute and chronic CRPS, but we included only the results of the chronic group in our review [47]. Gierthmühlen et al. described the results of QST for two groups of CRPS (a group with type 1 and the other group with type 2), comparing them to the control group, while we added only the results of QST of CRPS type 1 to the quantitative analysis and after contacting the authors we got the reference values based on Magerl et al. [15, 77]. Kemler et al. reported the results of QST for two groups of individuals with CRPS (one group with upper extremity CRPS and one group with lower extremity CRPS) [44]. We included the results of both groups in our meta-analysis. Thimineur et al. investigated pain ratings after the application of diluted ethanol on the tongue [57]. The results of this study were not included in the meta-analysis of pain ratings after noxious stimulus, because the methods used were very different from the methods used in the other studies. Mainka et al. and Terkelsen et al. [18, 49] assessed both joint and muscle PPTs, which were included in a separate meta-analysis, one related to the muscle and the other to the joint PPTs, respectively.

Uçeyler et al. and Enax-krumova et al. [16, 66] used the same cohort of patients with CRPS and controls. Thus, we added only the results of Uçeyler et al. in the quantitative analysis.

König et al. [40] investigated a subgroup of patients with CRPS that was previously investigated in König et al. [39]. Thus, only the results of König et al. [39] were used in our review.

Two studies investigated the pleasantness level after C-tactile touch perception using brush stroking with a velocity of 3 cm/s both at the affected and contralateral sides. This variable was included in our review, despite addressing a variable not reported in the study protocol, as pleasantness levels could expand our knowledge about the sensory profile and the underlying pain mechanisms in CRPS [71, 72].

Studies that investigated endogenous pain modulation could not be used in the meta-analysis because of different methodological approaches [45, 53]. One study used repetitive electrical stimuli [45], while the other study used a restricted CPM paradigm [53].

### Risk of bias

Quality assessment of the included studies is represented in Table 3, and Kappa statistics for agreement between the two reviewers was 0.76 (95% CI, 0.56–0.95), which is considered substantial agreement [78]. None of the 41 articles included in this review had a score above 7 points out of a maximum score of 9. Most of the included studies were of fair quality as the mean quality score was greater than 4. Only one study reported the non-response rate [18], and all studies had the same ascertainment for cases and controls.

### Sensory profile of adult patients with CRPS

#### Cold detection threshold

Seven studies (one with low quality and six with fair quality), including a total of 505 patients with CRPS, investigated CDT on the affected area [15, 43, 44, 47, 50, 67, 70] and showed a significant loss of cold detection sensation with moderate heterogeneity (Additional file 1: Fig. S1) (Table 6). Furthermore, there was symmetry in the funnel plot of included effect sizes (Additional file 2: Fig. S2).

**Table 5:** Frequencies of sensory gain and loss in CRPS based on QST

Study		CDT	WDT	TSL	CPT	HPT	PPT	MPT	MPS	WUR	MDT	VDT	PHS	DMA
Maier et al. [69]	Gain	2.7%	2.5%	2.7%	30.5%	40.1%	66.3%	28.7%	46.6%	13.1%	9.5%	1.5%	9.4%	24.1%
	Loss	32.5%	26.6%	26.9%	5.2%	7.7%	3.3%	10%	6.2%	2.7%	35.2%	35.4%	–	–
Gierthmühlen[15]	Gain	3.2%	2.1%	3.1%	31.7%	43.7%	66.6%	28.8%	42.8%	14.6%	11.3%	1.7%	6.4%	23.9%
	Loss	29.6%	24.9%	24%	3.7%	6.4%	3.5%	8.9%	9.2%	2.3%	30.9%	32.5%	–	–
Reimer [14]	Gain	0%	0%	5.3%	36.8%	36.8%	100%	5.3%	42.1%	5.3%	5.3%	5.3%	5.3%	26.3%
	Loss	31.6%	26.3%	26.3%	10.5%	5.3%	0%	5.3%	5.3%	6.7%	31.6%	42.1%	–	–
Kemler, [44]	Gain	0%	0%	–	77%	63%	85%	–	–	–	0%	–	–	–
	Loss	36%	27%	–	0%	0%	0%	–	–	–	74%	–	–	–
Dietz et al. [65]	Gain	8%	6.5%	5%	50%	43%	37%	60%	44%	19%	19.5%	–	9.5%	18%
	Loss	46%	42%	50%	30%	22.5%	25%	17.5%	13.5%	14.3%	64%	59.5%	–	–
Kumowski, [53]	Gain	–	–	–	17%	22%	–	33%	–	–	–	–	–	–
	Loss	25%	21%	33%	–	–	–	–	–	–	–	–	–	–

*CRPS* complex regional pain syndrome, *CDT* cold detection threshold, *CPT* cold pain threshold, *DMA* dynamic mechanical allodynia, *HPT* heat pain threshold, *MDT* mechanical detection threshold, *MPS* mechanical pain sensitivity, *MPT* mechanical pain threshold; PHS, paradoxical heat sensation, *PPT* pressure pain threshold, *QST* quantitative sensory testing; TSL, thermal sensory limen, VDT, vibration detection threshold; WDT, warm detection threshold; WUR, wind-up ratio

**Table 6:** Summary of the meta-analysis results

Measurement	Location	Effect size and 95%CI	*p* value	Magnitude of effect size	Heterogeneity	Quality of evidence based on GRADE assessment
Cold detection threshold (adults)	Affected	SMD, − 0.66; 95% CI, − 0.93, − 0.39	*p* < 0.01	Medium	I2 = 60%; *p* = 0.01	Low-quality evidence
	Remote	SMD, − 0.32; 95% CI, − 0.58, − 0.05	*p* = 0.02	Small	I2 = 59%; *p* = 0.01	Low-quality evidence
Warm detection threshold (adults)	Affected	SMD, 0.48; 95% CI, 0.22, 0.73	*p* < 0.01	Small	I2 = 57%; *p* = 0.02	Low-quality evidence
	Remote	SMD, 0.31; 95% CI, 0.06, 0.55	*p* = 0.01	Small	I2 = 51%; *p* = 0.03	Low-quality evidence
Thermal sensory limen (adults)	Affected	SMD, 0.96; 95% CI, 0.62, 1.29	*p* < 0.01	Large	I2 = 65%; *p* = 0.02	Low-quality evidence
	Remote	SMD, 0.61; 95% CI, 0.40, 0.83	*p* < 0.01	Medium	I2 = 48%; *p* = 0.06	Low-quality evidence
Mechanical detection threshold (adults)	Affected	SMD, 0.21; 95% CI, 0.03, 0.40	*p* = 0.02	Small	I2 = 0%; *p* = 0.63	Low-quality evidence
	Remote	SMD, 0.33; 95% CI, 0.14, 0.53	*p* = 0.01	Small	I2 = 1%; *p* = 0.41	Low-quality evidence
Vibration detection threshold (adults)	Affected	SMD, 0.45; 95% CI, 0.17, 0.72	*p* < 0.02	Small	I2 = 31%; *p* = 0.2	Moderate-quality evidence
	Remote	SMD, 0.3; 95% CI, 0.09, 0.52	*P* < 0.01	Small	I2 = 6%; *p* = 0.38	Moderate-quality evidence
Cold pain threshold (adults)	Affected	SMD, 0.75; 95% CI, 0.41, 1.10	*p* < 0.01	Medium	I2 = 72%; *p* < 0.01	Low-quality evidence
	Remote	SMD, 0.36; 95% CI, 0.19, 0.53	*p* < 0.01	Small	I2 = 7%; *p* = 0.38	Moderate-quality evidence
Heat pain threshold (adults)	Affected	SMD, − 0.41; 95% CI, − 0.63, − 0.20	*p* < 0.01	Small	I2 = 45%; *p* = 0.05	Moderate-quality evidence
	Remote	SMD, − 0.30; 95% CI, − 0.46, − 0.15	*P* < 0.01	Small	I2 = 0%; *p* = 0.49	Moderate-quality evidence
Mechanical pain threshold (adults)	Affected	SMD, − 3.66; 95% CI, − 5.95, − 1.37	*p* = 0.01	Large	I2 = 98%; *p* < 0.01	Very low-quality evidence
	Remote	SMD, 0.08; 95% CI, − 0.37, 0.52	*p* = 0.74	Negligible difference	I2 = 0%; *p* = 0.36	Low-quality evidence
Muscle pressure pain threshold (adults)	Affected	SMD, − 1.41; 95% CI, − 1.68, − 1.14	*p* < 0.01	Large	I2 = 52%; *p* = 0.03	Low-quality evidence
	Remote	SMD, − 0.38; 95% CI, − 0.69, − 0.07	*p* = 0.02	Small	I2 = 84%; *p* < 0.01	Low-quality evidence
Joint pressure pain threshold (adults)	Affected	SMD, − 2.92; 95% CI, − 3.47, − 2.37	*p* < 0.01	Large	I2 = 0%; *p* = 0.97	Moderate-quality evidence
	Remote	SMD, − 0.54; 95% CI, − 1.93, 0.86	*p* = 0.45	Medium	I2 = 92%; *p* < 0.01	Low-quality evidence
Mechanical pain sensitivity (adults)	Affected	SMD, 0.59;95% CI, 0.27, 0.9	*p* = 0.02	Medium	I2 = 41%; *p* = 0.15	Low-quality evidence
	Remote	SMD, 0.35; 95% CI, − 0.23, 0.93	*p* = 0.24	Small	I2 = 77%; *p* < 0.02	Very low-quality evidence
Wind up ratio (adults)	Affected	SMD, 0.2; 95% CI, − 0.17, 0.56	*p* = 0.30	Small	I2 = 66%; *p* = 0.01	Low-quality evidence
	Remote	SMD, 0.38; 95% CI, − 0.38, 1.13	*p* = 0.33	Small	I2 = 60%; *p* = 0.11	Low-quality evidence
Pain ratings (adults)	Affected	SMD, 1.29;95% CI, 0.67, 1.91	*p* < 0.01	Large	I2 = 68%; *p* < 0.01	Low-quality evidence
	Remote	SMD, 0.85;95% CI, 0.48, 1.22	*p* < 0.01	Large	I2 = 0%; *p* = 0.59	Low-quality evidence
Area after pinprick hyperalgesia (adults)	Affected	SMD, 3.15;95% CI, 2.13, 4.16	*p* = 0.01	Large	I2 = 54%; *p* = 0.14	Low-quality evidence
Level of pleasantness (adults)	Affected	SMD, − 0.97; 95% CI, − 1.51, − 0.44	*p* = 0.03	Large	I2 = 0%; *p* = 0.99	Moderate-quality evidence
	Remote	SMD, − 0.52; 95% CI, − 1.44, 0.41	*p* = 0.27	Medium	I2 = 67%; *p* = 0.08	Low-quality evidence
Cold detection threshold (children)	Affected	SMD, − 0.85; 95% CI, − 1.62, − 0.08	*p* = 0.03	Large	I2 = 85%; *p* = 0.01	Low-quality evidence
	Remote	SMD, − 0.48; 95% CI, − 1.93, 0.97	*p* = 0.52	Small	I2 = 96%; *p* < 0.01	Low-quality evidence
Warm detection threshold (children)	Affected	SMD, 0.82; 95% CI, − 0.66, 2.29)	*p* = 0.28	Large	I2 = 96%; *p* < 0.01	Low-quality evidence
	Remote	SMD, 0.82; 95% CI, − 0.59, − 1.05	*p* = 0.48	Large	I2 = 97%; *p* < 0.01	Low-quality evidence
Cold pain threshold (children)	Affected	SMD, 1.23;95% CI, 0.05, 2.41	*p* = 0.04	Large	I2 = 93%; *p* < 0.01	Low-quality evidence
	Remote	SMD, − 0.06; 95% CI, − 0.33, 0.22	*p* = 0.67	Negligible difference	I2 = 0%; *p* = 56	Low-quality evidence
Heat pain threshold (children)	Affected	SMD, − 0.05; 95% CI, − 1.09, 0.99	*p* = 0.92	Negligible difference	I2 = 93%; *p* < 0.01	Low-quality evidence
	Remote	SMD, 0.67; 95% CI, − 1.68, 3.02	*p* = 0.58	Medium	I2 = 98%; *p* < 0.01	Low-quality evidence

*CI* confidence interval,* SMD* standardized mean difference

Six studies (one with low quality and five with fair quality), including a total of 245 patients with CRPS, investigated CDT [43, 44, 47, 51, 67, 70] in areas remote from the affected area showing a significant loss of cold sensation with moderate heterogeneity (Additional file 3: Fig. S3) (Table 6). Also, there was no significant publication bias (*p* = 0.9) (Additional file 4: Fig. S4).

Seven studies (two with low quality and five with fair quality) using z-scores to investigate CDT showed loss of cold sensation on the affected side [39, 47, 66, 68, 71–73], and two studies (one with low quality and one with fair quality) showed loss of cold sensation on the contralateral limb [39, 47]. One study of fair quality showed no between-group difference [14].

According to the GRADE assessment, there was low-quality evidence suggesting loss of the cold sensation in patients with CRPS, either at the affected site or the remote areas away from the affected site (Table 6).

#### Warm detection threshold

The meta-analysis of seven studies (one with low quality and six with fair quality) including a total of 505 CRPS patients (Additional file 5: Fig. S5) (Table 6) [15, 43, 44, 47, 50, 67, 70] showed a significant loss of warm sensation on the affected site, with moderate heterogeneity. Furthermore, there was symmetry in the funnel plot of included effect sizes (Additional file 6: Fig. S6).

The meta-analysis of six studies (one with low quality and five with fair quality) including a total of 245 CRPS patients for areas remote from the affected area (Additional file 7: Fig. S7) (Table 6) [43, 44, 47, 51, 67, 70] showed a significant loss of warm sensation, with moderate heterogeneity. Also, there was no significant publication bias (*p* = 0.14) (Additional file 8: Fig. S8).

Nine studies (two with low quality and seven with fair quality) using z-scores showed loss of warm sensation at the affected side [14, 39, 47, 53, 66, 68, 71–73], and two studies (one with low quality and one with fair quality) showed loss of warm sensation on the contralateral limb [39, 47].

According to the GRADE assessment, there was low-quality evidence suggesting loss warm sensations in patients with CRPS, either at the affected site or the remote areas away from the affected site (Table 6).

#### Thermal sensory limen

Four studies (one with low quality and three with fair quality) with a total of 659 patients with CRPS showed a significant loss of thermal sensations on the affected regions, with moderate heterogeneity (I2 = 65%; *p* = 0.02) (Additional file 9: Fig. S9) (Table 6) [15, 47, 57, 67].

A meta-analysis of three studies (one with low quality and two with fair quality) with a total of 894 patients with CRPS for areas remote from the affected area showed a significant loss of thermal sensation, with moderate heterogeneity (Additional file 10: Fig. S10) (Table 6) [47, 57, 67].

Eight studies (two with low quality and six with fair quality) using z-scores showed loss of thermal sensations at the affected side [39, 47, 53, 66, 68, 71–73], and two studies (one with low quality and one with fair quality) showed loss of thermal sensations on the contralateral limb [39, 47].

According to the GRADE assessment, there was low-quality evidence suggesting loss of thermal sensations in patients with CRPS, either at the affected site or the remote areas away from the affected side (Table 6).

#### Mechanical detection threshold

A meta-analysis of five studies (three with low quality and two with fair quality) including a total of 513 patients with CRPS showed a significant loss of mechanical detection sensation on the affected regions, without heterogeneity (Additional file 11: Fig. S11) (Table 6) [15, 44, 45, 52, 57].

A meta-analysis of four studies (three with low quality and one with fair quality) with a total of 292 patients with CRPS showed a significant loss of mechanical detection sensation on the remote areas, without significant heterogeneity (Additional file 12: Fig. S12) (Table 6) [44, 45, 52, 57].

Four studies (one with low quality and three with fair quality) using z-scores showed loss of mechanical detection sensation in patients with CRPS [14, 39, 47, 72], and three studies (one with low quality and two with fair quality) showed no between-group differences [66, 68, 73]. Two studies (one with low quality and one with fair quality) showed loss of mechanical detection sensation in the contralateral limb [39, 47].

According to the GRADE assessment, there was low-quality evidence suggesting loss of mechanical detection sensations in patients either at the affected site or the remote areas away from the affected site (Table 6).

#### Vibration detection threshold

A meta-analysis of four studies of fair quality including a total of a total of 385 patients with CRPS showed a significant loss of vibration detection sensation on the affected regions, without significant heterogeneity (Additional file 13: Fig. S13) (Table 6) [15, 38, 50, 67].

A meta-analysis of three studies of fair quality including a total of 163 patients with CRPS reported a significant loss of vibration sensation on areas remote from the affected area, without significant heterogeneity (Additional file 14: Fig. S14) (Table 6) [38, 51, 67].

Six studies (two with low quality and four with fair quality) using z-scores showed loss of vibration sensation on the affected side [39, 47, 66, 68, 72, 73], one study of fair quality showed no between-group difference [14]**,** and two studies (one with low quality and one with fair quality) showed loss of vibration sensation on the contralateral side [39, 47].

According to the GRADE assessment, there was moderate-quality evidence suggesting loss of vibration sensations in patients with CRPS, either at the affected site or the remote areas away from the affected site (Table 6).

#### Cold pain threshold

Seven studies (one with low quality, five with fair quality, and one with good quality) investigated CPT on the affected areas in 481 patients with CRPS showing significant gain of CPT compared to healthy controls, with substantial heterogeneity (Additional file 15: Fig. S15) (Table 6) [15, 18, 43, 44, 47, 50, 67]. Furthermore, there was asymmetry in the funnel plot of included effect sizes (Additional file 16: Fig. S16).

Meta-analysis of six studies (one with low quality, four with fair quality, and one with good quality) including a total of 240 patients with CRPS investigated CPT in areas remote from the affected area and showed a significant gain of CPT in CRPS compared to healthy controls, without significant heterogeneity (Additional file 17: Fig. S17) (Table 6) [18, 43, 44, 47, 51, 67]. There was also no publication bias (*p* = 0.5) (Additional file 18: Fig. S18).

Six studies (one with low quality and five with fair quality) showed a sensory gain of CPT based on z-scores at the affected site of CRPS [39, 47, 53, 68, 71, 72], while three studies (one with low quality and two with fair quality) showed no between-group differences [14, 66, 73] and two studies (one with low quality and one with fair quality) showed a gain of cold pain sensation on the contralateral side [39, 47].

According to the GRADE assessment, there was low-quality evidence suggesting gain of cold pain thresholds in patients with CRPS at the affected site, but at remote areas, there was moderate-quality evidence (Table 6).

#### Heat pain threshold

A meta-analysis of nine studies (one with low quality, seven with fair quality, and one with good quality) including a total of 548 patients with CRPS showed a significant gain of HPT on the affected area of patients with CRPS, with moderate heterogeneity (Additional file 19: Fig. S19) (Table 6) [15, 18, 43, 44, 47, 50, 62, 67, 70]. Furthermore, there was no significant publication bias (*p* = 0.60) (Additional file 20: Fig. S20).

A meta-analysis of eight studies (one with low quality, six with fair quality, and one with good quality) including a total of 288 patients with CRPS reported a significant gain of HPT in areas remote from the affected area, without significant heterogeneity (Additional file 21: Fig. S21) (Table 6) [18, 43, 44, 47, 51, 62, 67, 70]. Also, there was no significant publication bias (*p* = 0.4) (Additional file 22: Fig. S22).

Six studies (one with low quality and five with fair quality) showed a sensory gain of HPT on the affected site using z-scores [14, 39, 47, 68, 71, 72], while two studies (one with low quality and one with fair quality) showed no differences [66, 73] and two studies (one with low quality and one with fair quality) showed a gain of heat pain sensation on the contralateral side [39, 47].

According to the GRADE assessment, there was moderate-quality evidence suggesting gain of heat pain thresholds in patients with CRPS, either at the affected site or the remote areas away from the affected site (Table 6).

#### Mechanical pain threshold

On the affected side, a meta-analysis of four studies (two with low quality and two with fair quality) including a total of 375 patients with CRPS reported a significant gain of MPT in patients with CRPS, with considerable heterogeneity (Additional file 23: Fig. S23) (Table 6) [15, 45, 56, 67].

On the remote areas, a meta-analysis of two studies (one with low quality and one with fair quality) with a total of 47 patients with CRPS and 34 healthy controls showed no group difference, without heterogeneity (Additional file 24: Fig. S24) (Table 6) [45, 67].

Based on z-scores, five studies (two of low quality and three of fair quality) showed a sensory gain of MPT on the affected site in patients with CRPS [39, 47, 68, 72, 73], while three studies of fair quality showed no between-group differences [14, 66, 71] and two studies (one of low quality and one of fair quality) showed a gain of MPT on the contralateral side [39, 47].

According to the GRADE assessment, there was very low-quality evidence suggesting gain of mechanical pain thresholds in patients with CRPS at the affected site, but at remote areas, there was low-quality evidence suggesting that there was no difference (Table 6).

#### Pressure pain threshold

The meta-analysis of nine studies (three with low quality, five with fair quality, and one with good quality) with a total of 507 patients with CRPS showed a significant gain of muscle PPT on the affected site in CRPS, with moderate heterogeneity (Additional file 25: Fig. S25) (Table 6) [15, 18, 38, 48–50, 52, 63, 67]. There was also no significant publication bias (*p* = 0.12) (Additional file 26: Fig. S26).

On the remote areas, a meta-analysis of nine studies (four with low quality, four with fair quality, and one with good quality) investigating muscle PPT showed a significant gain of PPT in CRPS, with substantial heterogeneity (I2 = 84%; *p* < 0.01) (Additional file 27: Fig. S27) (Table 6) [18, 38, 49, 51, 52, 54, 57, 63, 67]. Also, there was a significant publication bias. After adjusting for publication bias, the PPT difference between CRPS and controls was increased (SMD, − 0.44; 95% CI, − 0.55, − 0.12), with no change in the significance level (*p* < 0.01); heterogeneity remained considerable (Additional file 28: Fig. S28).

Eight studies (three with low quality and five with fair quality) using z-scores showed a gain of muscle PPT at the affected site of patients with CRPS [14, 39, 47, 66, 68, 71–73], while at the contralateral side, one study of fair quality showed a gain of PPT in CRPS [47] and another one of low quality showed no difference [39]. Moreover, one study of fair quality showed a significant gain of PPT on the affected side and remote areas including face, chest, abdomen, and back [55]**.**

According to the GRADE assessment, there was low-quality evidence suggesting gain of pressure pain thresholds of the affected muscles in patients with CRPS, either at the affected site or the remote areas away from the affected site (Table 6).

A meta-analysis of two studies (one with low quality and one with good quality) investigating PPT on affected joints reported a significant gain of PPT in CRPS, without significant heterogeneity (Additional file 29: Fig. S29) (Table 6) [18, 49].

In the remote joints, a meta-analysis of two studies (one with low quality and one with good quality) reported no difference of PPT in CRPS, with considerable heterogeneity (Additional file 30: Fig. S30) (Table 6) [18, 49].

According to the GRADE assessment, there was moderate-quality evidence suggesting gain of pressure pain thresholds of the affected joints in patients with CRPS, but at remote joints, there was low-quality evidence suggesting that there was no difference (Table 6).

#### Mechanical pain sensitivity

The meta-analysis of five studies (two with low quality and three with fair quality) including a total of 396 patients with CRPS showed a significant elevation of MPS in CRPS, with moderate heterogeneity (Additional file 31: Fig. S31) (Table 6) [15, 56, 62, 63, 67].

In the remote areas, a meta-analysis of three studies (one with low quality and two with fair quality) showed no difference, with substantial heterogeneity (Additional file 32: Fig. S32) (Table 6) [62, 63, 67].

Five studies (one with low quality and four with fair quality) showed an elevated MPS on the affected site of patients with CRPS based on z-scores [39, 47, 68, 71, 72], while three studies (one with low quality and two with fair quality) showed no differences [14, 66, 73] and two studies (one with low quality and one with fair quality) showed elevated MPS on the contralateral side of CRPS [39, 47].

According to the GRADE assessment, there was moderate-quality evidence suggesting enhanced mechanical pain sensitivity of the affected site in patients with CRPS, but at remote areas, there was very low-quality evidence suggesting that there was no difference (Table 6).

#### Wind-up ratio

A meta-analysis of five studies (one with low quality and four with fair quality) including a total of 374 patients with CRPS found no difference of WUR at the affected area, with moderate heterogeneity (Additional file 33: Fig. S33) (Table 6) [15, 50, 56, 62, 67].

On the remote areas, a meta-analysis of two studies with fair quality investigated WUR in 37 patients with CRPS showed no difference, with moderate heterogeneity (Additional file 34: Fig. S34) (Table 6) [62, 67].

Based on z-scores, four studies (two with low quality and two with fair quality) showed no differences in WUR on the affected site [14, 39, 66, 73] and one study of fair quality showed elevated WUR on the affected area in patients with CRPS [72].

According to the GRADE assessment, there was low-quality evidence suggesting that there was no difference between the levels of wind-up ratio, either at the affected site or the remote areas away from the affected site (Table 6).

#### Pain ratings after the noxious stimulus

A meta-analysis of five studies (three with low quality, one with fair quality, and one with good quality) reported a significant elevation of pain ratings in CRPS on the affected site, with substantial heterogeneity (Additional file 35: Fig. S35) (Table 6) [18, 42, 43, 45, 56].

In the remote areas, a meta-analysis of four studies (two with low quality, one with fair quality, and one with good quality) reported a significant elevation of pain ratings in CRPS, without significant heterogeneity (Additional file 36: Fig. S36) (Table 6) [18, 42, 43, 45].

According to the GRADE assessment, there was low-quality evidence suggesting elevated pain ratings in patients with CRPS, either at the affected site or the remote areas away from the affected site (Table 6).

#### Area after pinprick hyperalgesia

Meta-analysis of two low-quality studies including a total of 47 patients with CRPS showed a significant increase in the area of hyperalgesia on the affected site of patients with CRPS, with moderate heterogeneity (Additional file 37: Fig. S37) (Table 6) [45, 56].

According to the GRADE assessment, there was low-quality evidence suggesting a significant increase in the area of hyperalgesia on the affected site of patients with CRPS (Table 6).

#### Flare area after electric stimulus

Two studies (one with low quality and one with fair quality) investigated flare areas using laser Doppler imaging [45, 58]. Weber et al. showed a significant increase in flare area after the application of electric stimulus, while Seifert et al. showed no difference between patients with CRPS and healthy controls. We could not add the results in the meta-analysis because of the different techniques used; Weber et al. inserted cutaneous microdialysis fiber to assess protein extravasation while blocking the radial and peroneal nerves at the wrist and ankle, respectively. This could interfere with the assessment of the flare area that occurred after inserting the microdialysis fiber. Seifert et al. assessed the flare area before and after electric stimulation of the affected area without inserting the microdialysis fiber or blocking the radial and peroneal nerves.

#### Electric pain threshold and current detection threshold

Two low-quality studies investigated the sensory profile after the application of electric current [45, 59]. Seifert et al. used a 1 Hz electric current to measure both pain and detection thresholds and found no differences between CRPS patients (affected and contralateral sides) and healthy controls [45]. Raj et al. used electric current of different frequencies and showed that 64% of patients with CRPS had abnormal electric pain threshold, while a percentage of 33% showed abnormal current detection threshold on the affected side, with some abnormalities on the contralateral side [59]. Thus, there were inconsistent findings regarding both electric pain and detection thresholds in CRPS, which need further investigations.

#### Dynamic mechanical allodynia

Several studies indicated the presence of DMA in CRPS [15, 42–45, 55, 59, 67, 69].

#### Paradoxical heat sensation

Several studies indicated that PHS is not frequent in CRPS [14, 15, 47, 53, 67, 69, 73].

#### Endogenous pain modulation

Two studies (one with low quality and one with fair quality) investigated endogenous pain modulation in CRPS [45, 53]. One study used conditioned pain modulation and found comparable descending pain modulation in patients with CRPS and controls [53]. Seifert et al. showed enhanced pain facilitation in CRPS after using repetitive electric pulse stimulation [45].

#### Level of pleasantness in CRPS

Two fair-quality studies looked at the pleasantness level following c-tactile touch perception on the affected side, and their meta-analysis revealed that CRPS patients had significantly lower pleasantness levels than healthy controls, without heterogeneity (Additional file 38: Fig. S38) (Table 6) [71, 72].

On the contralateral side, the meta-analysis of two studies of fair quality investigating the pleasantness level after c-tactile touch perception showed no difference in pleasantness level on the contralateral limb of CRPS compared with healthy controls, with moderate heterogeneity (Additional file 39: Fig. S39) (Table 6) [71, 72].

According to the GRADE assessment, there was moderate-quality evidence suggesting a significant reduction of pleasantness levels at the affected site in patients with CRPS, but at remote joints, there was low-quality evidence suggesting that there was no difference (Table 6).

### Sensory profile of children with CRPS

#### Cold detection threshold

The meta-analysis of two fair-quality studies including a total of 76 children with CRPS showed a significant loss of cold sensation on the affected areas of CRPS, with substantial heterogeneity (Additional file 40: Fig. S40) (Table 6) [46, 64].

On the contralateral side, a meta-analysis of two fair-quality studies including a total of 76 children with CRPS showed no difference in CDT between patients with CRPS and controls, with considerable heterogeneity (Additional file 41: Fig. S41) (Table 6) [46, 64].

According to the GRADE assessment, there was low-quality evidence suggesting loss of cold sensations of the affected site in patients with CRPS, but at the contralateral side, there was low-quality evidence suggesting that there was no difference (Table 6).

#### Warm detection threshold

The meta-analysis of two studies with fair quality including a total of 76 children with CRPS reported no difference in warm sensation on the affected areas between patients with CRPS and controls, with considerable heterogeneity (Additional file 42: Fig. S42) (Table 6) [46, 64].

On the contralateral side, a meta-analysis of two fair-quality studies including a total of 76 children with CRPS reported no difference in WDT between patients with CRPS and controls, with considerable heterogeneity (Additional file 43: Fig. S43) (Table 6) [46, 64].

According to the GRADE assessment, there was low-quality evidence suggesting that there was no difference of warm sensations in patients with CRPS, either at the affected site or the contralateral side (Table 6).

#### Cold pain threshold

A meta-analysis of three fair-quality studies including a total of 102 children with CRPS showed a significant gain of CPT on the affected site of CRPS, with considerable heterogeneity (Additional file 44: Fig. 44) (Table 6) [41, 46, 64].

On the contralateral side, a meta-analysis of two fair-quality studies including a total of 76 children with CRPS reported no difference in CPT between patients with CRPS and controls, without significant heterogeneity (Additional file 45: Fig. S45) (Table 6) [46, 64].

According to the GRADE assessment, there was low-quality evidence suggesting gain of cold pain thresholds of the affected site in patients with CRPS, but at the contralateral side, there was low-quality evidence suggesting that there was no difference (Table 6).

#### Heat pain threshold

On the affected side, a meta-analysis of three fair-quality studies including a total of 102 children with CRPS reported no difference in HPT between patients with CRPS and controls, with considerable heterogeneity (Additional file 46: Fig. 46) (Table 6) [41, 46, 64].

On the contralateral side, a meta-analysis of two fair-quality studies including a total of 76 children with CRPS reported no difference in HPT between patients with CRPS and controls, with considerable heterogeneity (Additional file 47: Fig. S47) (Table 6) [46, 64].

According to the GRADE assessment, there was low-quality evidence suggesting that there was no difference of heat pain thresholds in patients with CRPS, either at the affected site or the contralateral side (Table 6).

### Frequencies of sensory abnormalities in adult with CRPS

Regarding the percentage of sensory loss and hyperalgesia, 25% to 33% of patients with CRPS showed a thermal and mechanical sensory loss, between 60 to 100% of patients showed pressure pain hyperalgesia, and 30% to 40% of patients showed thermal hyperalgesia (Table 5) [14, 15, 69].

#### Sensitivity analysis

A sensitivity analysis was carried out, and studies with a high risk of bias were omitted. As a result, *p* values of the effect sizes were not significantly impacted for all outcomes except TSL of remote areas and MPT of the afflicted site, which showed a non-significant difference. Levels of heterogeneity were also not significantly impacted except for CDT of the affected site, WUR of the affected site, pain rating of the affected site, MPT of the affected site, and MPS of the affected site and the remote areas, which showed a significant reduction. However, after adjusting for low-quality studies, levels of heterogeneity of MDT of the affected site and TSL of the remote areas were significantly increased.

## Discussion

This systematic review aimed to summarize the current literature on QST measurements, pain ratings after noxious stimulus, area of pinprick hyperalgesia, and flare area in patients with CRPS to examine the sensory profile and underlying pain mechanisms.

Adult patients with CRPS showed loss of all detection thresholds (CDT, WDT, MDT, VDT, and TSL) compared to controls, both in the affected and contralateral sides. Also, there was a significant gain in CPT, HPT, and PPT both in the affected and remote areas. Furthermore, pain ratings after noxious stimulus showed significant elevation in the affected and contralateral areas, while MPS was elevated in the affected area only. The area of pinprick hyperalgesia was larger in CRPS compared to healthy controls, while the results for flare area were contradictory. The sensory profile of children with CRPS showed loss of cold sensation and cold hyperalgesia in the affected region without apparent sensory deficits at the remote areas away from the affected site.

Interestingly, adult patients with CRPS showed both sensory loss and primary and secondary hyperalgesia for all pain stimuli in the affected and remote areas, which strongly suggests the involvement of central nervous system and central sensitization [79–81]**.** This has also been supported by investigations in CRPS patients, which revealed bilateral structural and functional abnormalities in brain areas important for pain processing, cognition, and motor behavior [79, 81, 82]. Thus, central sensitization can be initiated by the enhanced peripheral sensitization (enhanced local hyperalgesia) [47, 83], or neuroplasticity at the spinal and brain levels (hemisensory abnormalities and increased area after pinprick hyperalgesia) [45, 63, 70, 84, 85], or the release of inflammatory mediators after tissue injury as substance p, bradykinin, calcitonin gene-related peptide, interleukin-1*β*, -2, -6, and tumor necrosis factor-*α* [8, 86, 87]. The diffuse sensory loss discovered in this meta-analysis could be attributed to decreased neurite density in both affected and unaffected sides of CRPS patients, or it could have a central origin [19, 43, 72, 88]. Finally, the reduced pleasantness level in CRPS could indicate loss of small nerve fibers and central nervous system remodeling as the pleasantness levels reduced more in patients with CRPS accompanied with depression and allodynia than those without allodynia and depression [71, 72].

Comparing the sensory phenotype in CRPS with neuropathic pain conditions reveals distinct sensory patterns. In carpal tunnel syndrome, recent study revealed dominant sensory loss localized only to the affected hand area with inconclusive evidence about central sensitization [89]. Also, in different radiculopathies, the sensory loss was localized to maximum pain area and dermatomal area with inconclusive picture about the presence of hyperalgesia [90–92]. Even in migraine, the impaired pain processing was localized to the affected area [93]. Recently, a new study suggested contralateral spread of sensory loss in painful and painless unilateral neuropathy with slightly limited spread of hyperalgesia [94]. In contrast, the sensory loss and thermal and mechanical hyperalgesia in CRPS were diffuse as evidenced by bilateral sensory loss and bilateral reduction of neurite density. Comparing CRPS to other chronic conditions as tendinitis and arthritis, CRPS showed more prominent thermal and mechanical hyperalgesia [95–97]. Comparing CRPS to chronic conditions with unknown etiology such as fibromyalgia shows comparable results both at the level of diffuse sensory loss or hyperalgesia or reduced level of pleasantness after C-tactile perception [52, 98, 99], which could suggest shared pain mechanisms and etiologies. Such findings could support classifying CRPS as a nociplastic pain type instead of neuropathic pain type [100], in agreement with the recent definition and grading system of neuropathic pain and IASP recent classification which excluded CRPS [100–102]. Interestingly, there was evidence of the presence of different comorbidities in CRPS such as sleep disturbances, post-traumatic stress disorder, and increased sensitivity to light and auditory stimuli [6, 12, 103–105] that strongly suggest a nociplastic mechanism for CRPS. Also, the frequency of sensory abnormalities in CRPS is more consistent than the frequencies found in previous studies for neuropathic pain conditions. In carpal tunnel syndrome, the percentage of patients with sensory loss was found to range from 22 to 33%, thermal hyperalgesia from 1 to 45%, and mechanical hyperalgesia from 20 to 45% [92, 106, 107].

Regarding CPM in CRPS, there were two studies discussing endogenous pain modulation in CRPS. One study showed enhanced pain facilitation rather than impaired descending pain inhibition after using repetitive noxious electrical stimuli [45]. The other study showed unimpaired descending pain inhibition when using the restricted CPM paradigm (heat was used as a test stimulus and cold as a conditioning stimulus) [53]. These contradictory results might be explained by the different disease duration (mean duration was 22 months in the study of Seifert et al., while the maximum disease duration was 12 months in the study of Kumowski et al.) and/or by the different procedures of assessment of endogenous pain modulation. Fortunately, offset analgesia is a paradigm which can also assess endogenous pain modulation that showed impaired pain inhibition in patients with CRPS [108].

No difference was found for temporal summation, represented by WUR, between individuals with CRPS and controls both in the affected and the contralateral limb. This might be due to the small cohort of patients with CRPS in the included studies that investigated WUR, except for Gierthmühlen et al. [15], who showed elevated WUR in a large cohort of patients with CRPS. Importantly, the diffuse loss of small nerve fibers bilaterally can cause the absence of WUR both in the affected and the contralateral regions [43]. Interestingly, WUR of CRPS type II (with evidence of nerve injury) showed no difference when compared to the control group [15], similar to the findings of WUR in CTS (median nerve injury) which showed no difference also [89].

Sensory profile of children and adolescents with CRPS showed loss of cold sensation and cold hyperalgesia at the affected region only, indicating less severe form of CRPS in this age group. Interestingly, children and adolescent with CRPS showed better prognosis and improvement than adults with CRPS, which might be related to the less severe sensory abnormalities [109]. Importantly, the findings of sensory profile of children and adolescents with CRPS are based on three studies only, which prevents us from drawing a comprehensive sensory profile.

### Limitations of the review

Since the overall level of certainty ranged from very low to moderate based on the GRADE assessment [34, 35], the results should be regarded with caution. There were various issues that decreased the general level of certainty. At first, the included studies were observational studies with poor to good quality ratings. Second, there was moderate to substantial heterogeneity across the obtained results. Finally, the meta-analysis of several QST outcomes was based on a small number of studies, and the effect sizes occasionally appear small with large confidence intervals.

It is important to highlight that the sensitivity analysis controlling for low-quality studies (meta-analyses were repeated while excluding studies with high risk of bias) showed a non-significant effect either at the levels of heterogeneity or the obtained effect sizes and corresponding p values of most outcomes. Therefore, the degree of heterogeneity seen in the results might not be explained by the risk of bias of the included studies.

Possible causes of heterogeneity might include the different disease duration of CRPS across the included studies (ranging from six months to five years). Disease duration seems to result in different sensory profiles in patients with CRPS [14, 47, 70]. Thus, future studies might consider comparing sensory profiles of patients with CRPS of different durations. This heterogeneity may be also explained by several factors, starting with the diagnostic criteria for CRPS, which were modified to rely on the Budapest criteria [1] rather than the previous IASP standards [110]. Second, based on the predominant pathophysiology, a recent categorization is better able to distinguish between three clusters of individuals with CRPS type 1 and type 2: CRPS of central phenotype, CRPS of peripheral phenotype, and CRPS of mixed phenotype [111]. As a result, limiting the classification of CRPS to type 1 and type 2 may produce inconsistent results. It is interesting to note that the outcomes of this review are comparable to the findings of the one study that looked at the QST outcomes in CRPS type 2 [15]. This could provide credibility to the current division into three phenotypes.

It is noteworthy to mention that some of the included studies recruited a mix of CRPS type 1 and type 2 which might represent a potential cause of heterogeneity. However, the number patients with CRPS type 2 included in these studies was very small. For example, Terkelsen et al. recruited 2 patients with CRPS type 2 and 18 patients with CRPS type 1[18].

The results of the quantitative sensory testing outcomes of adolescents and children with CRPS were only examined in three studies, which limited the conclusions. Therefore, additional research is required to support the findings of the present review.

## Conclusion

A mix of diffuse thermal and mechanical sensory loss and hyperalgesias in the affected and remote areas is the dominant sensory phenotype in CRPS indicating the dominant peripheral and central sensitization as key underlying pain mechanisms. There is some evidence regarding the enhanced pain facilitation more than impaired descending pain inhibition as evident by elevated thermal and mechanical pain ratings and increased areas of pinprick hyperalgesia. Such results could indicate the involvement of small nerve fibers both at the affected and remote areas. Adolescents and children with CRPS showed less severe form of sensory abnormalities as evident with loss of cold detection sensation and cold hyperalgesia at the affected site.

### Future implications of the review

Further research is needed investigating the efficacy of the descending pain inhibition in patients with CRPS, as well as the widespread sensory loss and hyperalgesia, the pleasantness level after C-tactile stimulation, the electric pain and detection thresholds, and the area of pinprick hyperalgesia of the affected site and remote areas.

As evident from this review, there was a diffuse loss of sensation in patients with CRPS. Thus, the previous studies which compared the QST outcomes of the affected area to that of the contralateral healthy side might result in inconsistent findings as well as might hinder the progress in providing better treatment options. We suggest comparing the affected or contralateral side with reference values of healthy subjects or control group, to avoid any bias.

Previous research revealed that the sensory deficits extended from the affected area to the ipsilateral body sites more compared to the contralateral side [84, 85]. Thus, such studies lacked the presence of control group, while we suggest comparing the results of QST in affected areas, areas in the ipsilateral side away from the affected region, and control group. It is noteworthy that Rooijen et al. investigated the sensory deficits in CRPS affected area, contralateral area, and ipsilateral areas away from the affected region but this study included both patients with CRPS with dystonia and without dystonia [51]. Moreover, face area showed specific sensory abnormalities in patients with CRPS [51, 63] which indeed needs further investigations.

A group of CRPS patients had elevated WUR, whereas another group had no difference when compared to healthy controls. Future research will therefore be required to determine the relationship between the decline in small fiber density and the change in WUR, as it is possible that the decline in small fiber density could prevent the change of the WUR.

Finally, in order to inform better treatment options, it is crucial to compare the new classification of CRPS into three phenotypes (central, peripheral, and mixed) with the existing classification into type 1 and 2. The first step is to investigate the sensory profile of CRPS type 2 and compare it to the results of our review. This could indicate the same sensory profiles and the same underlying pain mechanisms. Thus, the necessity to switch over to the new classification would then likely be of vital importance.

## Supplementary Information

**Additional file 1**. Fig. S1 Pooled results of cold detection threshold (CDT) of the affected area. SD: standard deviation, CRPS: complex regional pain syndrome, and Std Mean Difference: standardized mean difference.

**Additional file 2**. Fig. S2 Funnel plot of cold detection threshold of the affected side.

**Additional file 3**. Fig. S3 Pooled results of cold detection threshold (CDT) of the remote areas. SD: standard deviation, CRPS: complex regional pain syndrome, and Std Mean Difference: standardized mean difference.

**Additional file 4**. Fig. S4 Funnel plot of cold detection threshold of the remote areas.

**Additional file 5.** Fig. S5 Pooled results of warm detection threshold (WDT) of the affected area. SD: standard deviation, CRPS: complex regional pain syndrome, and Std Mean Difference: standardized mean difference.

**Additional file 6**. Fig. S6 Funnel plot of warm detection threshold of the affected side.

**Additional file 7**. Fig. S7 Pooled results of warm detection threshold (WDT) of the remote areas. SD: standard deviation, CRPS: complex regional pain syndrome, and Std Mean Difference: standardized mean difference.

**Additional file 8.** Fig. S8 Funnel plot of warm detection threshold of the remote areas.

**Additional file 9**. Fig. S9 Pooled results of thermal sensory limen (TSL) of the affected area. SD: standard deviation, CRPS: complex regional pain syndrome, and Std Mean Difference: standardized mean difference.

**Additional file 10**. Fig. S10 Pooled results of thermal sensory limen (TSL) of the remote areas. SD: standard deviation, CRPS: complex regional pain syndrome, and Std Mean Difference: standardized mean difference.

**Additional file 11**. Fig. S11 Pooled results of mechanical detection threshold (MDT) of the affected area. SD: standard deviation, CRPS: complex regional pain syndrome, and Std Mean Difference: standardized mean difference.

**Additional file 12**. Fig. S12 Pooled results of mechanical detection threshold (MDT) of the remote areas. SD: standard deviation, CRPS: complex regional pain syndrome, and Std Mean Difference: standardized mean difference.

**Additional file 13**. Fig. S13 Pooled results of vibration detection threshold (VDT) of the affected area. SD: standard deviation, CRPS: complex regional pain syndrome, and Std Mean Difference: standardized mean difference.

**Additional file 14**. Fig. S14 Pooled results of vibration detection threshold (VDT) of the remote areas. SD: standard deviation, CRPS: complex regional pain syndrome, and Std Mean Difference: standardized mean difference.

**Additional file 15**. Fig. S15 Pooled results of cold pain threshold (CPT) of the affected area. SD: standard deviation, CRPS: complex regional pain syndrome, and Std Mean Difference: standardized mean difference.

**Additional file 16.** Fig. S16 Funnel plot of cold pain threshold of the affected side.

**Additional file 17**. Fig. S17 Pooled results of cold pain threshold (CPT) of the remote areas. SD: standard deviation, CRPS: complex regional pain syndrome, and Std Mean Difference: standardized mean difference.

**Additional file 18**. Fig. S18 Funnel plot of cold pain threshold of the remote areas.

**Additional file 19.** Fig. S19 Pooled results of heat pain threshold (HPT) of the affected area. SD: standard deviation, CRPS: complex regional pain syndrome, and Std Mean Difference: standardized mean difference.

**Additional file 20**. Fig. S20 Funnel plot of heat pain threshold of the affected side.

**Additional file 21**. Fig. S21 Pooled results of heat pain threshold (HPT) of the remote areas. SD: standard deviation, CRPS: complex regional pain syndrome, and Std Mean Difference: standardized mean difference.

**Additional file 22**. Fig. S22 Funnel plot of heat pain threshold of the remote areas.

**Additional file 23**. Fig. S23 Pooled results of mechanical pain threshold (MPT) of the affected area. SD: standard deviation, CRPS: complex regional pain syndrome, and Std Mean Difference: standardized mean difference.

**Additional file 24**. Fig. S24 Pooled results of mechanical pain threshold (MPT) of the remote areas. SD: standard deviation, CRPS: complex regional pain syndrome, and Std Mean Difference: standardized mean difference.

**Additional file 25**. Fig. S25 Pooled results of pressure pain threshold (PPT) of the affected area (deep tissue PPT). SD: standard deviation, CRPS: complex regional pain syndrome, and Std Mean Difference: standardized mean difference.

**Additional file 26**. Fig. S26 Funnel plot of pressure pain threshold of the affected side.

**Additional file 27**. Fig. S27 Pooled results of pressure pain threshold (PPT) of the remote areas (deep tissue PPT). SD: standard deviation, CRPS: complex regional pain syndrome, and Std Mean Difference: standardized mean difference.

**Additional file 28**. Fig. S28 Funnel plot of pressure pain threshold of the remote areas.

**Additional file 29**. Fig. S29 Pooled results of pressure pain threshold (PPT) of the affected area (joint PPT). SD: standard deviation, CRPS: complex regional pain syndrome, and Std Mean Difference: standardized mean difference.

**Additional file 30**. Fig. S30 Pooled results of pressure pain threshold (PPT) of the remote areas (joint PPT). SD: standard deviation, CRPS: complex regional pain syndrome, and Std Mean Difference: standardized mean difference.

**Additional file 31**. Fig. S31 Pooled results of mechanical pain sensitivity (MPS) of the affected area. SD: standard deviation, CRPS: complex regional pain syndrome, and Std Mean Difference: standardized mean difference.

**Additional file 32**. Fig. S32 Pooled results of mechanical pain sensitivity (MPS) of the remote areas. SD: standard deviation, CRPS: complex regional pain syndrome, and Std Mean Difference: standardized mean difference.

**Additional file 33**. Fig. S33 Pooled results of wind-up ratio (WUR) of the affected area. SD: standard deviation, CRPS: complex regional pain syndrome, and Std Mean Difference: standardized mean difference.

**Additional file 34**. Fig. S34 Pooled results of wind-up ratio (WUR) of the remote areas. SD: standard deviation, CRPS: complex regional pain syndrome, and Std Mean Difference: standardized mean difference.

**Additional file 35**. Fig. S35 Pooled results of pain ratings after noxious stimulus of the affected area. SD: standard deviation, CRPS: complex regional pain syndrome, and Std Mean Difference: standardized mean difference.

**Additional file 36**. Fig. S36 Pooled results of pain ratings after noxious stimulus of the remote areas. SD: standard deviation, CRPS: complex regional pain syndrome, and Std Mean Difference: standardized mean difference.

**Additional file 37**. Fig. S37 Pooled results of area after induced pinprick hyperalgesia of the affected area. SD: standard deviation, CRPS: complex regional pain syndrome, and Std Mean Difference: standardized mean difference.

**Additional file 38**. Fig. S38 Pooled results of pleasantness level of C-tactile perception of the affected area. SD: standard deviation, CRPS: complex regional pain syndrome, and Std Mean Difference: standardized mean difference.

**Additional file 39**. Fig. S39 Pooled results of pleasantness level of C-tactile perception of the remote areas. SD: standard deviation, CRPS: complex regional pain syndrome, and Std Mean Difference: standardized mean difference.

**Additional file 40**. Fig. S40 Pooled results of cold detection threshold (CDT) of the affected area of children and adolescent with CRPS. SD: standard deviation, CRPS: complex regional pain syndrome, and Std Mean Difference: standardized mean difference.

**Additional file 41**. Fig. S41 Pooled results of cold detection threshold (CDT) of the contralateral side of children and adolescent with CRPS. SD: standard deviation, CRPS: complex regional pain syndrome, and Std Mean Difference: standardized mean difference.

**Additional file 42.** Fig. S42 Pooled results of warm detection threshold (WDT) of the affected area of children and adolescent with CRPS. SD: standard deviation, CRPS: complex regional pain syndrome, and Std Mean Difference: standardized mean difference.

**Additional file 43**. Fig. S43 Pooled results of warm detection threshold (WDT) of the contralateral side of children and adolescent with CRPS. SD: standard deviation, CRPS: complex regional pain syndrome, and Std Mean Difference: standardized mean difference.

**Additional file 44**. Fig. S44 Pooled results of cold pain threshold (CPT) of the affected area of children and adolescent with CRPS. SD: standard deviation, CRPS: complex regional pain syndrome, and Std Mean Difference: standardized mean difference.

**Additional file 45**. Fig. S45 Pooled results of cold pain threshold (CPT) of the contralateral side of children and adolescent with CRPS. SD: standard deviation, CRPS: complex regional pain syndrome, and Std Mean Difference: standardized mean difference.

**Additional file 46**. Fig. S46 Pooled results of heat pain threshold (HPT) of the affected area of children and adolescent with CRPS. SD: standard deviation, CRPS: complex regional pain syndrome, and Std Mean Difference: standardized mean difference

**Additional file 47**. Fig. S47 Pooled results of heat pain threshold (HPT) of the contralateral side of children and adolescent with CRPS. SD: standard deviation, CRPS: complex regional pain syndrome, and Std Mean Difference: standardized mean difference.
