# Supplementary figures and images for: Pain mechanisms in complex regional pain syndrome: a systematic review and meta-analysis of quantitative sensory testing outcomes
Source: J Orthop Surg Res. 2023 Jan 2;18:2. doi: 10.1186/s13018-022-03461-2 (PMC9806919; doi:10.1186/s13018-022-03461-2)

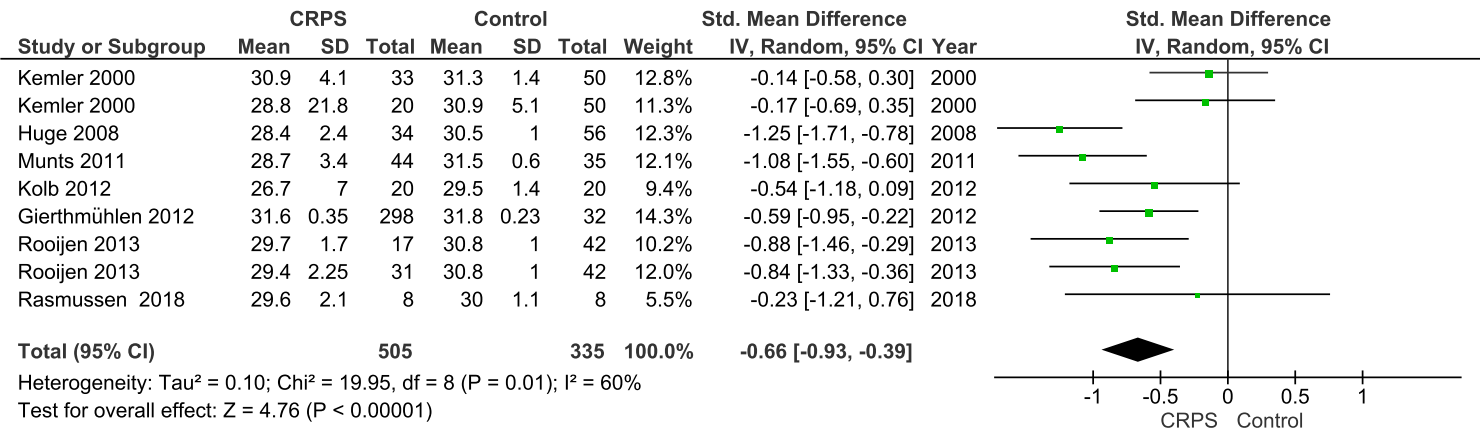

Supplement: Supplementary file 1 — Additional file 1. Fig. S1 Pooled results of cold detection threshold (CDT) of the affected area. SD: standard deviation, CRPS: complex regional pain syndrome, and Std Mean Difference: standardized mean difference. [file 13018_2022_3461_MOESM1_ESM.pdf]

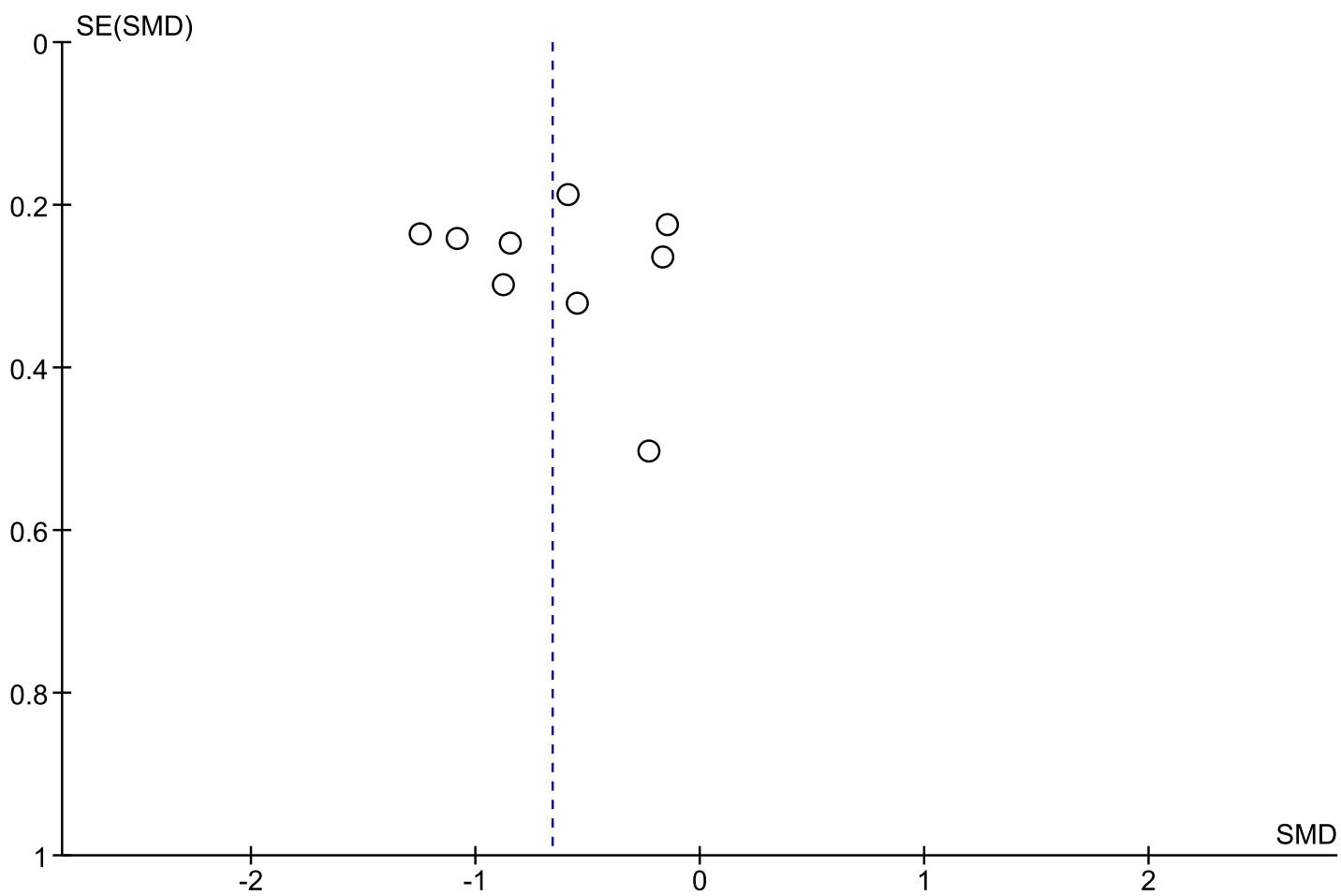

Supplement: Supplementary file 2 — Additional file 2. Fig. S2 Funnel plot of cold detection threshold of the affected side. [file 13018_2022_3461_MOESM2_ESM.pdf]

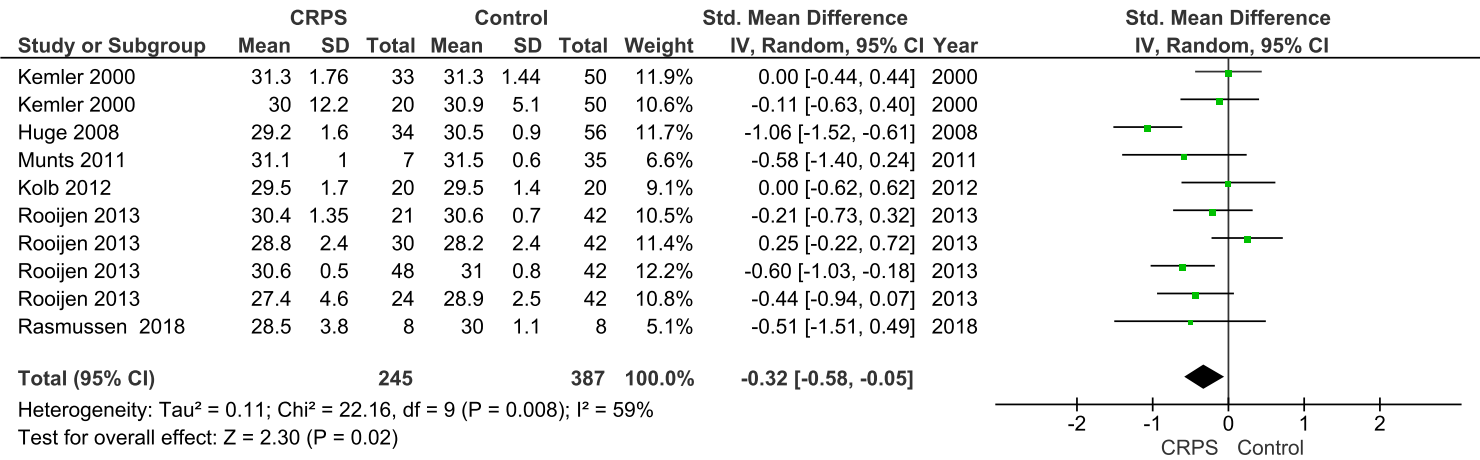

Supplement: Supplementary file 3 — Additional file 3. Fig. S3 Pooled results of cold detection threshold (CDT) of the remote areas. SD: standard deviation, CRPS: complex regional pain syndrome, and Std Mean Difference: standardized mean difference. [file 13018_2022_3461_MOESM3_ESM.pdf]

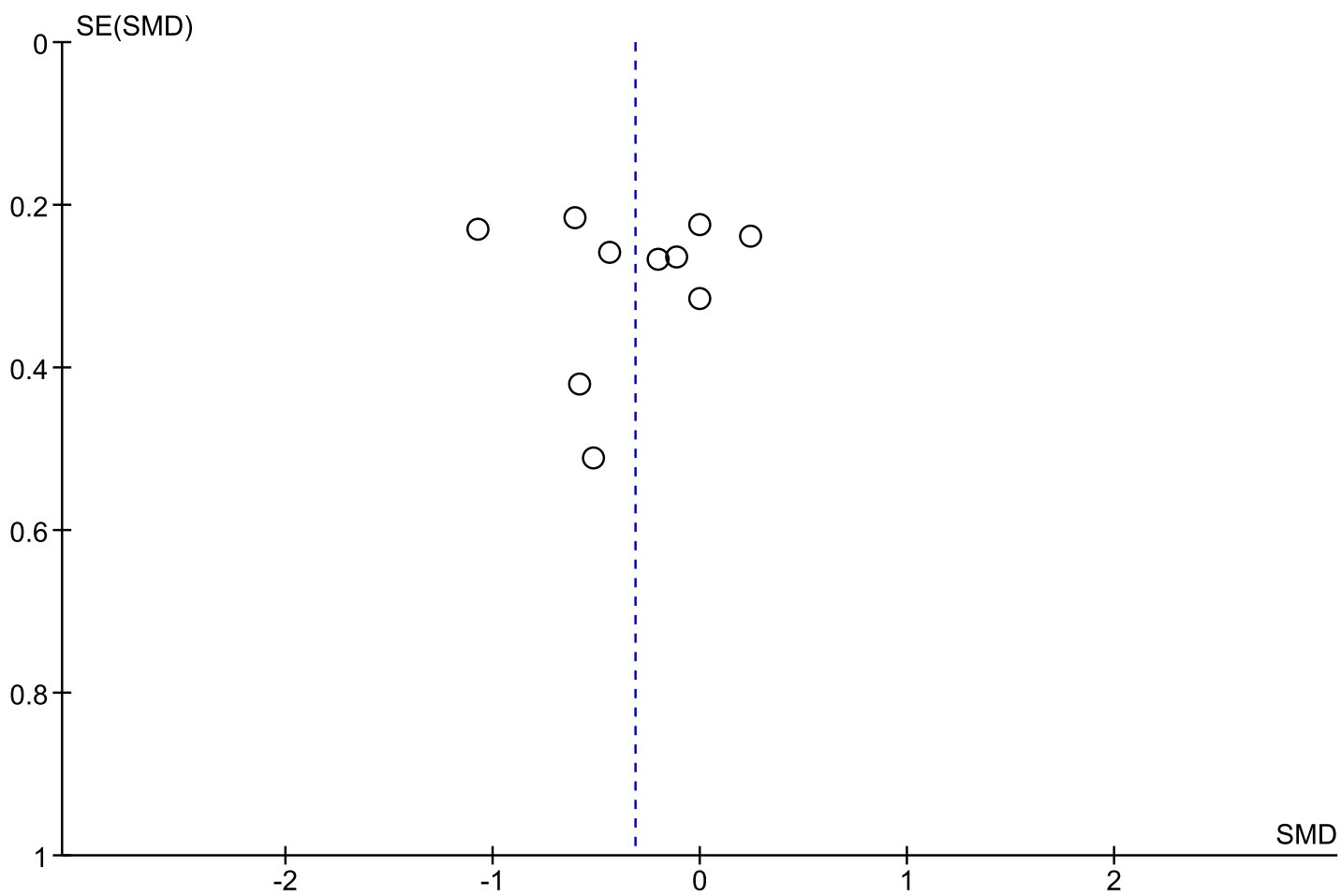

Supplement: Supplementary file 4 — Additional file 4. Fig. S4 Funnel plot of cold detection threshold of the remote areas. [file 13018_2022_3461_MOESM4_ESM.pdf]

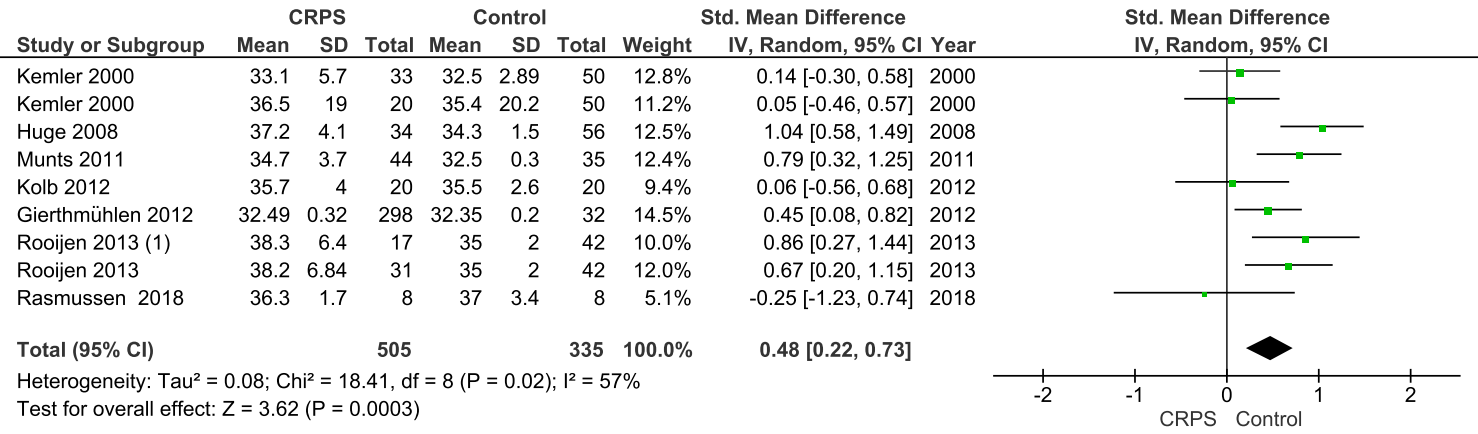

Footnotes  
(1) 6

Supplement: Supplementary file 5 — Additional file 5. Fig. S5 Pooled results of warm detection threshold (WDT) of the affected area. SD: standard deviation, CRPS: complex regional pain syndrome, and Std Mean Difference: standardized mean difference. [file 13018_2022_3461_MOESM5_ESM.pdf]

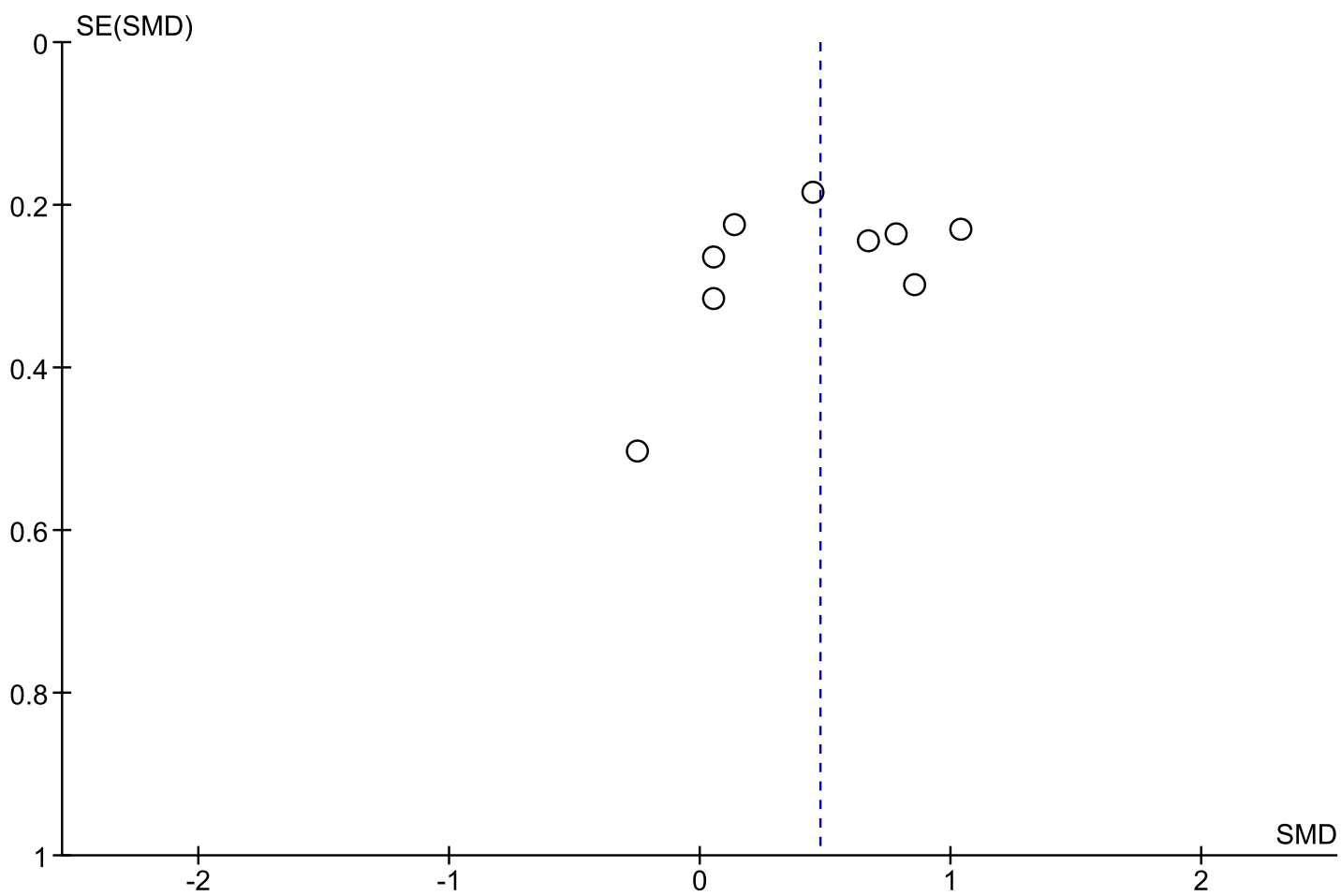

Supplement: Supplementary file 6 — Additional file 6. Fig. S6 Funnel plot of warm detection threshold of the affected side. [file 13018_2022_3461_MOESM6_ESM.pdf]

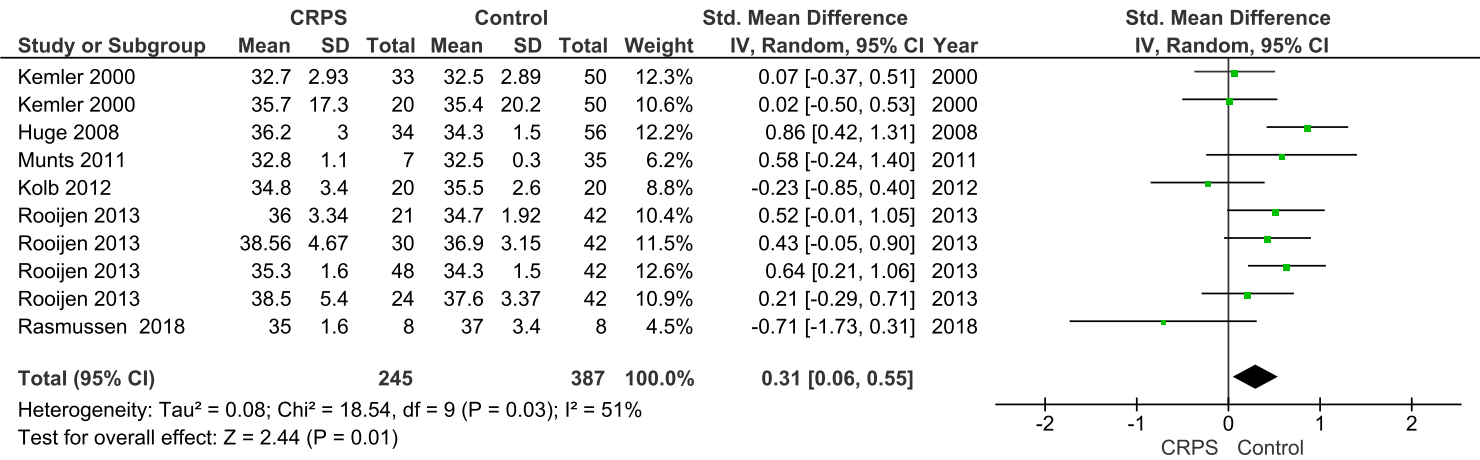

Supplement: Supplementary file 7 — Additional file 7. Fig. S7 Pooled results of warm detection threshold (WDT) of the remote areas. SD: standard deviation, CRPS: complex regional pain syndrome, and Std Mean Difference: standardized mean difference. [file 13018_2022_3461_MOESM7_ESM.pdf]

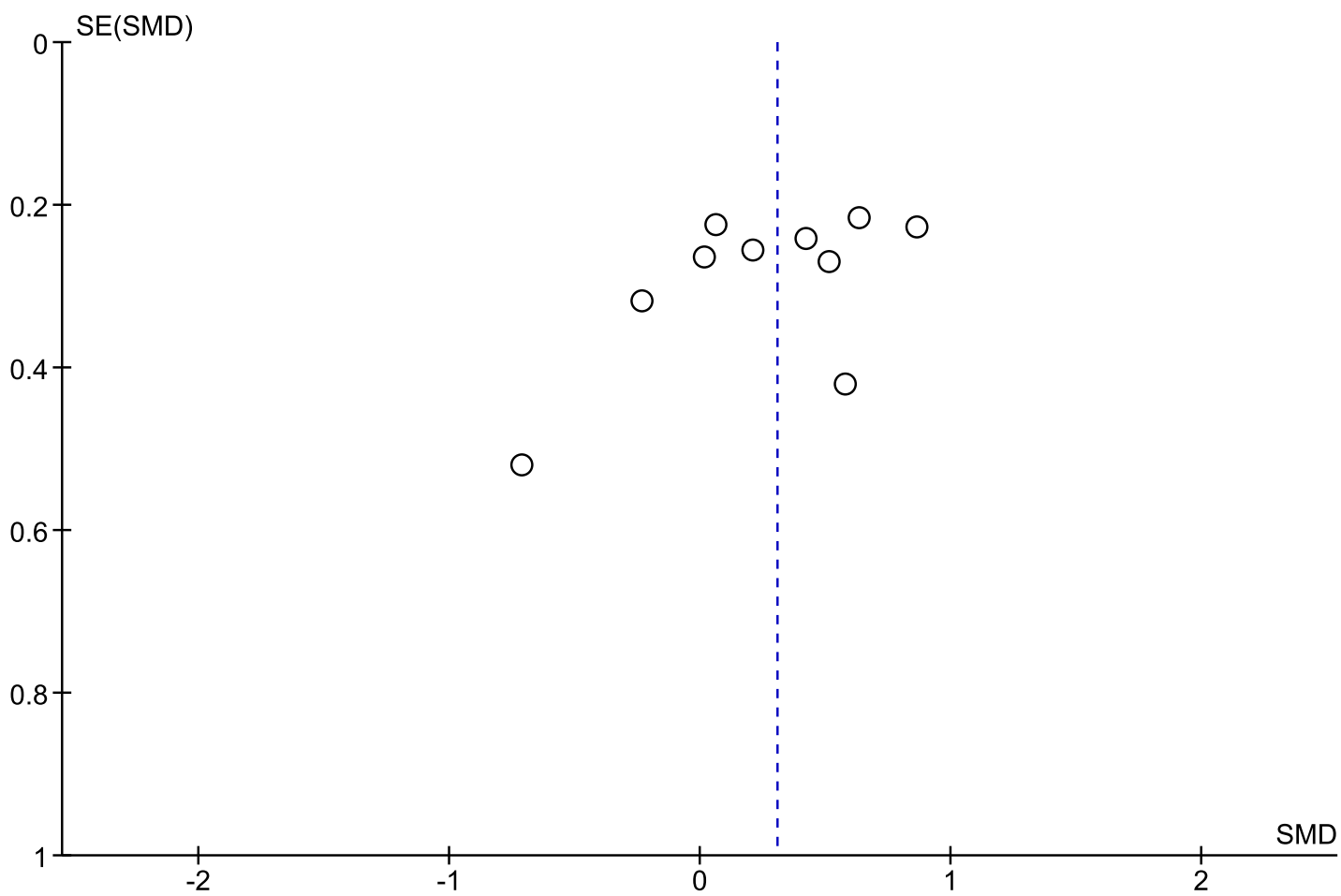

Supplement: Supplementary file 8 — Additional file 8. Fig. S8 Funnel plot of warm detection threshold of the remote areas. [file 13018_2022_3461_MOESM8_ESM.pdf]

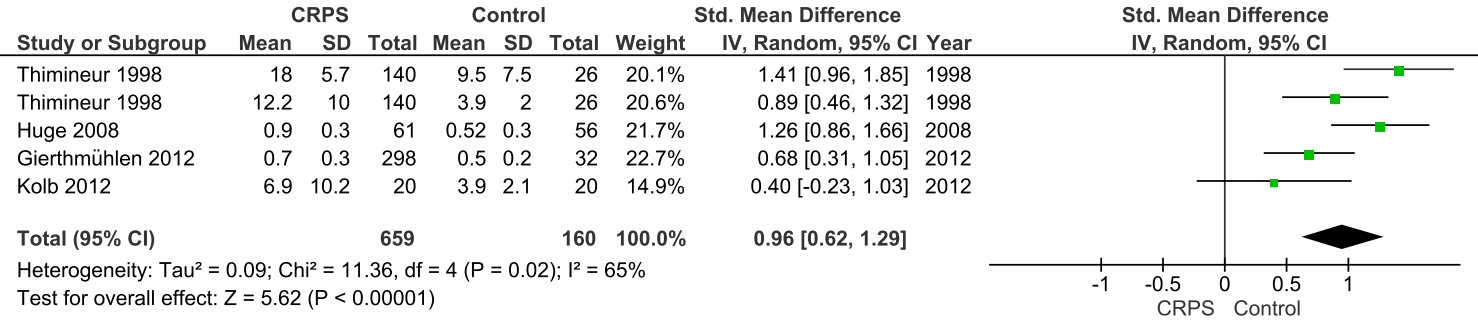

Supplement: Supplementary file 9 — Additional file 9. Fig. S9 Pooled results of thermal sensory limen (TSL) of the affected area. SD: standard deviation, CRPS: complex regional pain syndrome, and Std Mean Difference: standardized mean difference. [file 13018_2022_3461_MOESM9_ESM.pdf]

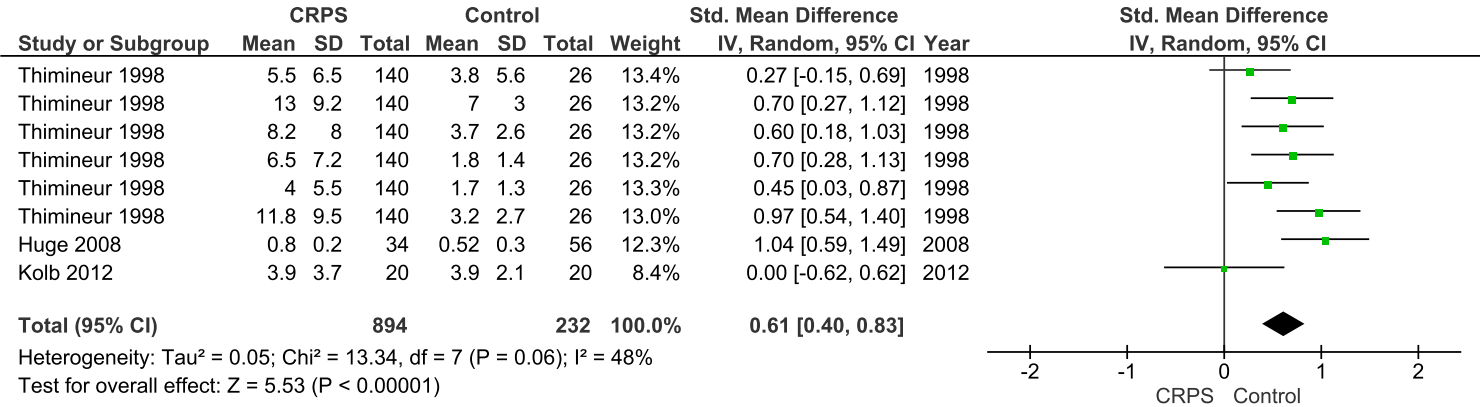

Supplement: Supplementary file 10 — Additional file 10. Fig. S10 Pooled results of thermal sensory limen (TSL) of the remote areas. SD: standard deviation, CRPS: complex regional pain syndrome, and Std Mean Difference: standardized mean difference. [file 13018_2022_3461_MOESM10_ESM.pdf]

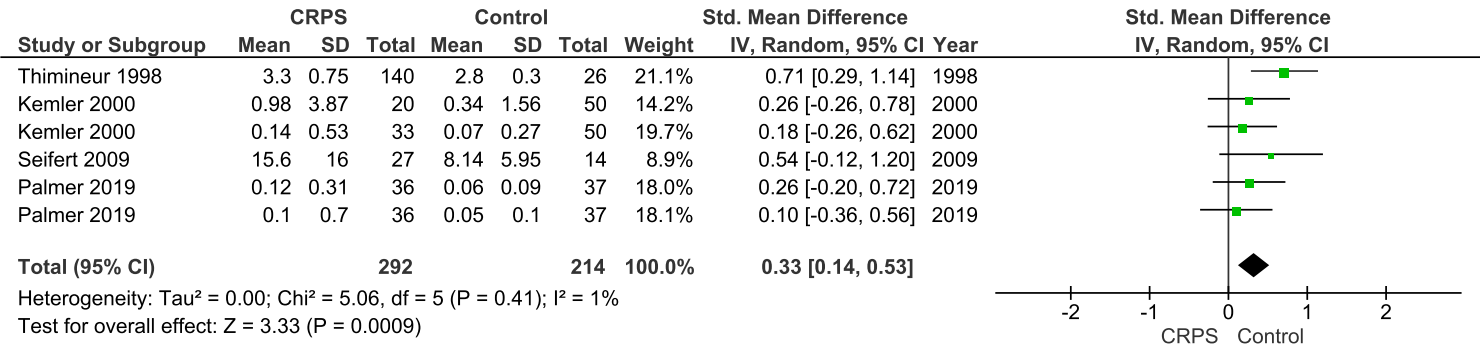

Supplement: Supplementary file 12 — Additional file 12. Fig. S12 Pooled results of mechanical detection threshold (MDT) of the remote areas. SD: standard deviation, CRPS: complex regional pain syndrome, and Std Mean Difference: standardized mean difference. [file 13018_2022_3461_MOESM12_ESM.pdf]

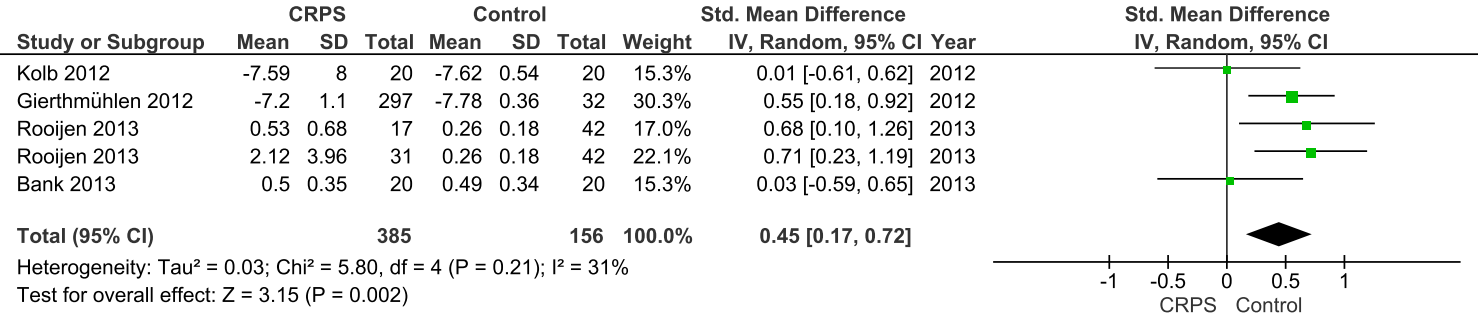

Supplement: Supplementary file 13 — Additional file 13. Fig. S13 Pooled results of vibration detection threshold (VDT) of the affected area. SD: standard deviation, CRPS: complex regional pain syndrome, and Std Mean Difference: standardized mean difference. [file 13018_2022_3461_MOESM13_ESM.pdf]

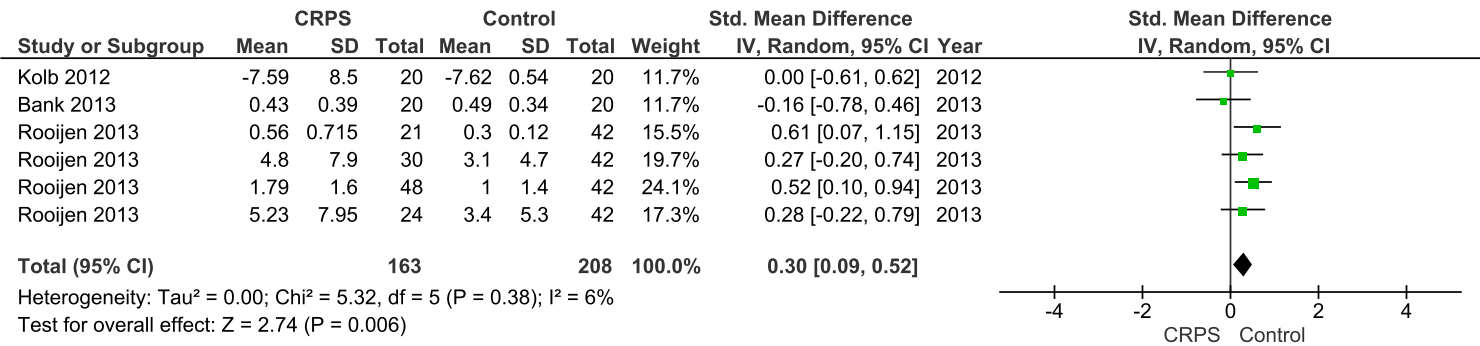

Supplement: Supplementary file 14 — Additional file 14. Fig. S14 Pooled results of vibration detection threshold (VDT) of the remote areas. SD: standard deviation, CRPS: complex regional pain syndrome, and Std Mean Difference: standardized mean difference. [file 13018_2022_3461_MOESM14_ESM.pdf]

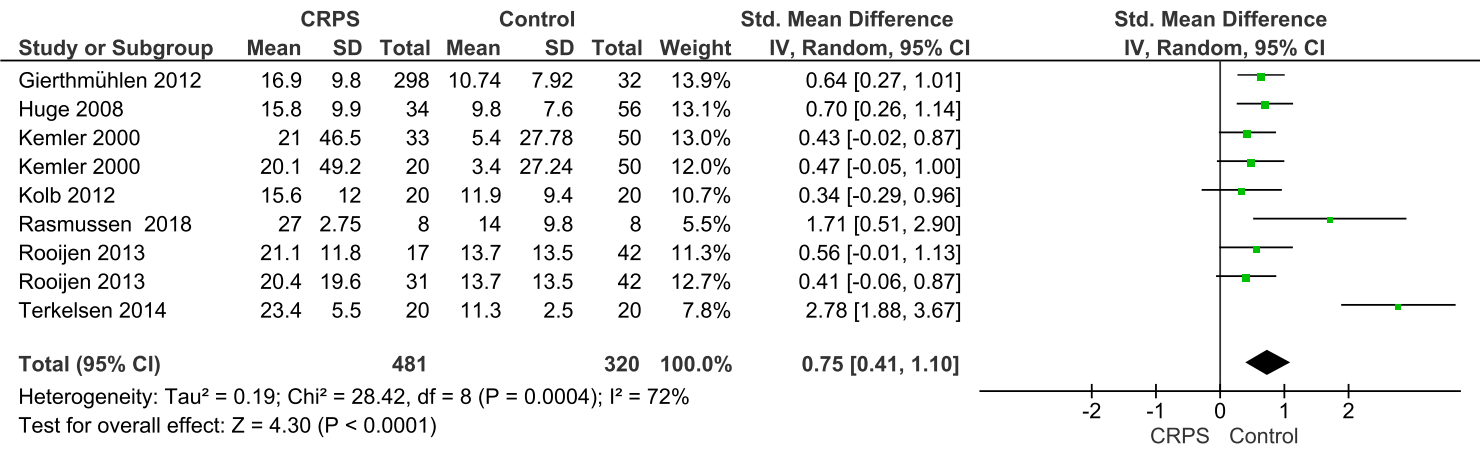

Supplement: Supplementary file 15 — Additional file 15. Fig. S15 Pooled results of cold pain threshold (CPT) of the affected area. SD: standard deviation, CRPS: complex regional pain syndrome, and Std Mean Difference: standardized mean difference. [file 13018_2022_3461_MOESM15_ESM.pdf]

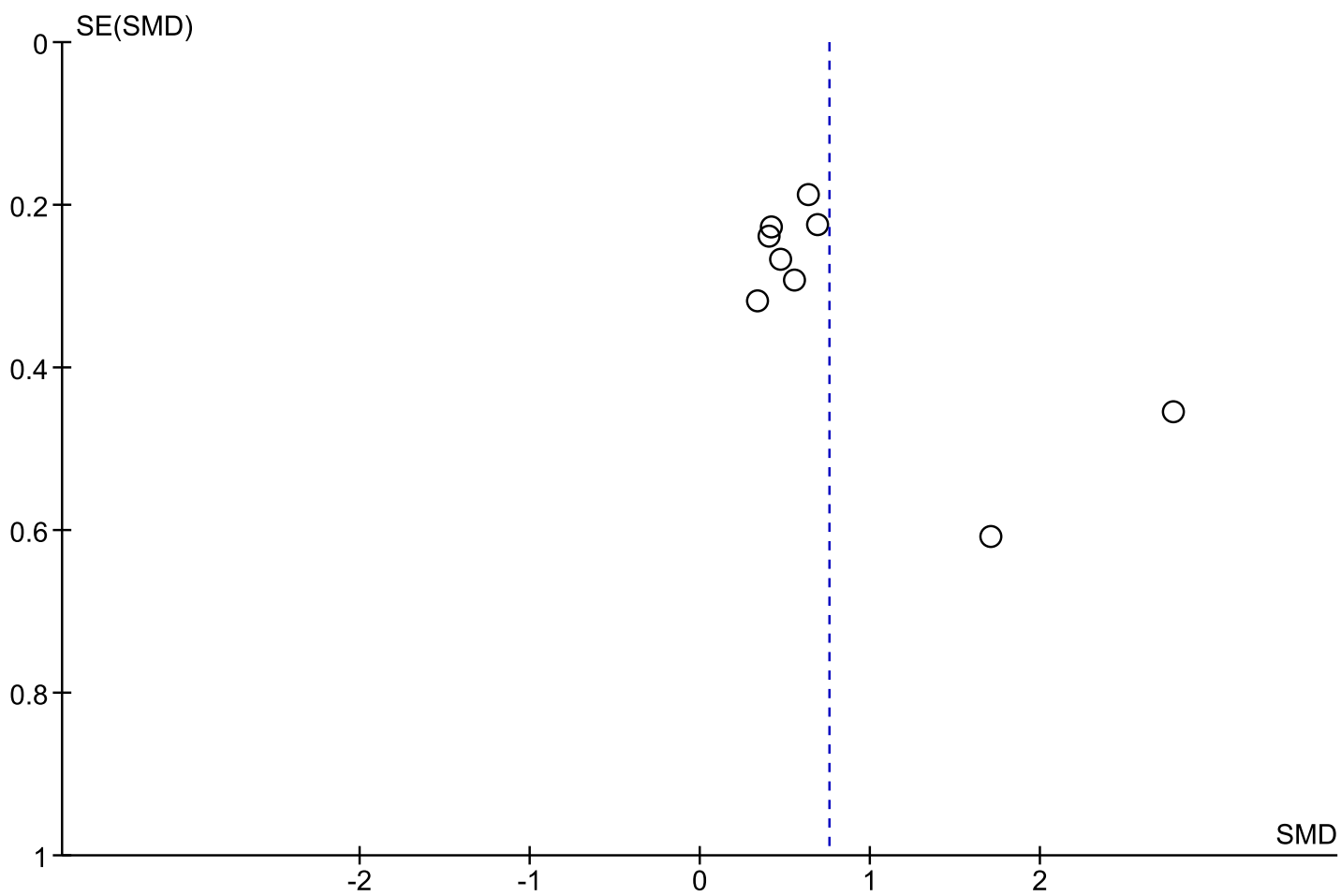

Supplement: Supplementary file 16 — Additional file 16. Fig. S16 Funnel plot of cold pain threshold of the affected side. [file 13018_2022_3461_MOESM16_ESM.pdf]

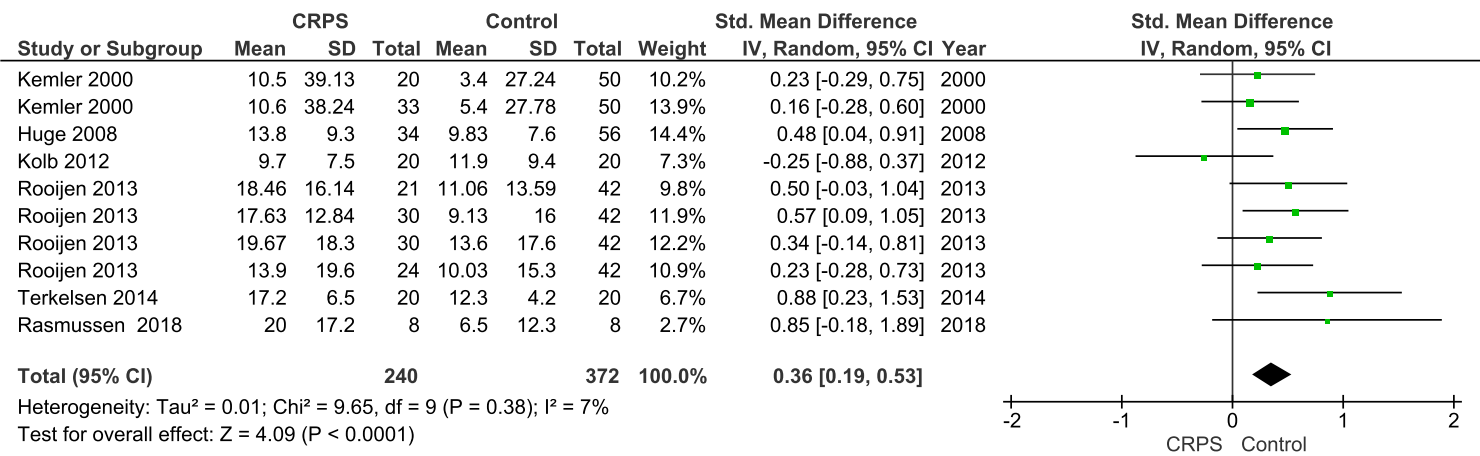

Supplement: Supplementary file 17 — Additional file 17. Fig. S17 Pooled results of cold pain threshold (CPT) of the remote areas. SD: standard deviation, CRPS: complex regional pain syndrome, and Std Mean Difference: standardized mean difference. [file 13018_2022_3461_MOESM17_ESM.pdf]

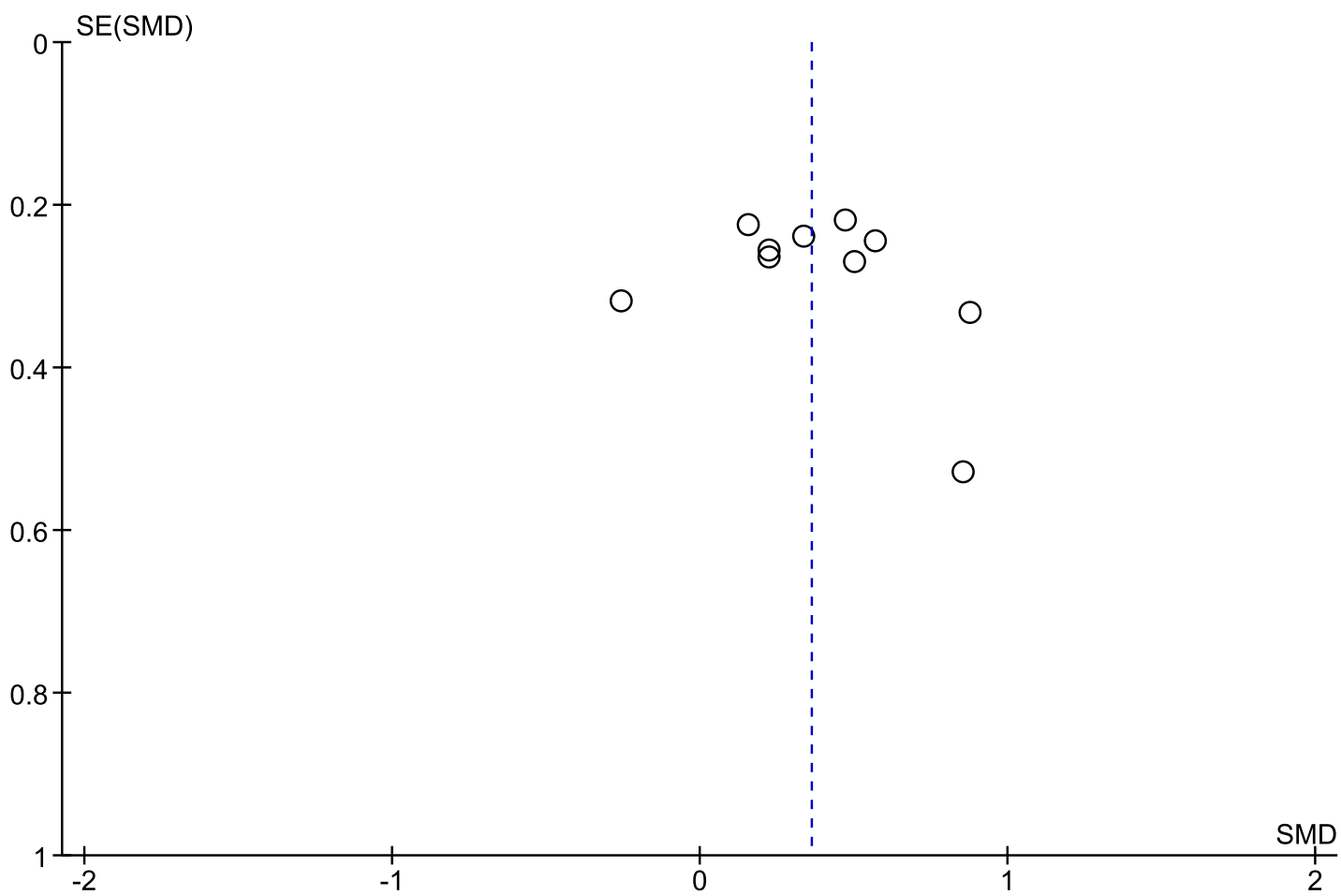

Supplement: Supplementary file 18 — Additional file 18. Fig. S18 Funnel plot of cold pain threshold of the remote areas. [file 13018_2022_3461_MOESM18_ESM.pdf]

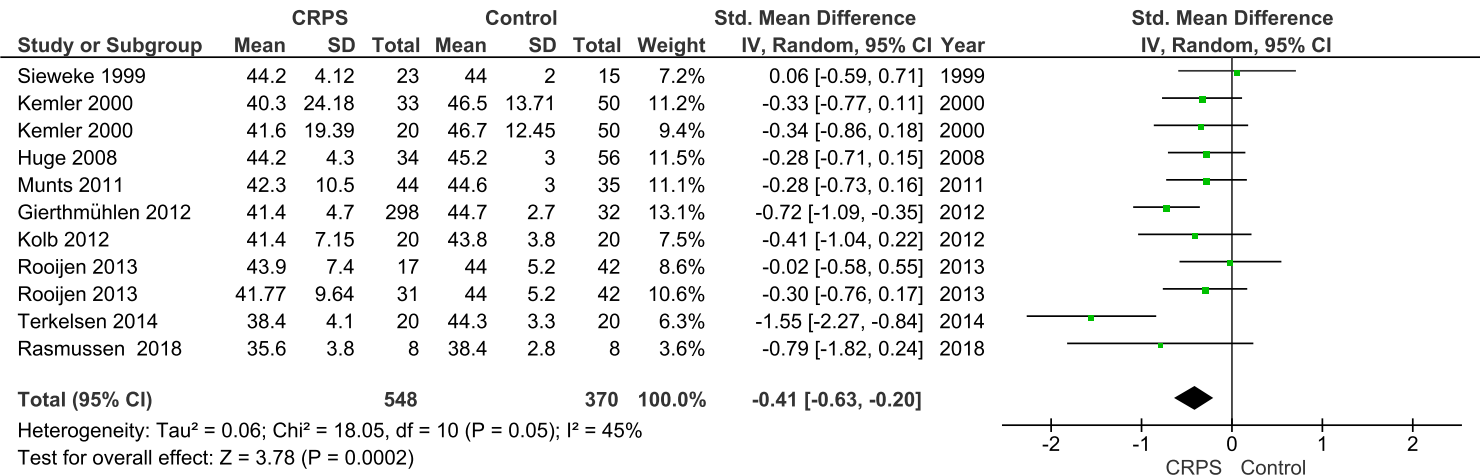

Supplement: Supplementary file 19 — Additional file 19. Fig. S19 Pooled results of heat pain threshold (HPT) of the affected area. SD: standard deviation, CRPS: complex regional pain syndrome, and Std Mean Difference: standardized mean difference. [file 13018_2022_3461_MOESM19_ESM.pdf]

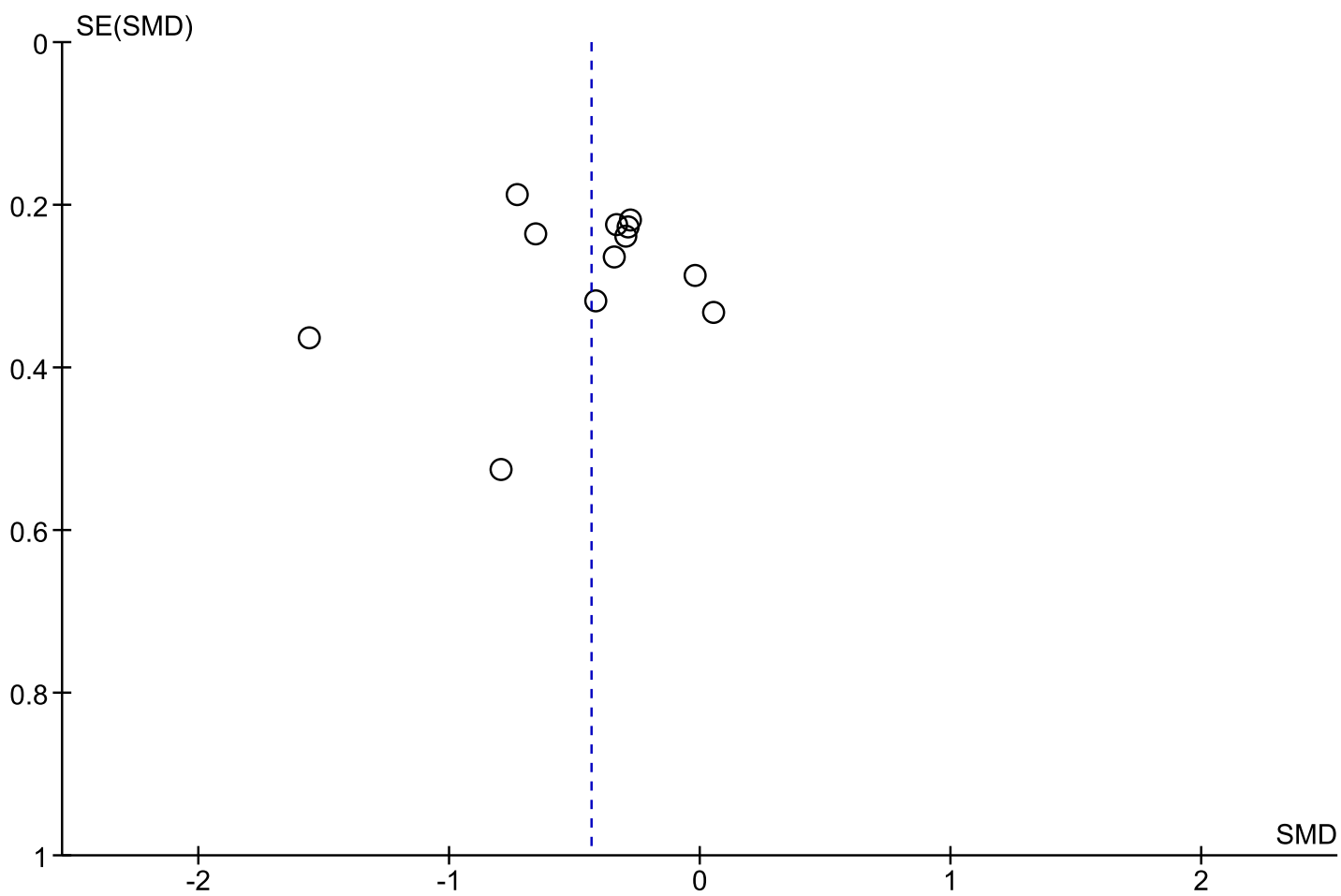

Supplement: Supplementary file 20 — Additional file 20. Fig. S20 Funnel plot of heat pain threshold of the affected side. [file 13018_2022_3461_MOESM20_ESM.pdf]

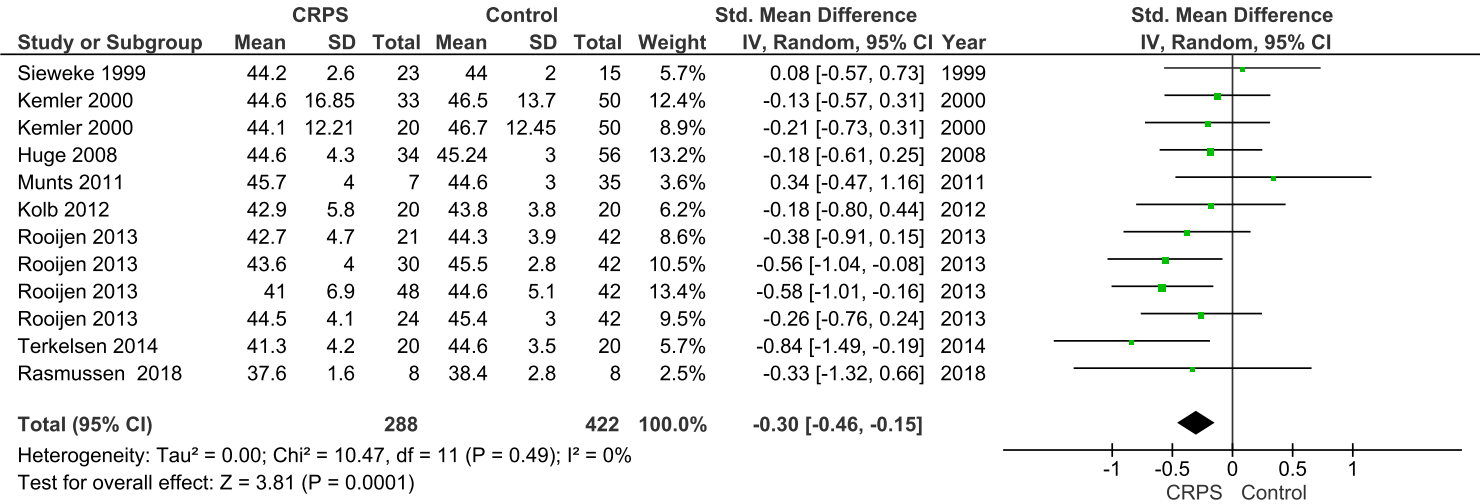

Supplement: Supplementary file 21 — Additional file 21. Fig. S21 Pooled results of heat pain threshold (HPT) of the remote areas. SD: standard deviation, CRPS: complex regional pain syndrome, and Std Mean Difference: standardized mean difference. [file 13018_2022_3461_MOESM21_ESM.pdf]

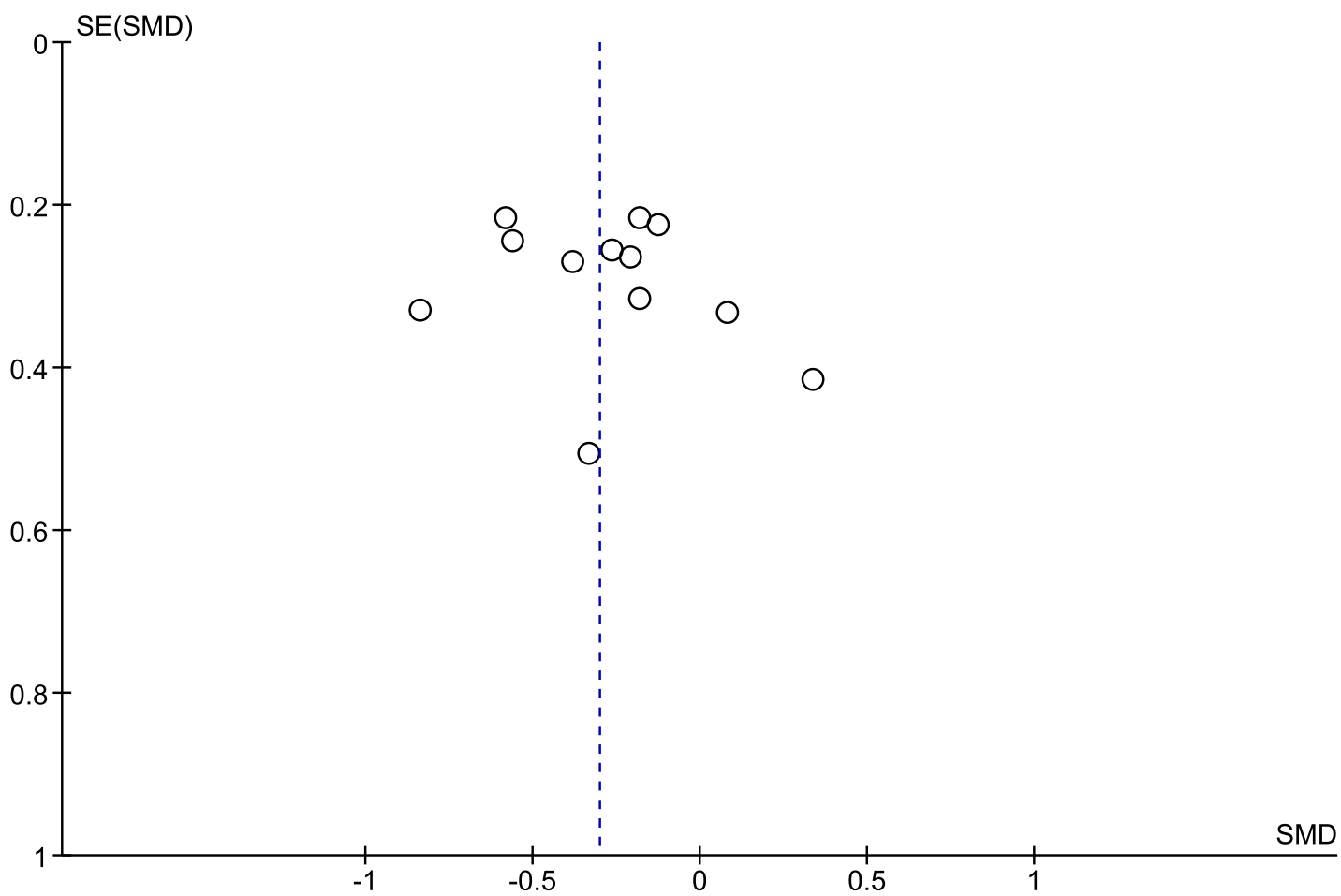

Supplement: Supplementary file 22 — Additional file 22. Fig. S22 Funnel plot of heat pain threshold of the remote areas. [file 13018_2022_3461_MOESM22_ESM.pdf]

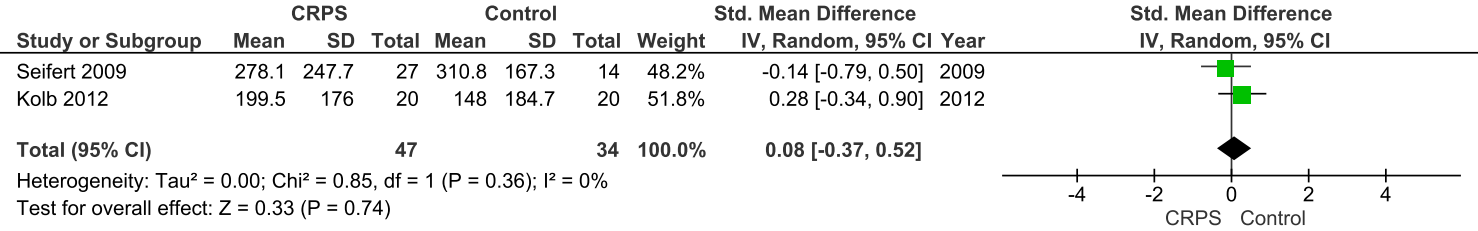

Supplement: Supplementary file 24 — Additional file 24. Fig. S24 Pooled results of mechanical pain threshold (MPT) of the remote areas. SD: standard deviation, CRPS: complex regional pain syndrome, and Std Mean Difference: standardized mean difference. [file 13018_2022_3461_MOESM24_ESM.pdf]

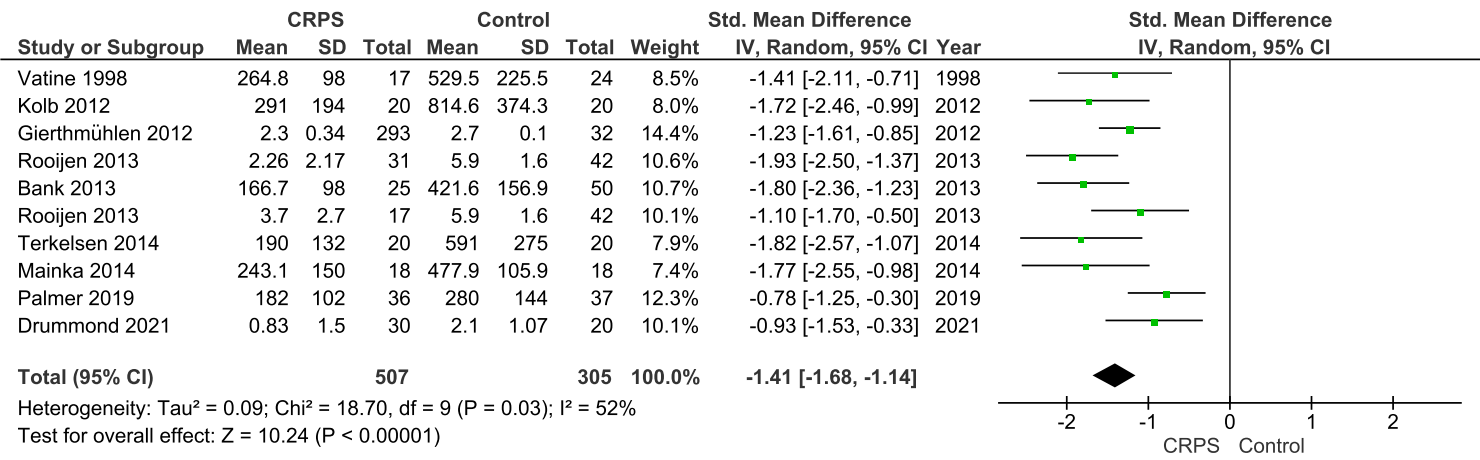

Supplement: Supplementary file 25 — Additional file 25. Fig. S25 Pooled results of pressure pain threshold (PPT) of the affected area (deep tissue PPT). SD: standard deviation, CRPS: complex regional pain syndrome, and Std Mean Difference: standardized mean difference. [file 13018_2022_3461_MOESM25_ESM.pdf]

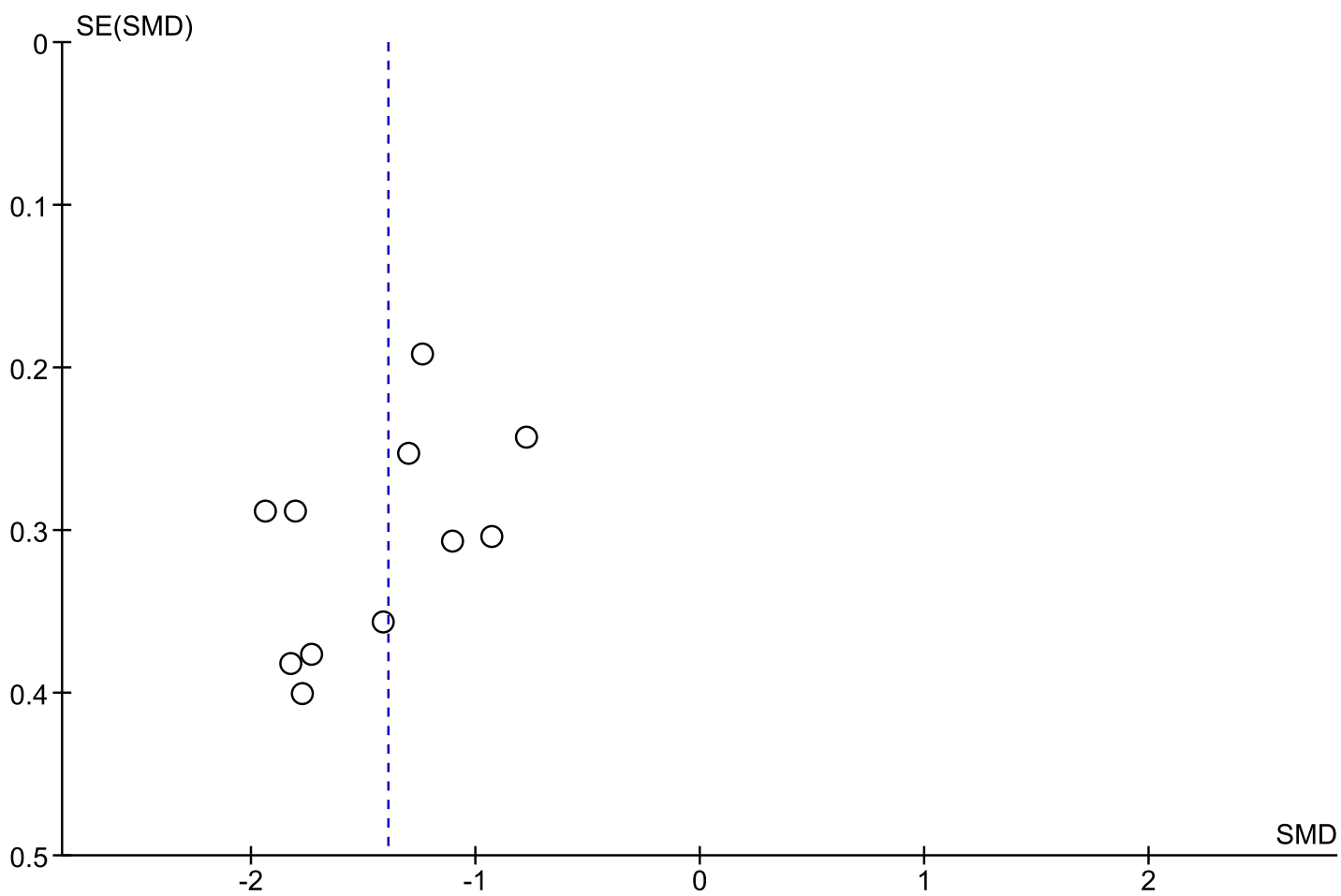

Supplement: Supplementary file 26 — Additional file 26. Fig. S26 Funnel plot of pressure pain threshold of the affected side. [file 13018_2022_3461_MOESM26_ESM.pdf]

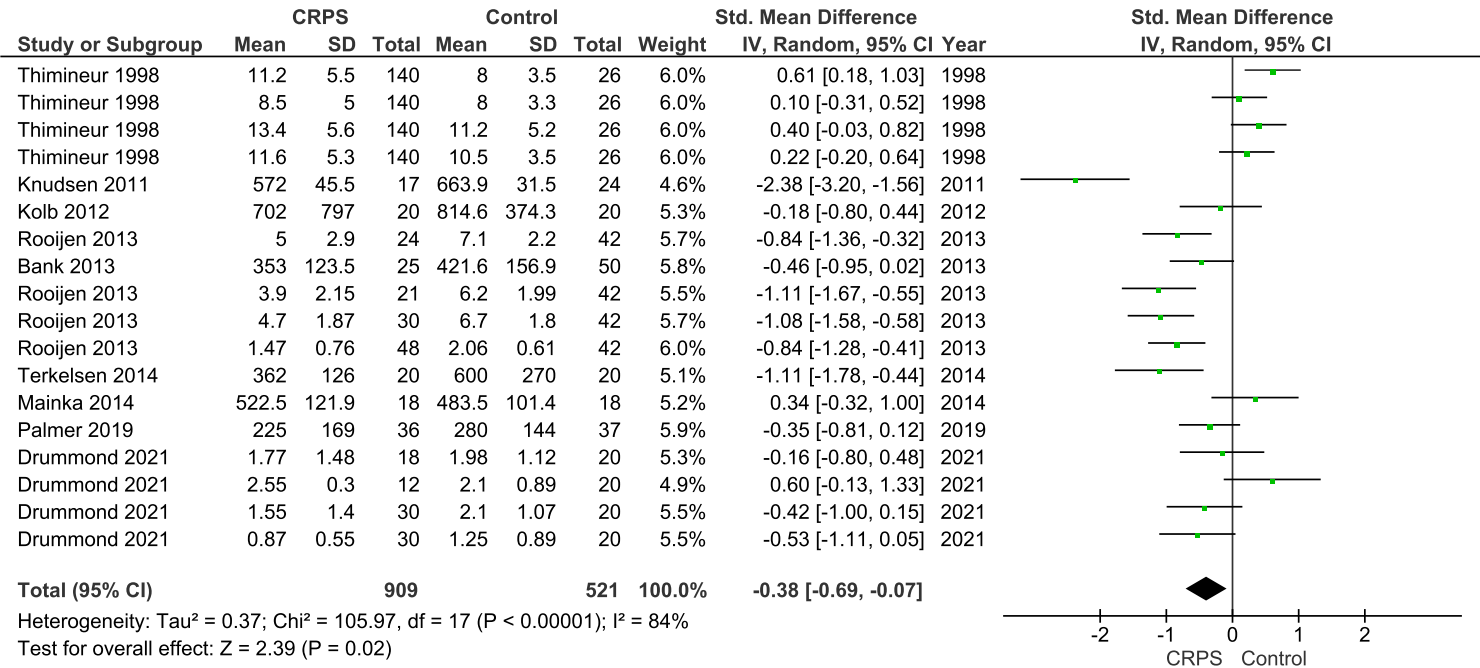

Supplement: Supplementary file 27 — Additional file 27. Fig. S27 Pooled results of pressure pain threshold (PPT) of the remote areas (deep tissue PPT). SD: standard deviation, CRPS: complex regional pain syndrome, and Std Mean Difference: standardized mean difference. [file 13018_2022_3461_MOESM27_ESM.pdf]

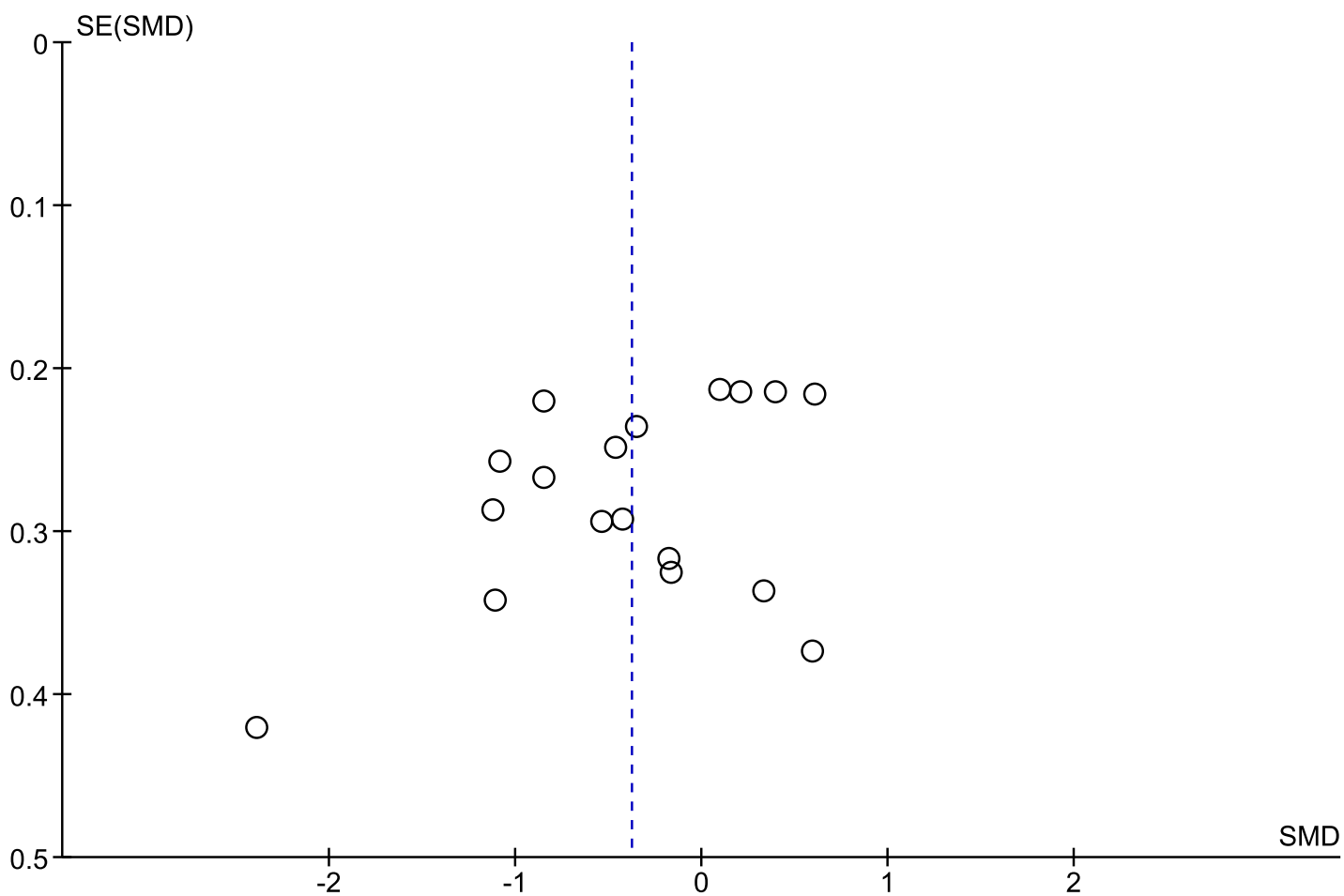

Supplement: Supplementary file 28 — Additional file 28. Fig. S28 Funnel plot of pressure pain threshold of the remote areas. [file 13018_2022_3461_MOESM28_ESM.pdf]

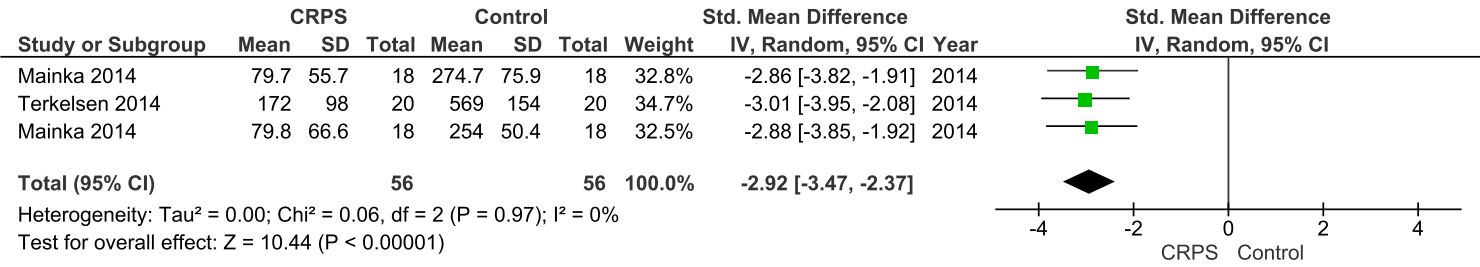

Supplement: Supplementary file 29 — Additional file 29. Fig. S29 Pooled results of pressure pain threshold (PPT) of the affected area (joint PPT). SD: standard deviation, CRPS: complex regional pain syndrome, and Std Mean Difference: standardized mean difference. [file 13018_2022_3461_MOESM29_ESM.pdf]

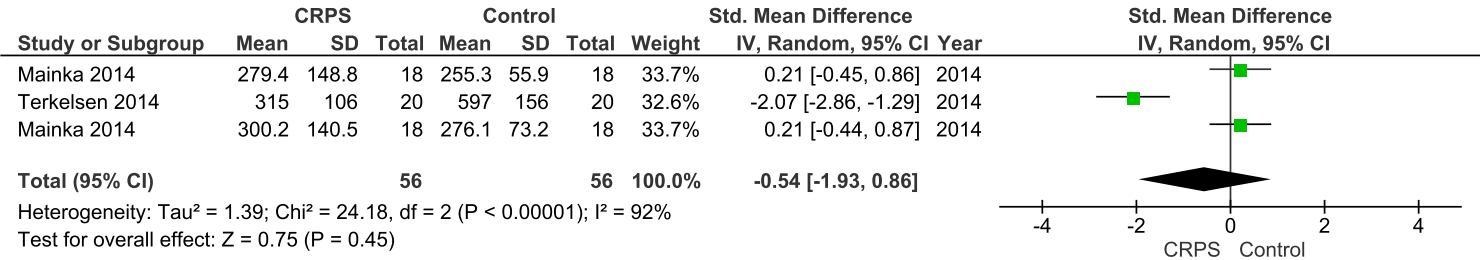

Supplement: Supplementary file 30 — Additional file 30. Fig. S30 Pooled results of pressure pain threshold (PPT) of the remote areas (joint PPT). SD: standard deviation, CRPS: complex regional pain syndrome, and Std Mean Difference: standardized mean difference. [file 13018_2022_3461_MOESM30_ESM.pdf]

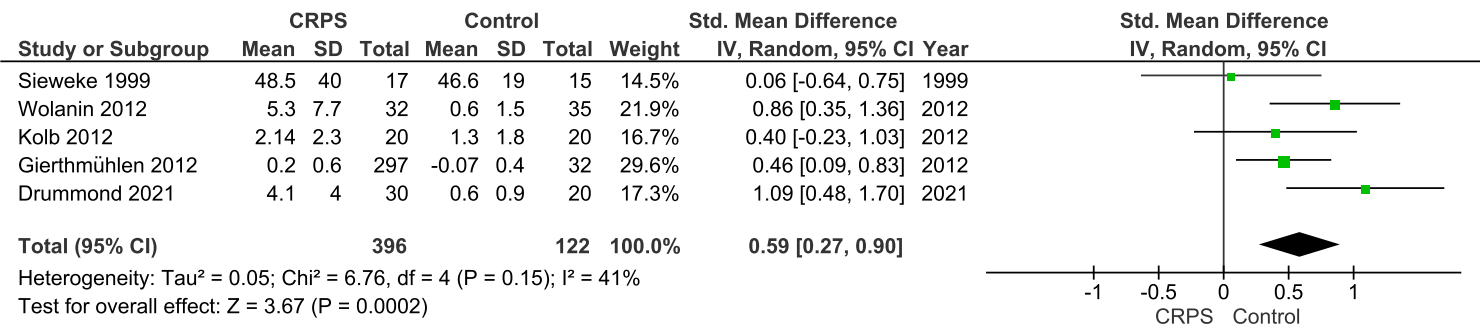

Supplement: Supplementary file 31 — Additional file 31. Fig. S31 Pooled results of mechanical pain sensitivity (MPS) of the affected area. SD: standard deviation, CRPS: complex regional pain syndrome, and Std Mean Difference: standardized mean difference. [file 13018_2022_3461_MOESM31_ESM.pdf]

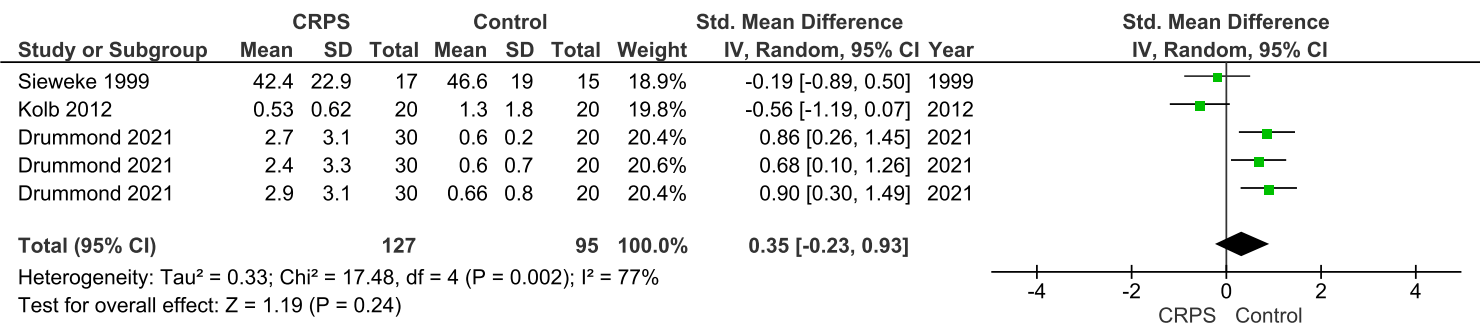

Supplement: Supplementary file 32 — Additional file 32. Fig. S32 Pooled results of mechanical pain sensitivity (MPS) of the remote areas. SD: standard deviation, CRPS: complex regional pain syndrome, and Std Mean Difference: standardized mean difference. [file 13018_2022_3461_MOESM32_ESM.pdf]

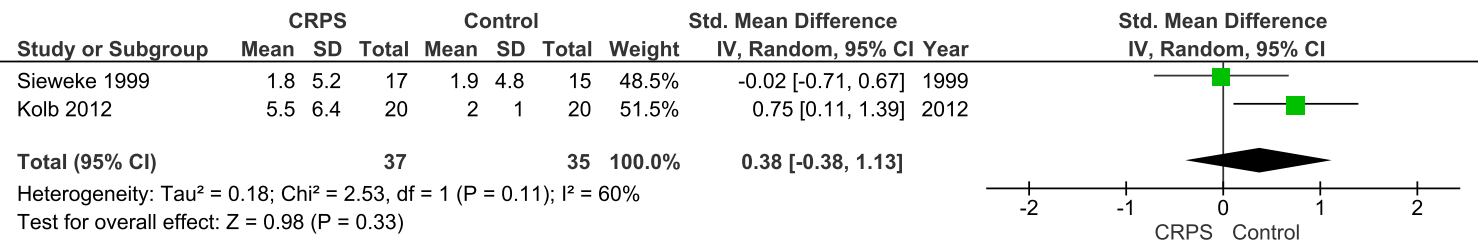

Supplement: Supplementary file 34 — Additional file 34. Fig. S34 Pooled results of wind-up ratio (WUR) of the remote areas. SD: standard deviation, CRPS: complex regional pain syndrome, and Std Mean Difference: standardized mean difference. [file 13018_2022_3461_MOESM34_ESM.pdf]

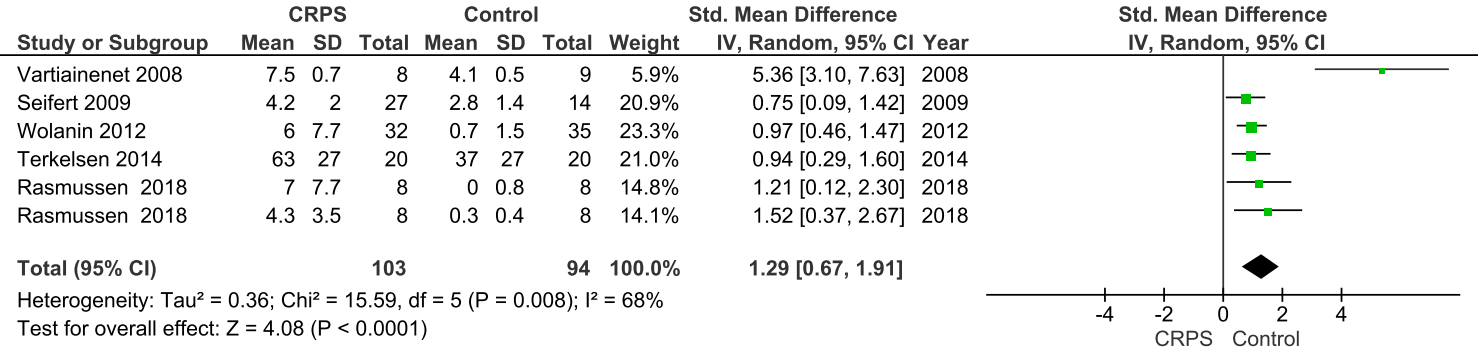

Supplement: Supplementary file 35 — Additional file 35. Fig. S35 Pooled results of pain ratings after noxious stimulus of the affected area. SD: standard deviation, CRPS: complex regional pain syndrome, and Std Mean Difference: standardized mean difference. [file 13018_2022_3461_MOESM35_ESM.pdf]

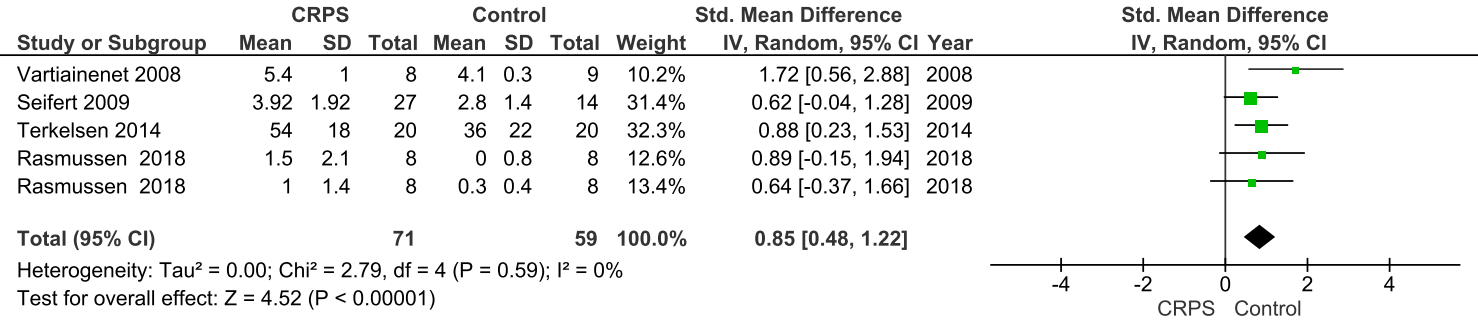

Supplement: Supplementary file 36 — Additional file 36. Fig. S36 Pooled results of pain ratings after noxious stimulus of the remote areas. SD: standard deviation, CRPS: complex regional pain syndrome, and Std Mean Difference: standardized mean difference. [file 13018_2022_3461_MOESM36_ESM.pdf]

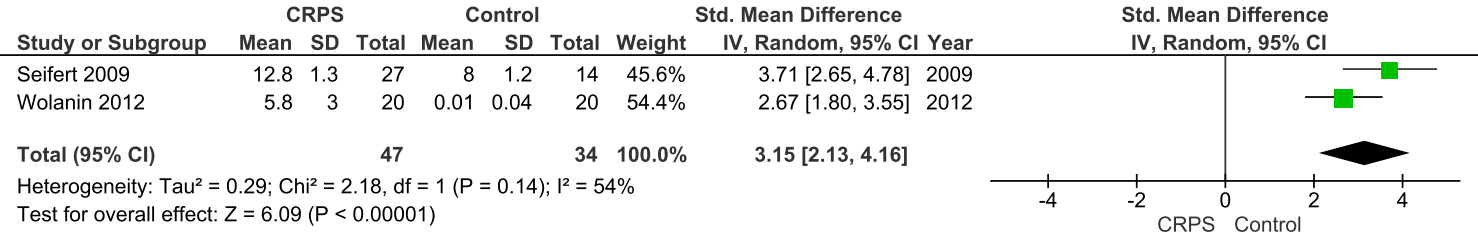

Supplement: Supplementary file 37 — Additional file 37. Fig. S37 Pooled results of area after induced pinprick hyperalgesia of the affected area. SD: standard deviation, CRPS: complex regional pain syndrome, and Std Mean Difference: standardized mean difference. [file 13018_2022_3461_MOESM37_ESM.pdf]

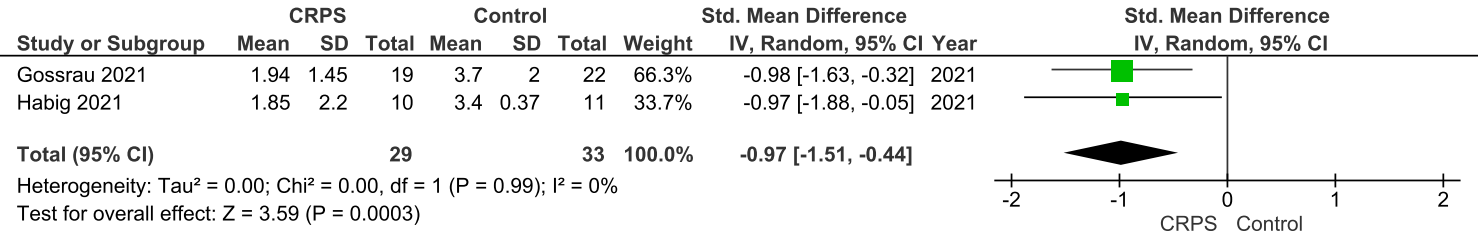

Supplement: Supplementary file 38 — Additional file 38. Fig. S38 Pooled results of pleasantness level of C-tactile perception of the affected area. SD: standard deviation, CRPS: complex regional pain syndrome, and Std Mean Difference: standardized mean difference. [file 13018_2022_3461_MOESM38_ESM.pdf]

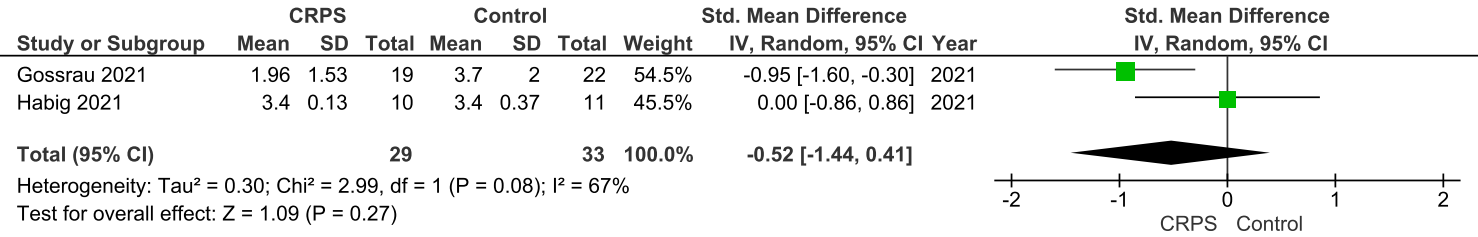

Supplement: Supplementary file 39 — Additional file 39. Fig. S39 Pooled results of pleasantness level of C-tactile perception of the remote areas. SD: standard deviation, CRPS: complex regional pain syndrome, and Std Mean Difference: standardized mean difference. [file 13018_2022_3461_MOESM39_ESM.pdf]

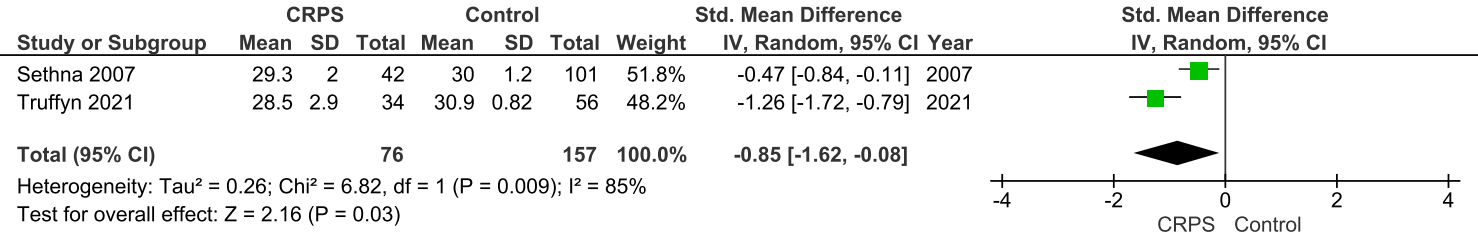

Supplement: Supplementary file 40 — Additional file 40. Fig. S40 Pooled results of cold detection threshold (CDT) of the affected area of children and adolescent with CRPS. SD: standard deviation, CRPS: complex regional pain syndrome, and Std Mean Difference: standardized mean difference. [file 13018_2022_3461_MOESM40_ESM.pdf]

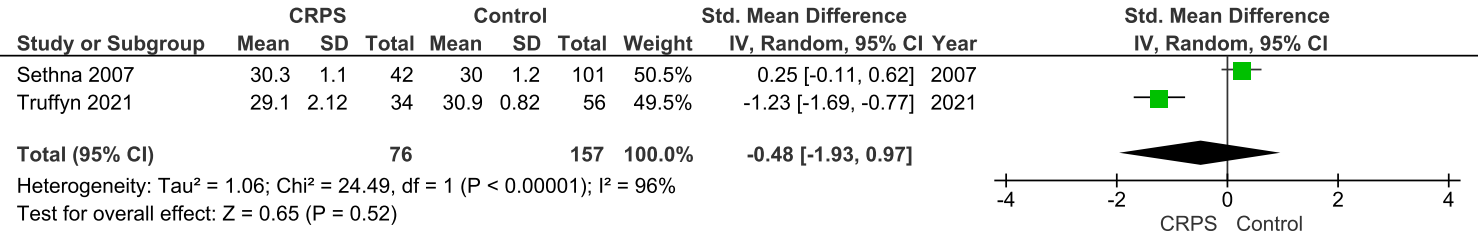

Supplement: Supplementary file 41 — Additional file 41. Fig. S41 Pooled results of cold detection threshold (CDT) of the contralateral side of children and adolescent with CRPS. SD: standard deviation, CRPS: complex regional pain syndrome, and Std Mean Difference: standardized mean difference. [file 13018_2022_3461_MOESM41_ESM.pdf]

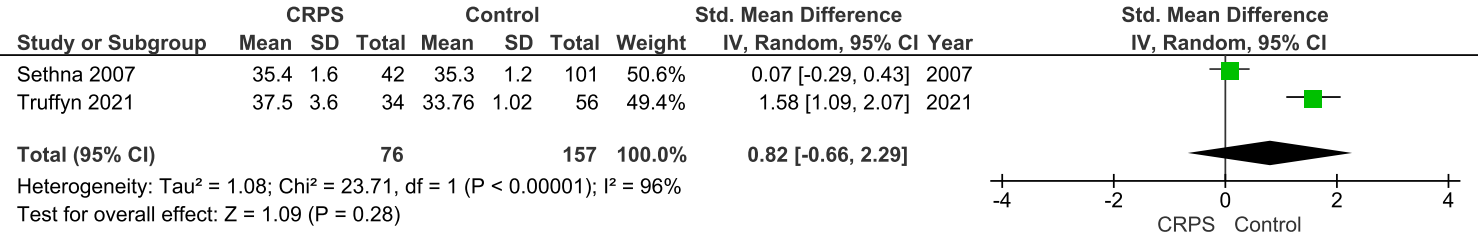

Supplement: Supplementary file 42 — Additional file 42. Fig. S42 Pooled results of warm detection threshold (WDT) of the affected area of children and adolescent with CRPS. SD: standard deviation, CRPS: complex regional pain syndrome, and Std Mean Difference: standardized mean difference. [file 13018_2022_3461_MOESM42_ESM.pdf]

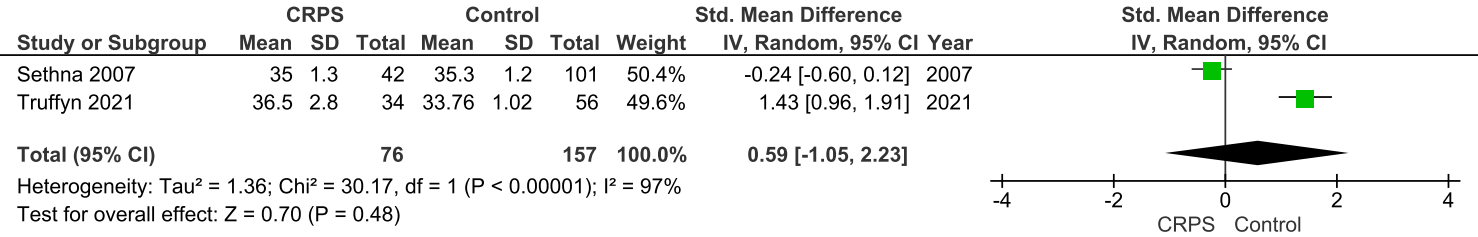

Supplement: Supplementary file 43 — Additional file 43. Fig. S43 Pooled results of warm detection threshold (WDT) of the contralateral side of children and adolescent with CRPS. SD: standard deviation, CRPS: complex regional pain syndrome, and Std Mean Difference: standardized mean difference. [file 13018_2022_3461_MOESM43_ESM.pdf]

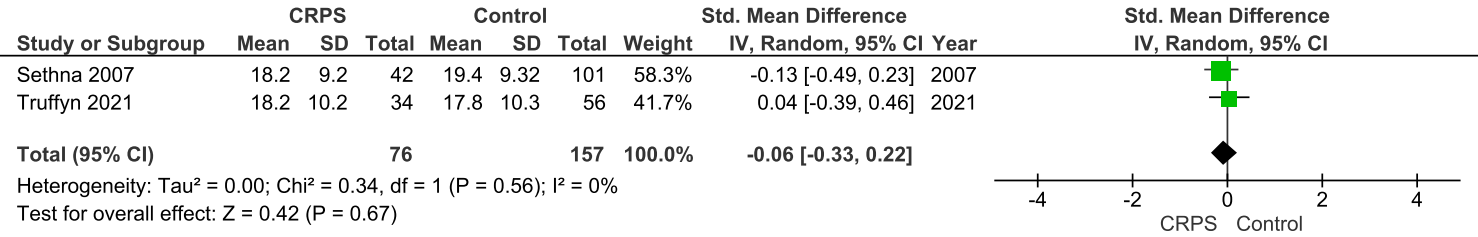

Supplement: Supplementary file 45 — Additional file 45. Fig. S45 Pooled results of cold pain threshold (CPT) of the contralateral side of children and adolescent with CRPS. SD: standard deviation, CRPS: complex regional pain syndrome, and Std Mean Difference: standardized mean difference. [file 13018_2022_3461_MOESM45_ESM.pdf]

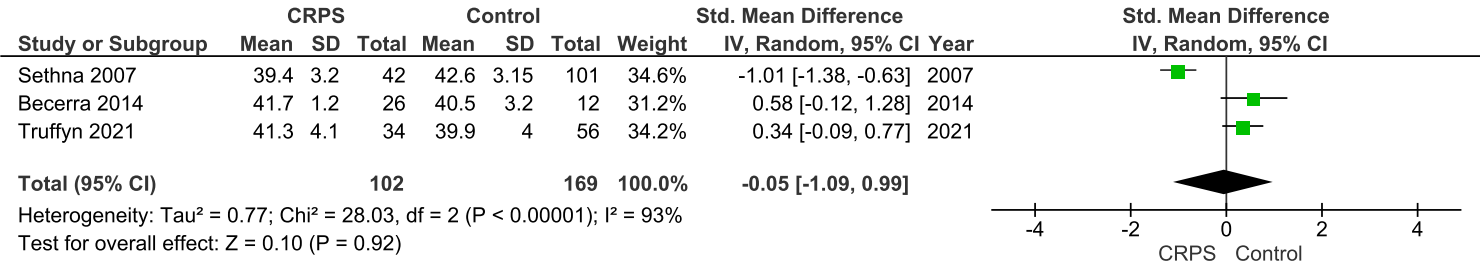

Supplement: Supplementary file 46 — Additional file 46. Fig. S46 Pooled results of heat pain threshold (HPT) of the affected area of children and adolescent with CRPS. SD: standard deviation, CRPS: complex regional pain syndrome, and Std Mean Difference: standardized mean difference [file 13018_2022_3461_MOESM46_ESM.pdf]

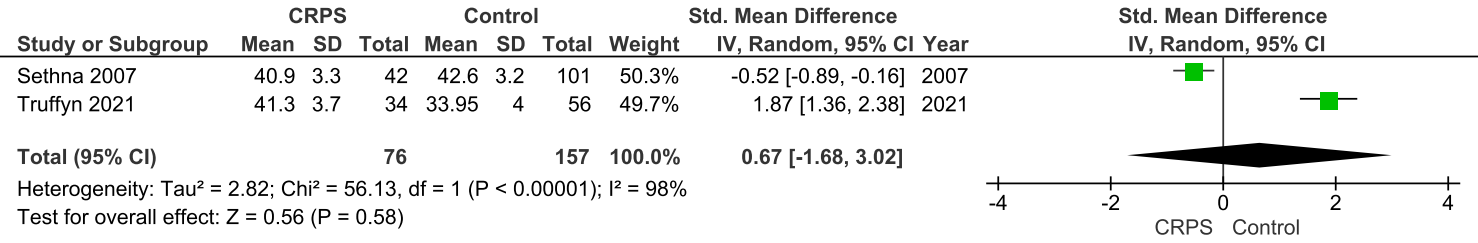

Supplement: Supplementary file 47 — Additional file 47. Fig. S47 Pooled results of heat pain threshold (HPT) of the contralateral side of children and adolescent with CRPS. SD: standard deviation, CRPS: complex regional pain syndrome, and Std Mean Difference: standardized mean difference. [file 13018_2022_3461_MOESM47_ESM.pdf]
