# Supplementary material for: Pain mechanisms in complex regional pain syndrome: a systematic review and meta-analysis of quantitative sensory testing outcomes
Source: J Orthop Surg Res. 2023 Jan 2;18:2. doi: 10.1186/s13018-022-03461-2 (PMC9806919; doi:10.1186/s13018-022-03461-2)

	CRPS			Control				Std. Mean Difference	
Study or Subgroup	Mean	SD	Total	Mean	SD	Total	Weight	IV, Random, 95% CI	Year
Thimineur 1998	3.9	9	140	2.9	0.3	26	19.7%	0.12 [-0.30, 0.54]	1998
Kemler 2000	7.9	255	33	0.07	0.27	50	17.9%	0.05 [-0.39, 0.49]	2000
Kemler 2000	4.5	33.88	20	0.34	1.56	50	12.8%	0.23 [-0.29, 0.75]	2000
Seifert 2009	28	30.9	27	8.14	5.95	14	7.7%	0.76 [0.10, 1.43]	2009
Gierthmühlen 2012	0.32	0.68	257	0.15	0.4	32	25.5%	0.26 [-0.11, 0.63]	2012
Palmer 2019	0.14	0.7	36	0.06	0.09	37	16.4%	0.16 [-0.30, 0.62]	2019
**Total (95% CI)**			**513**			**209**	**100.0%**	**0.21 [0.03, 0.40]**	

Heterogeneity: Tau² = 0.00; Chi² = 3.46, df = 5 (P = 0.63); I² = 0%

Test for overall effect: Z = 2.24 (P = 0.02)

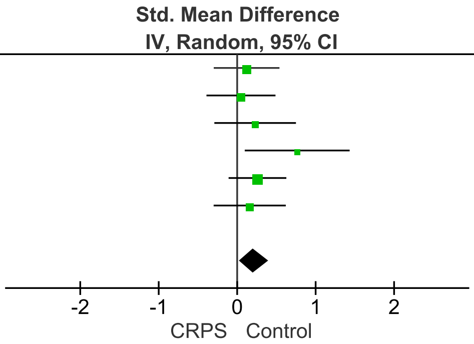

Supplement: Supplementary file 11 — Additional file 11. Fig. S11 Pooled results of mechanical detection threshold (MDT) of the affected area. SD: standard deviation, CRPS: complex regional pain syndrome, and Std Mean Difference: standardized mean difference. [file 13018_2022_3461_MOESM11_ESM.pdf]
