# Supplementary material for: Pain mechanisms in complex regional pain syndrome: a systematic review and meta-analysis of quantitative sensory testing outcomes
Source: J Orthop Surg Res. 2023 Jan 2;18:2. doi: 10.1186/s13018-022-03461-2 (PMC9806919; doi:10.1186/s13018-022-03461-2)

	CRPS			Control				Std. Mean Difference	
**Study or Subgroup**	**Mean**	**SD**	**Total**	**Mean**	**SD**	**Total**	**Weight**	**IV, Random, 95% CI**	**Year**
Seifert 2009	213.6	223	27	310.8	167.3	14	29.5%	-0.46 [-1.12, 0.19]	2009
Gierthmühlen 2012	1.68	0.5	296	1.78	0.35	32	30.0%	-0.20 [-0.57, 0.16]	2012
Wolanin 2012	3.2	0.3	32	10.5	0.13	35	10.9%	-31.71 [-37.27, -26.16]	2012
Kolb 2012	139	163	20	148	184.7	20	29.6%	-0.05 [-0.67, 0.57]	2012
**Total (95% CI)**			**375**			**101**	**100.0%**	**-3.66 [-5.95, -1.37]**	

Heterogeneity: Tau² = 4.51; Chi² = 123.94, df = 3 (P < 0.00001); I² = 98%
Test for overall effect: Z = 3.13 (P = 0.002)

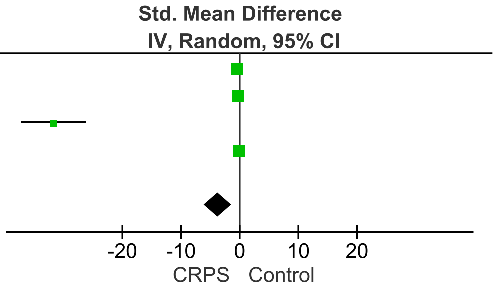

Supplement: Supplementary file 23 — Additional file 23. Fig. S23 Pooled results of mechanical pain threshold (MPT) of the affected area. SD: standard deviation, CRPS: complex regional pain syndrome, and Std Mean Difference: standardized mean difference. [file 13018_2022_3461_MOESM23_ESM.pdf]
