# Supplementary material for: Pain mechanisms in complex regional pain syndrome: a systematic review and meta-analysis of quantitative sensory testing outcomes
Source: J Orthop Surg Res. 2023 Jan 2;18:2. doi: 10.1186/s13018-022-03461-2 (PMC9806919; doi:10.1186/s13018-022-03461-2)

	CRPS			Control				Std. Mean Difference	
**Study or Subgroup**	**Mean**	**SD**	**Total**	**Mean**	**SD**	**Total**	**Weight**	**IV, Random, 95% CI**	**Year**
Sieweke 1999	2.1	5.8	17	1.9	4.8	15	13.4%	0.04 [-0.66, 0.73]	1999
Gierthmühlen 2012	0.4	0.35	257	0.32	0.24	32	20.5%	0.23 [-0.13, 0.60]	2012
Kolb 2012	4	4.7	20	2	1	20	14.6%	0.58 [-0.06, 1.21]	2012
Wolanin 2012	6	7.7	32	1.3	3	35	17.4%	0.81 [0.31, 1.31]	2012
Rooijen 2013	1.9	1.9	17	2	1.5	42	16.0%	-0.06 [-0.62, 0.50]	2013
Rooijen 2013	1.47	0.77	31	2	1.5	42	18.1%	-0.42 [-0.89, 0.05]	2013
**Total (95% CI)**			**374**			**186**	**100.0%**	**0.19 [-0.17, 0.56]**	

Heterogeneity: $Tau^2 = 0.14$; $Chi^2 = 14.84$, df = 5 (P = 0.01); $I^2 = 66\%$

Test for overall effect: Z = 1.03 (P = 0.30)

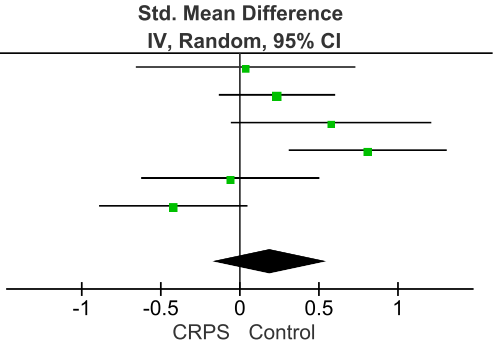

Supplement: Supplementary file 33 — Additional file 33. Fig. S33 Pooled results of wind-up ratio (WUR) of the affected area. SD: standard deviation, CRPS: complex regional pain syndrome, and Std Mean Difference: standardized mean difference. [file 13018_2022_3461_MOESM33_ESM.pdf]
