# Supplementary material for: Pain mechanisms in complex regional pain syndrome: a systematic review and meta-analysis of quantitative sensory testing outcomes
Source: J Orthop Surg Res. 2023 Jan 2;18:2. doi: 10.1186/s13018-022-03461-2 (PMC9806919; doi:10.1186/s13018-022-03461-2)

	CRPS			Control				Std. Mean Difference		Std. Mean Difference
Study or Subgroup	Mean	SD	Total	Mean	SD	Total	Weight	IV, Random, 95% CI	Year	IV, Random, 95% CI
Sethna 2007	25.53	6.2	42	19.4	9.32	101	35.7%	0.71 [0.35, 1.08]	2007	
Becerra 2014	21.2	3.2	26	11.2	2.6	12	28.9%	3.23 [2.20, 4.26]	2014	
Truffyn 2021	18.9	10.3	34	17.8	10.3	56	35.3%	0.11 [-0.32, 0.53]	2021	
**Total (95% CI)**			**102**			**169**	**100.0%**	**1.23 [0.05, 2.41]**		

Heterogeneity: Tau² = 0.98; Chi² = 30.60, df = 2 (P < 0.00001); I² = 93%
Test for overall effect: Z = 2.04 (P = 0.04)

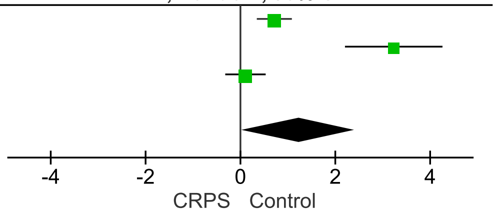

Supplement: Supplementary file 44 — Additional file 44. Fig. S44 Pooled results of cold pain threshold (CPT) of the affected area of children and adolescent with CRPS. SD: standard deviation, CRPS: complex regional pain syndrome, and Std Mean Difference: standardized mean difference. [file 13018_2022_3461_MOESM44_ESM.pdf]
